# GFI1 as a novel regulator of γδ T cell development and the IL-17/IFN-γ lineage commitment

**DOI:** 10.3389/fimmu.2026.1849622

**Published:** 2026-08-05

**Authors:** Jennifer Fraszczak, David Obwegs, Kaifee Arman, Tanja Muralt, Bavanitha Thurairajah, Ève Mallet Gauthier, Irah L. King, Heather J. Melichar, Sagar, Tarik Möröy

**Affiliations:** 1 Institut de recherches cliniques de Montréal (IRCM), Montréal, QC, Canada; 2 Department of Medicine II (Gastroenterology, Hepatology, Endocrinology, and Infectious Diseases), Freiburg University Medical Center, Faculty of Medicine, University of Freiburg, Freiburg, Germany; 3 Faculty of Biology, University of Freiburg, Freiburg, Germany; 4 Meakins-Christie Laboratories, Research Institute of the McGill University Health Centre, Montreal, QC, Canada; 5 Department of Microbiology and Immunology, McGill University, Montreal, QC, Canada; 6 Rosalind and Morris Goodman Cancer Institute of the McGill University, Montreal, QC, Canada; 7 Département de microbiologie, infectiologie et immunologie, Faculty of Medicine, Université de Montréal, Montréal, QC, Canada; 8 Department of Medicine, Division of Clinical and Translational Research, McGill University, Montreal, QC, Canada

**Keywords:** GFI1, gamma delta T cells, pre T cell development, c-MAF, allergy, IL-17

## Abstract

GFI1 is a DNA-binding transcription factor that regulates the commitment of hematopoietic precursors to myeloid and lymphoid lineages. Here we report that GFI1 is expressed in γδ T cells and restricts the cellularity of RORγt^+^Vγ6^+^ γδ T cells that produce high levels of IL-17A while promoting the expansion of Vγ1^+^ and Vγ4^+^ γδ T cells. GFI1 deficiency results in an expansion of Vγ6^+^ γδT17 cells that starts post-birth and is strongly exacerbated upon challenge with an inflammatory allergen. Additionally, we observe an expansion of RORγt^+^/MAF^+^ cells within the thymic DN1e population of GFI1-deficient mice. The DN1e population, along with other DN subsets in GFI1-deficient mice, exhibits a distinctive γδT17 cell-specific transcriptomic profile. Specifically, DN1, DN3, and γδ T cells lacking GFI1 show upregulation of the B-ZIP transcription factor MAF, which regulates genes critical for γδ T cells, such as *IL-17a*, *IL-22*, and *Blk*. The *Maf* gene is occupied by GFI1 in DN pre-T cells at cognate binding sites in its promoter region, suggesting that GFI1 acts as a direct repressor of *Maf*. We conclude that GFI1 functions as a novel regulator of Vγ6^+^ γδT17 precursor cells restricting their peripheral expansion by acting upstream of a MAF-dependent regulatory network.

## Highlights

- GFI1 controls the expansion of γδ T cells in peripheral lymphoid organs.
- GFI1 specifically restricts the expansion of γδ T cells secreting IL-17 by acting upstream of a MAF-dependent transcriptional regulatory network.
- GFI1 plays a cell-intrinsic role in controlling the generation of γδ T cells through the repression of *Maf.*
- GFI1 controls a MAF^+^/RORγt^+^ γδ T cell precursor cell population within the DN1e cell subset.

## Introduction

GFI1 is a 50-kDa transcription factor that contains an N-terminal SNAG domain and six C-terminal C_2_H_2_-type zinc fingers, which facilitate DNA binding to target genes (1–4). The SNAG domain of GFI1 interacts with the histone demethylase KDM1A (LSD1) and recruits it along with CoRest to target genes (5–8). GFI1 and LSD1 are also part of the nucleosome remodeling and deacetylase complex (NuRD) (9, 10), which enables transcriptional repression (5). *Gfi1* was first identified in retrovirally induced murine T cell lymphoma (11, 12). Through the analysis of *Gfi1*-deficient mice (13), it was recognized that GFI1 controls the proliferation of hematopoietic stem cells (14) as well as lymphoid/myeloid (15) and monocyte/granulocyte lineage decisions in progenitor cells (2, 16). Moreover, GFI1 controls pre-T cell numbers in the thymus and the T cell differentiation process which begins at the early thymic progenitor (ETPs) stage. GFI1 is required for the survival and proliferation of the very early DN1 and DN2 pre-T cells, and the absence of GFI1 leads to a block of differentiation and the accumulation of an abnormal population between the DN1 and DN2 stages (17). In addition, GFI1 also plays a role in pre-TCR selection, where it regulates the proliferation and differentiation of DN3 cells into DN4 cells, and it is involved in major histocompatibility complex (MHC)-restricted positive and negative selection processes, highlighting its influence on the development of CD4 and CD8 single-positive T cells (17). GFI1 has been shown to regulate pre-T cell differentiation through NOTCH1 signaling (18), which is essential for the maintenance of ETPs and T cell lineage commitment as well as gene expression of lung Krüppel-like factor (KLF2) or inhibitor of DNA binding 1 and 2 (Id1 and Id2) (17). GFI1 also regulates Foxo1 expression in thymic DP cells to maintain a specific transcriptional program that ensures the formation of mature T cells (19). GFI1 also restrains the generation of regulatory T cells and plays a role in their function (20–22). In mature CD4^+^ T cells, GFI1 is induced to maintain an active Th2 program (22, 23) by either preventing the degradation of GATA3 or blocking the production of IL-17 regulated by RORγt (24–28). GFI1 also inhibits the Th1 program in activated CD4^+^ T cells (29) and modulates Th9 polarization (30). In mature CD8^+^ T cells, GFI1 has recently been shown to control the formation of effector CD8^+^ T cells (31) and the persistence of memory CD8^+^ T cells (32) during infection.

To date, studies characterizing GFI1 function in T cell differentiation or peripheral T cell activity have focused on αβ T cell receptor-expressing populations, while a potential role for GFI1 in γδ T cells has not been investigated. Murine γδ T cells develop in waves starting in the fetal thymus at E14 with Vγ5^+^Vδ1^+^ dendritic epidermal T cells (DETC), followed by Vγ6^+^ and Vγ4^+^ γδ T cell subsets around fetal stage E16 homing to specific tissues like the lungs or the tongue (33). Around birth, while DETC and Vγ6^+^ γδ T cell production declines drastically, new subsets of γδ T cells emerge that seed the intestine, liver, and peripheral lymphoid organs, respectively (34). Different γδ T cell subsets have been identified (34–36), with two main subpopulations being γδT1 cells (37) and γδT17 cells (38). It is known that most γδT17 cells express either the Vγ4 or the Vγ6 chain (39), with Vγ6^+^ γδ T cells being produced almost exclusively at the fetal stage and the Vγ4^+^ γδ T cells at the fetal and perinatal stages (40–42). Recent reports suggest that γδT17 and γδT1 cells can develop in the thymus *via* several pathways: One of these pathways is independent of the TCR and is initiated in two subsets of the DN1 thymic precursors called DN1d and DN1e (43–45). From these subsets, both IFN-γ^+^ and IL-17^+^ γδ T cells can emerge (45). The second pathway involves DN3 cells, where a strong TCR signal favors the IFN-γ-producing lineage, whereas a weak signal is associated with the development of γδT17 lineage (33, 46–49). Each γδ T cell subset has specific functions that are not only critical for the immune innate response against many pathogens (35, 50) but also play a role in autoimmune disorders and inflammation (51–55). Moreover, clinical trials are also currently under way to test whether γδ T cells are effective in cancer immunotherapy (56), creating a need to better understand their development and function. The role that transcription factors play in establishing γδ T cell fate has been challenging, and current knowledge remains limited.

Here we demonstrate that GFI1 is expressed in γδ T cells and that genetic ablation of GFI1 leads to a significant expansion of RORγt^+^Vγ6^+^γδ T cells producing IL-17, with a concomitant decrease in Vγ1^+^ and Vγ4^+^ γδ T cells in adult mice. This bias toward the γδT17 subtype starts after birth in GFI1 knock-out (KO) mice and remains in place in the adult thymus, where a Vγ6^+^ subset secreting IL-17 becomes the predominant population among γδ T cells. This phenotype is exacerbated in *Gfi1*-deficient mice when challenged with the inflammatory antigen. In addition, GFI1 KO mice have an increased number of c-MAF^+^/RORγt^+^ cells in the DN1e subset, which has been described to contain precursors for γδT17 cells (45). *Gfi1*-deficient DN thymocytes and γδ T cells show an upregulation of the B-Zip transcription factor c-MAF, which is known to regulate the production of γδT17 cells, suggesting that GFI1 controls the cellularity of γδ T cells and their progenitors though the regulation of MAF.

## Results

### *Gfi1* deficient mice show an expansion of γδ T cells in barrier tissues and in peripheral lymphoid organs

**Figure 1:**
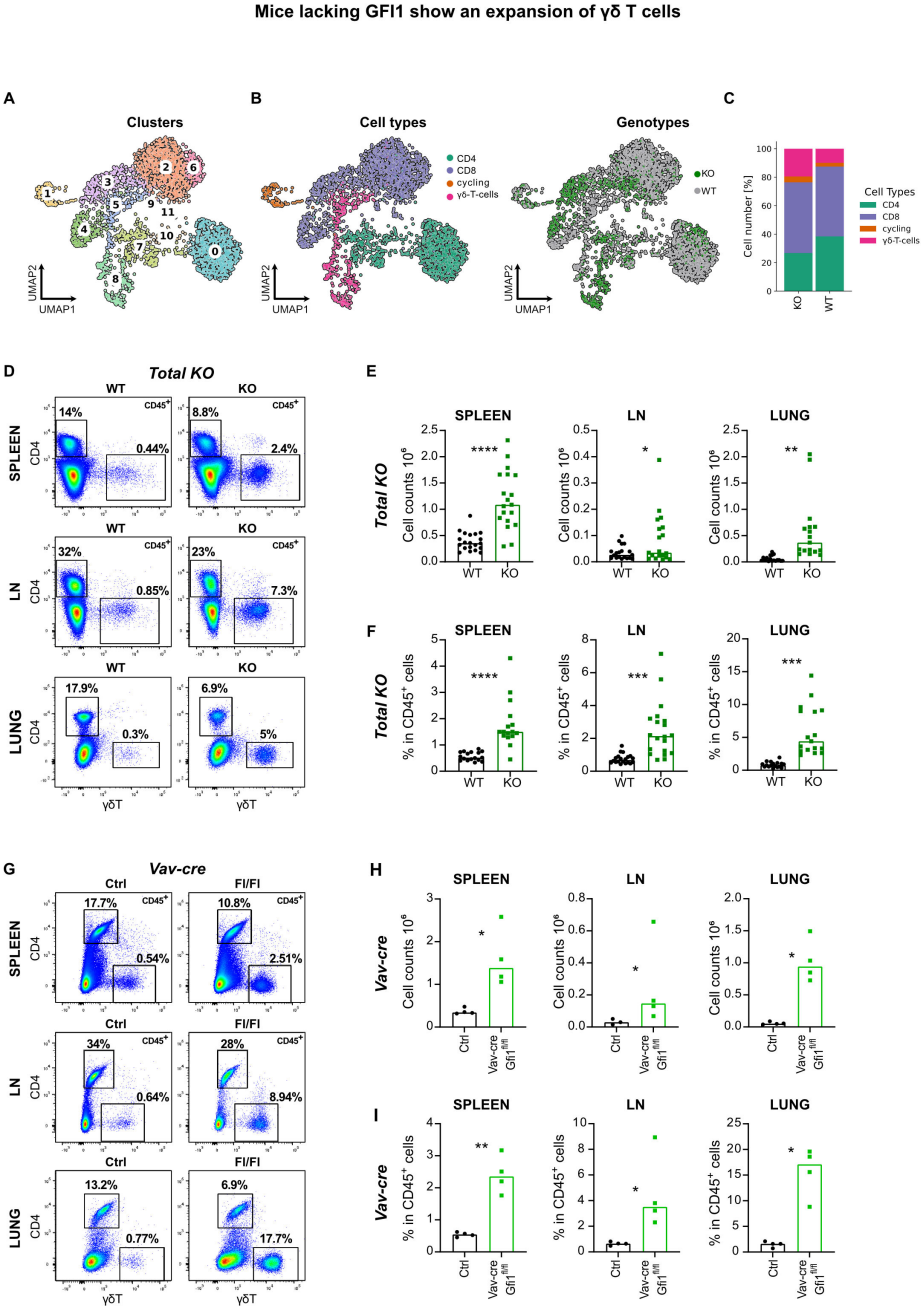
Phenotype of γδ T cells in adult GFI1 KO mice. **(A)** UMAP representation based on gene expression profiling by scRNA-seq depicting 12 clusters in the CD3^+^ lymphocyte compartment in the lung tissue of WT and GFI1 KO mice and the defined subsets and genotypes labeled in different colors **(B)**. **(C)** Bar plots showing the distribution of the defined populations in the indicated genotypes. **(D)** Representative plots of lung, splenic, and LN cells from WT and GFI1 KO mice analyzed by flow cytometry and stained to identify γδ T cells (CD45^+^CD4^-^γδT^+^) after gating on live cells (according to the FSC/SSC) and singlets. **(E, F)** Cell numbers **(E)** and frequencies in CD45^+^ cells **(F)** of γδ T cells (CD4^-^γδT^+^) in the lung, spleen, and LN of WT and GFI1 KO mice. **(G)** Representative plots of lung, splenic, and LN cells from control and *vav-cre GFI1^flox/flox^* mice analyzed by flow cytometry and stained to identify γδ T cells (CD45^+^CD4^-^γδT^+^) after gating on live cells (according to the FSC/SSC) and singlets. **(H, I)** Cell numbers **(H)** and frequencies in CD45^+^ cells **(I)** of γδ T cells (CD4^-^γδT^+^) in the lung, spleen, and LN of control and *vav-cre GFI1^flox/flox^* mice. Adult mice meaning mice older than 5 weeks. Each data point represents one individual mouse [**(E**, **F)**: *n* > 16, three to four independent experiments, **(H, I)**]: *n* = 4, two independent experiments). Statistical testing was performed with paired *t*-test using Prism v9 (GraphPad). *p*-values are shown as *<0.05, **<0.01, ***<0.005, and ****<0.001 where each statistical significance was found.

To gain more insight into the potential role of GFI1 in the immune compartment of barrier tissues, we performed single-cell RNA sequencing (scRNA-seq) of lung cells from GFI1 WT and KO mice. We noticed that the UMAP representation showed two clusters (clusters 5 and 8) of *Cd4^-^*, *Cd8a*^-^, *Cd8b*^-^, and *Trdc*^+^ cells within the *Cd3e*^+^ lymphocyte compartment, which were overrepresented in GFI1 KO mice (**Figures 1A–C**; **Supplementary Figures 1A, B**). These two clusters were annotated as γδ T cells, while all other clusters were conventional CD4^+^ or CD8^+^ T cells (**Figures 1A–C**; **Supplementary Figures 1A, B**). Flow cytometry confirmed the presence of an increased proportion of γδ T cells in the lungs, spleen, and lymph nodes (LN) (**Figures 1D–F**). This observation stood in stark contrast to the number of CD4^+^ T cells, which are known to be reduced in *Gfi1*-deficient mice (17, 18, 57). Accordingly, the proportion of cells from GFI1 KO mice belonging to the CD4^+^ T cell clusters (clusters 7, 10, and 0) was reduced compared to WT mice (**Figure 1A–C**; **Supplementary Figures 1A, B**), which was also seen by flow cytometry (**Supplementary Figure 1C**). The same observations were made with *vav-cre GFI1^flox/flox^* mice where GFI1 is deleted solely in hematopoietic cells. As in the germline GFI1 KO mice, γδ T cells from *vav-cre GFI1^flox/flox^* mice were expanded in number and frequency compared to cre-expressing GFI1 WT mice (**Figures 1G–I**), while CD4^+^ T cells were reduced (**Supplementary Figure 1D**). This excluded the non-hematopoietic environment as a potential cause for γδ T cell expansion in GFI1 KO mice.

#### Specific expansion of γδT17 cells in GFI KO mice

**Figure 2:**
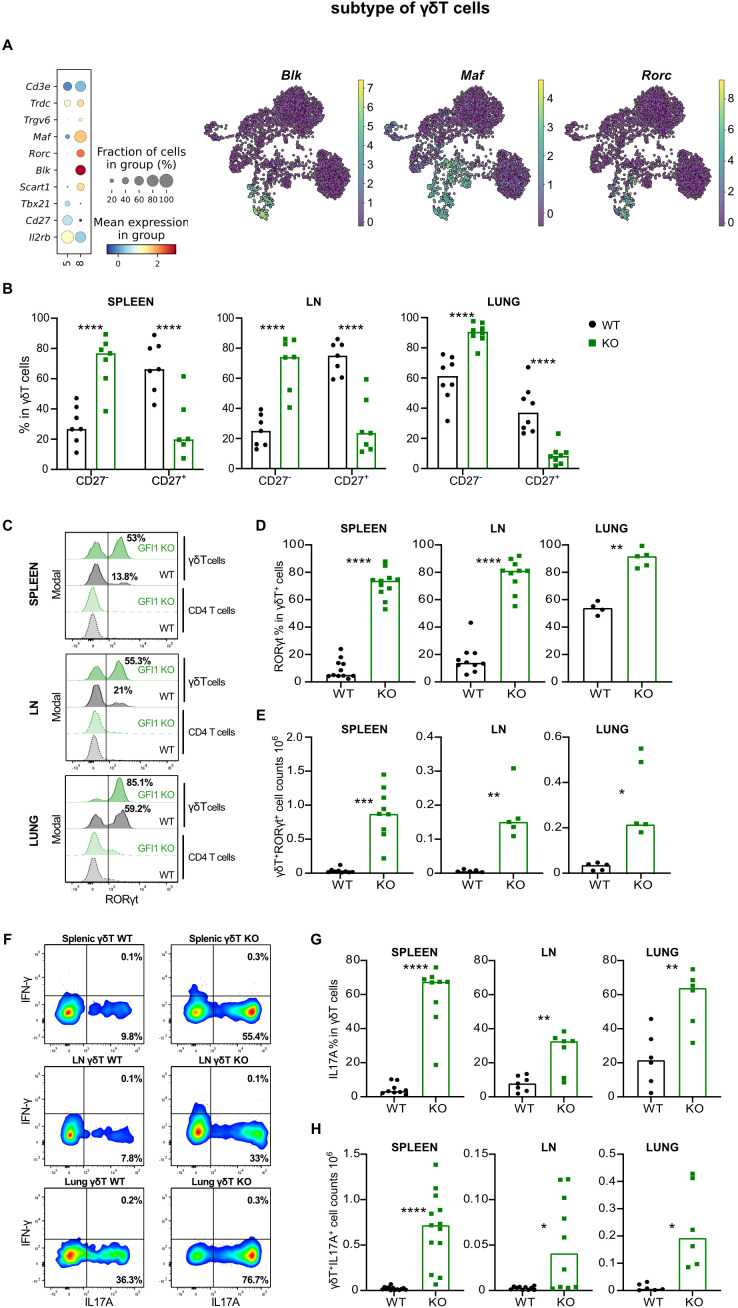
Expansion of the RORγt^+^Il-17a^+^ γδT subset in adult GFI1 KO mice. **(A)** UMAP representation showing the expression of *Blk*, *Maf*, and *Rorc* genes in the CD3^+^ lymphocyte compartment in the lung tissue of WT and GFI1 KO mice. Color represents the normalized expression of the gene. **(B)** Frequencies of CD27^+^ and CD27^-^ cells in splenic, LN, and lung CD45^+^CD4^-^γδT^+^ from WT and GFI1 KO mice. **(C)** Representative normalized histogram of RORγt^+^ cells in either splenic, LN, or lung CD45^+^CD4^-^γδT^+^ and CD45^+^CD4^+^γδT^-^ from WT and GFI1 KO mice. **(D)** Frequencies of RORγt^+^ cells in splenic, LN, and lung CD45^+^CD4^-^γδT^+^ comparing WT and GFI1KO mice. **(E)** Splenic, LN, and lung CD45^+^CD4^-^γδT^+^RORγt^+^ cell counts in WT and GFI1 KO mice. **(F)** Representative plots of lung, splenic, and LN cells from WT and KO mice treated with PMA, ionomycin, and GolgiPlug for 3.5 h and analyzed by flow cytometry. The cells were gated on singlets CD45^+^CD4^-^γδT^+^ before gating for IFN-γ and IL-17A. **(G)** Frequencies of IL-17A^+^ in splenic, LN, and lung CD45^+^CD4^-^ γδT^+^ comparing WT and GFI1 KO mice. **(H)** Cell numbers of splenic, LN, and lung CD45^+^CD4^-^γδT^+^IL-17A^+^ in WT and GFI1 KO mice. Each data point represents one individual mouse [**(B)** n = 7, three independent experiments, **(D)** *n* = 11, three to four independent experiments, **(E)** *n* ≥ 5, two to four independent experiments, **(H)** *n* ≥ 6, three independent experiments). Adult mice meaning mice older than 5 weeks. Statistical testing was performed with multiple paired *t*- test for **(B)** and paired *t*-test for **(D, E)** and **(G, H)** using Prism v9 (GraphPad). *p*-values are shown as *<0.05, **<0.01, ***<0.005, and ****<0.001 where each statistical significance was found.

To determine if the expansion of γδ T cells in GFI1 KO mice was specific to the γδ T cell subtype, we further examined the key features of expanded γδ T cells in GFI1 KO lungs. Expanded lung γδ T cells showed transcriptomic features of γδT17 cells as indicated by the expression of *Blk*, *Maf*, *Rorc*, and *Scart*—in particular, cluster 8 (**Figure 2A**). We analyzed these cells further and found that a large proportion of the expanded γδ T compartment in GFI1 KO lacked CD27 (**Figure 2B**; **Supplementary Figure 2A**), a marker associated with the γδT1 subtype (39). Consistent with this observation, we also found that up to 80% of *Gfi1*-deficient γδ T cells are RORγt^+^ in the lung, spleen, and LN (**Figures 2C–E**). Similar data were obtained with *vav-cre GFI1^flox/flox^* mice, where increased frequencies and numbers of RORγt^+^ γδ T cells were also evident compared to cre-expressing GFI1 WT mice (**Supplementary Figures 2B–D**). The upregulation of RORγt was also seen in *Gfi1*-deficient CD4^+^ T cells from lymph nodes and lung but not from spleen, albeit at a much lower level (**Figure 2C**; **Supplementary Figure 2E**).

To test the functionalities of GFI1 KO γδ T cells, we cultured cells from the spleen, LN, and lung of WT and GFI1 KO mice and treated them with PMA and ionomycin in the presence of GolgiPlug. We found that large proportions of *Gfi1*-deficient γδ T cells from spleen, LN, and lungs produced IL-17A (**Figures 2F, G**), which led to significantly increased numbers of IL-17A^+^ γδ T cells in the KO culture when compared to WT controls (**Figure 2H**). Moreover, GFI1 KO γδ T cells were also able to produce IL-22 (**Supplementary Figures 2F–H**), a cytokine often produced together with IL-17A by γδT17 cells (58). These findings suggest that the absence of GFI1 leads specifically to the expansion of γδT17 cells, which produce IL-17 after stimulation.

### Vγ6-expressing γδT17 cells expand in the absence of GFI1

**Figure 3:**
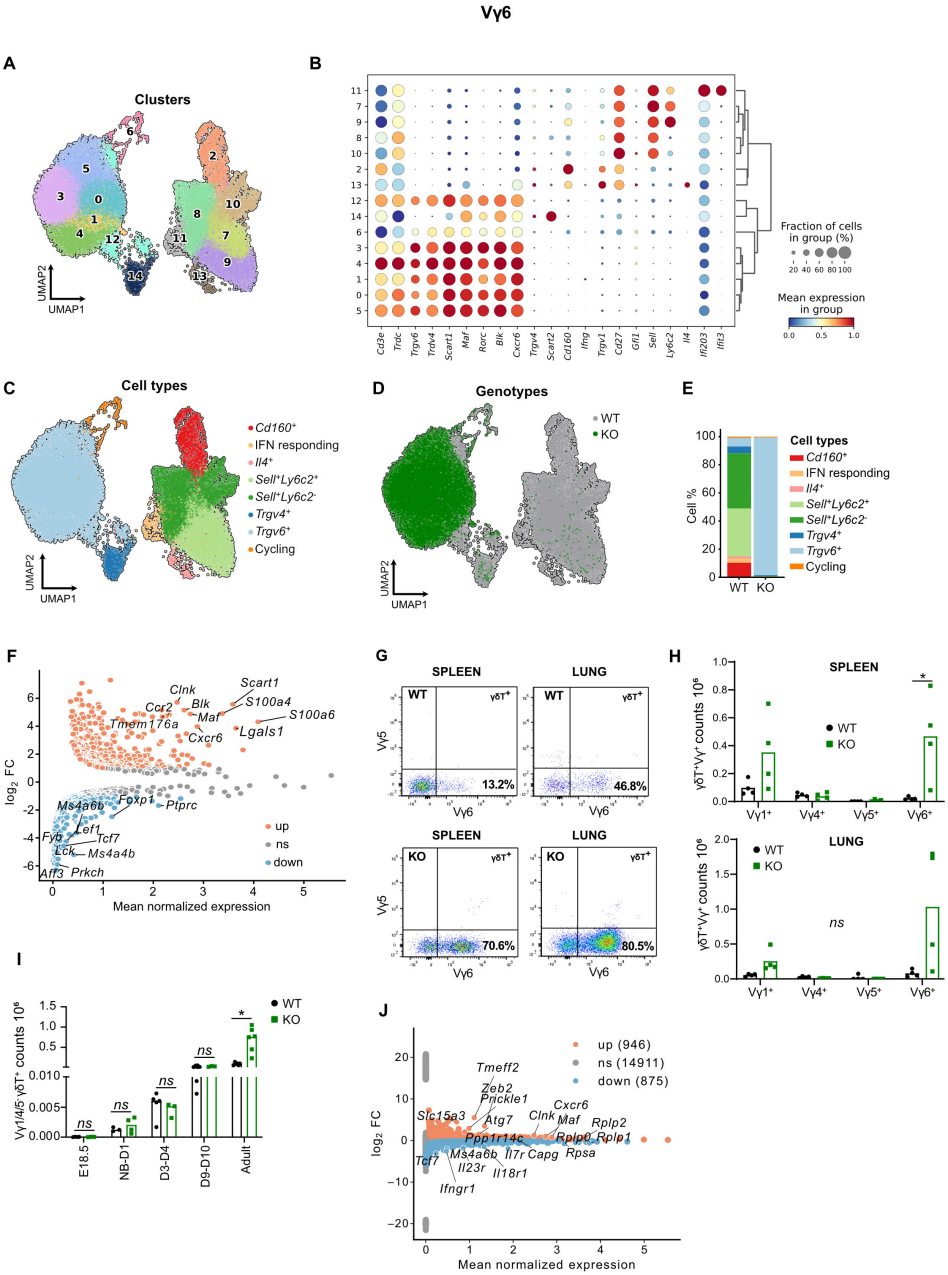
Expansion of Vγ6^+^ γδT17 cells in the periphery of adult GFI1 KO mice. **(A)** UMAP representation based on gene expression profiling by sc-RNA-seq depicting 15 clusters in sorted splenic γδ T cells of WT and GFI1 KO mice. **(B)** Dot plot displays the mean expression level of selected marker genes across the identified clusters shown in **(A)**. Dot size indicates the fraction of cells within a given cluster expressing the respective gene, whereas color intensity reflects the mean normalized expression level of that gene within the cluster. The plot does not represent differential expression between GFI1 KO and WT cells. **(C, D)** UMAP representation of sorted splenic γδ T cells of WT and GFI1 KO mice colored by cell type **(C)** or genotype **(D)**. **(E)** Bar plots showing the distribution of cell types found in splenic γδ T cells in the indicated genotypes. **(F)** Differential gene expression showing key up- or downregulated genes in GFI1 KO splenic γδ T cells versus WT splenic γδ T cells. **(G)** Representative plots of Vγ5^+^ and Vγ6^+^ in γδT^+^ cells from WT and GFI1 KO spleen and lung after gating on live cells (according to FSC/SSC) and singlets. **(H)** Vγ1^+^, Vγ4^+^, Vγ5^+^, or Vγ6^+^ γδT^+^ cell counts in the spleen and lung of adult WT and GFI1 KO mice. **(I)** Cell numbers of Vγ1^-^Vγ4^-^Vγ5^-^ γδT^+^ cells at different time points in WT and GFI1 KO spleen, with adult mice meaning animals older than 5 weeks. **(J)** Differential gene expression showing key up- or downregulated genes in GFI1 KO splenic *Trgv6^+^* γδ T cells versus WT splenic γδ T cells. Each data point in **(H, J)** represent one mouse. **(H)** *n* = 4, two independent experiments, **(I)** *n* = 6, three independent experiments. Statistical testing was performed with multiple Mann–Whitney tests using Prism v9 (GraphPad). *p*-values are shown as *<0.05 where each statistical significance was found.

Flow cytometric analyses of cells from a GFI1 reporter mouse which carries an EGFP cDNA “knock-in” in the *Gfi1* locus, placing it under the control of the *Gfi1* promoter (59), indicated that *Gfi1* is expressed at comparable levels in different Vγ subtypes (**Supplementary Figures 3A, B**). To characterize further the subtype of γδT17 cells, we performed a scRNA-seq analysis on sorted γδ T cells from spleens of WT and GFI1 KO mice. We annotated the clusters based on our previously published study (60), and 15 clusters were identified in splenic γδ T cells (**Figure 3A**; **Supplementary Figure 3C**), with six clusters (0, 1, 3, 4, 5, and 12) expressing genes associated with the Vγ6^+^ γδ T cell subtype such as *Trgv6*, *Cxcr6, Scart1*, and *Rorc* (61) (**Figures 3B, C**). These six clusters represented almost the totality of the splenic γδ T cells sorted from GFI1 KO mice (**Figures 3C–E**). We have included *Ifng*, *Trgv1*, and *Cd27* in the panel since they are established markers of IFN-γ-producing γδ T cells. These markers co-localize with *Trgv1*-expressing clusters and are largely absent from the *Maf^+^Rorc^+^Scart1^+^* Vγ6-associated clusters (**Figure 3B**).

Consistent with the previous observations, we found that the most upregulated genes in GFI1 KO splenic γδ T cells compared to WT controls were associated with the mature Vγ6^+^ γδT17 cells like *Scart1*, *Maf*, *Blk*, and *Ccr2* (61), while genes associated with exchanging and adaptive-like γδ T cells such as *Tcf7* and *Lef1* were downregulated (**Figure 3F**), which was in agreement with the expansion of Vγ6^+^ γδ T cells in the spleen and in the lung of GFI1 KO mice compared to WT controls as determined by flow cytometry (**Figures 3G, H**). Unexpectedly, the expansion of Vγ6^+^ γδ T cells in the spleen started only after birth (**Figure 3I**), at a point in development when the generation of this subtype is not supposed to be maintained, suggesting that GFI1 controls and possibly restricts the expansion of functional Vγ6^+^ γδT17 cells only in adult mice in the periphery.

To further explore the transcriptional consequences of GFI1 deficiency, we performed differential expression analysis within individual γδ T-cell subsets (Vγ1, Vγ4, and V6). While transcriptional changes were detected in all γδ T-cell populations, the magnitude of the effect varied substantially. *Trgv6^+^* cells exhibited the strongest response to GFI1 deficiency, with 1,821 differentially expressed genes (**Supplementary Figure 3J**; **Supplementary Table 1**), compared with 372 and 31 genes in *Trgv1^+^* and *Trgv4^+^* cells, respectively (**Supplementary Figures 3F, G**; **Supplementary Tables 2**, **3**). Importantly, *Trgv6^+^* cells showed altered expression of multiple genes associated with the γδT17 program, including *Maf*, *Rorc*, *Blk*, and *Cxcr6*, indicating that GFI1 not only regulates the size of the Vγ6 compartment but also influences its transcriptional program (**Figure 3J**). In contrast, although *Trgv1^+^* cells displayed a substantial number of differentially expressed genes, these changes were not accompanied by a coordinated induction of canonical γδT17-associated genes (**Supplementary Figure 3F**).

To address the biological significance of the six Vγ6-associated clusters identified by unsupervised clustering, we performed a more detailed analysis of the Vγ6 compartment. While all clusters shared the core Vγ6 signature, the clusters displayed distinct transcriptional states characterized by the differential expression of proliferation-associated, interferon-responsive, and Tcf7/Il7r-associated gene programs (**Supplementary Figure 3E**), suggesting that GFI1 broadly affects the Vγ6 compartment rather than a single Vγ6 subpopulation (**Supplementary Figures 3D, E**).

### Defects in γδ T cell production are detected in the thymus of adult mice

**Figure 4:**
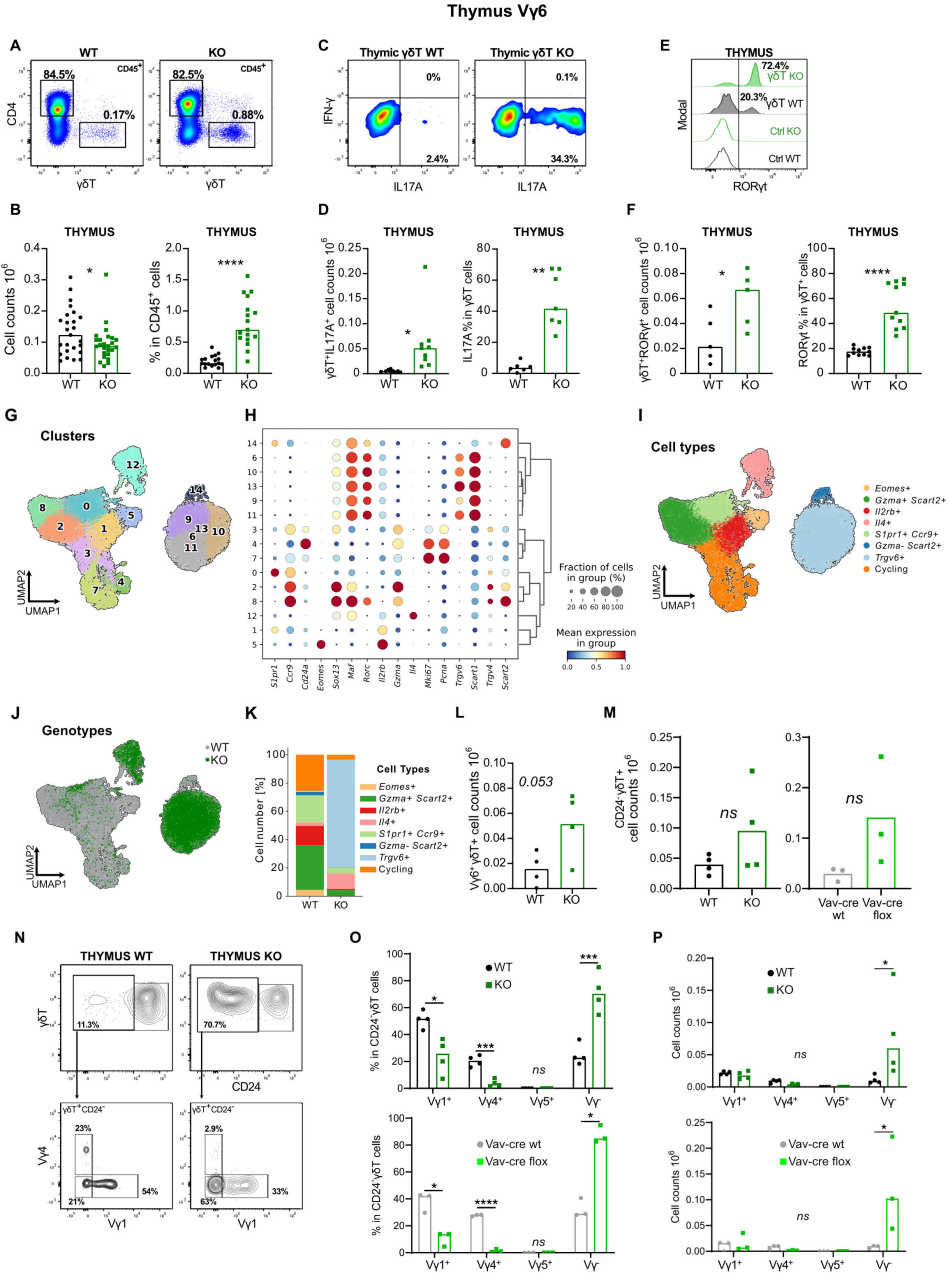
Expansion of Vγ6^+^ γδT17 cells in the thymus of adult GFI1 KO mice. **(A)** Representative plots of thymic CD45^+^CD4^-^γδT^+^ cells in WT and GFI1 KO mice after gating on live cells (according to FSC/SSC) and singlets. **(B)** γδ T cell counts (CD45^+^CD4^-^γδT^+^, left) and frequencies in CD45^+^ cells (CD4^-^γδT^+^, right) in WT and GFI1 KO thymus. **(C)** Representative plots of thymic γδ T cells (CD45^+^CD4^-^γδT^+^) treated with PMA, ionomycin, and GolgiPlug for 3.5 hours and stained for IL-17A and IFN-γ. The cells were gated on singlets CD45^+^CD4^-^γδT^+^ before gating for IFN-γ and IL-17A. **(D)** CD45^+^CD4^-^γδT^+^IL-17^+^ cell counts (left) and IL-17A^+^ cell frequencies in CD45^+^CD4^-^γδT^+^ (right) in WT and GFI1 KO thymus. **(E)** Representative normalized histogram for RORγt and control isotype (Ctrl) in CD45^+^CD4^-^γδT^+^ from WT and GFI1 KO thymic cells. **(F)** CD45^+^CD4^-^γδT^+^RORγt^+^ cell counts (left) and RORγt^+^ cell frequencies in CD45^+^CD4^-^γδT^+^ (right) in WT and GFI1 KO thymus. **(G)** UMAP representation based on gene expression profiling depicting 15 clusters in sorted thymic γδ T cells of WT and GFI1 KO mice. **(H)** Dot plot showing the key differentially expressed genes in each cluster shown in **(G)**. Color represents the scaled mean expression of the gene in the respective cluster, and dot size represents the fraction of cells in the cluster expressing the gene. **(I, J)** UMAP representation of sorted thymic γδ T cells of WT and GFI1 KO mice colored by cell type **(I)** or genotype **(J)**. **(K)** Bar plots showing the distribution of each cell type found in thymic γδ T cells in the indicated genotypes. **(L)** Thymic Vγ6^+^ γδT^+^ cell counts in adult WT and GFI1 KO mice based on cytometry staining. **(M)** Cell numbers of CD24^-^ γδT^+^ in adult thymus of WT and GFI1 KO mice or control (*vav-cre GF1^wt/wt^*) and *vav-cre GFI1^flox/flox^* mice. **(N)** Representative plots of CD24 expression in thymic γδ T cells and Vγ chains expression in thymic CD24^-^ γδ T cells from adult WT and GFI1 KO mice. **(O, P)** Vγ chain frequencies **(O)** and Vγ chain cell counts **(P)** in thymic CD24^-^ γδ T cells from adult WT and GFI1 KO mice or control (*vav-cre GF1^wt/wt^*) and *vav-cre GFI1^flox/flox^* mice. Each data point represents one individual mouse [**(B)** *n* = 26, seven to eight independent experiments, **(D)** *n* = 9, three to four independent experiments, **(F)** *n* = 5, two independent experiments, **(L, M)** *n* = 4, two independent experiments, **(O, P)** *n* = 3–4, one experiment] Adult mice meaning mice older than 5 weeks. Statistical testing was performed with paired *t*-test or multiple paired *t*-test using Prism v9 (GraphPad). *p*-values are shown as *<0.05, **<0.01, ***<0.005, and ****<0.001 where each statistical significance was found.

To determine whether the γδ T cell phenotype observed in GFI1 KO mice was due to a developmental defect, we analyzed γδ T cells from the thymus, where γδ T cells originate and diversify (62). Even though absolute numbers of γδ T cells were similar in GFI1 KO and WT adult thymus, both the number and frequency of IL-17A^+^/RORγt^+^ cells were significantly increased in *Gfi1*-deficient mice compared to WT controls (**Figures 4A–F**). Similar data were obtained again with *vav-cre Gfi1^fl/fl^* mice (**Supplementary Figures 4A–C**). scRNA-seq analysis of sorted thymic γδ T cells from WT and GFI1 KO mice revealed 15 clusters, with five clusters (clusters 6, 9, 10, 11, and 13) expressing genes specific to the Vγ6^+^ γδT17 subtype such as *Scart1*, *Rorc*, *Maf*, and *Trgv6*, and with one cluster (cluster 14) expressing *Rorc, Maf* and *Trgv4* and genes specific to Vγ4^+^ γδT17 cell subtype (**Figures 4G–I**; **Supplementary Figure 4D**) (61, 63). Clusters 6, 9, 10, 11, and 13 form a larger cluster containing only Vγ6^+^ γδT17 cells identified as *Trgv6^+^*, and cluster 14 contains only Vγ4^+^ γδT17 cells identified as *Trgv4^+^* (**Figure 4I**). The partial detection of *Trgv4* (**Figure 4H**) is consistent with dropout and technical limitations owing to the use of a 3′-based scRNA-seq approach. Cluster annotation was therefore based on the overall transcriptional identity, and therefore cluster 14 was assigned to the Vγ4^+^ γδT17 subtype based on its transcriptional profile (e.g., *Maf*, *Rorc*, and *Scart2*). The low detection of *Trgv4* and *Trgv6* transcripts is expected, which does not reliably capture TCR variable regions.

We noted that *Trgv4* transcripts are also detected in clusters 2 and 8 (**Figure 4H**). However, since *Trgv4* expression is often sparsely detected in 3′ scRNA-seq datasets, we also considered the overall transcriptional identity and developmental state of the cells. Clusters 2 and 8 express genes such as *Gzma* and *Ccr9* (**Figure 4H**) and localize within the CD24-associated compartment. Previous studies, including our own work, have shown that these populations represent immature thymic γδ T-cell states that do not correspond to mature peripheral γδT17 cells (63, 64). In contrast, cluster 14 was grouped together with the Vγ6-associated γδT17 clusters since it expressed the canonical γδT17-associated genes *Maf* and *Rorc* together with *Scart2* (**Figure 4H**), which are well-established markers associated with Vγ4^+^ γδT17 cells (63). Hence, cluster 14 was annotated as a Gzma^−^Scart2^+^ population likely representing Vγ4+ γδT17 cells.

Interestingly, the cells found in the *Trgv6^+^* cluster were exclusively from GFI1 KO mice and represented the majority of its thymic γδ T population, while the *Trgv4^+^* cluster was only found in WT thymus (**Figures 4I–K**). We also noted a vast expansion of *IL4^+^* γδ T population in KO thymus. Consistent with these data, the most upregulated genes in GFI1 KO thymic γδ T cells were again genes associated to the Vγ6 subset such as *Scart1* and *Cxcr6* (**Supplementary Figure 4E**), which is consistent with the over-representation of the Vγ6 γδT17 subtype already seen in the thymus of GFI1 KO mice. This bias toward the Vγ6 γδ T subset in adult GFI1 KO thymus was also confirmed by flow cytometry by using the specific antibody against the Vγ6 chain showing a strong tendency to higher numbers of Vγ6^+^ γδ T cells found in adult thymocytes lacking GFI1 compared to WT thymus (**Figure 4L**). Similarly, CD24^-^ γδ T cell numbers in adult thymocytes lacking GFI1 are clearly increased, but the difference did not reach significance compared to controls (**Figure 4N**). Including a CD24 marker also rendered the gating on Vγ chain populations more obvious in *Gfi1*-deficient γδ T cells (**Figure 4N**). We could confirm again a higher proportion and cell count of Vγ1^-^Vγ4^-^Vγ5^-^ (most likely meaning Vγ6^+^) cells in CD24^-^γδ T cells lacking GFI1 in adult mice (**Figures 4N–P**). The bias was also confirmed by staining for CD44 and CD45RB (33, 47), known to distinguish the different subsets of γδ T cells, with significantly increased numbers of CD44^+^CD45RB^-^ cells (known as γδT17) within the CD24^-^ γδ T cell subset in GFI1 KO thymus (**Supplementary Figures 4F, G**). As observed in the spleen and lungs, the over-representation of Vγ6^+^ γδ T cells in the GFI1 KO thymus also started after birth (**Supplementary Figures 4H–K**) with higher frequencies of Vγ1^-^Vγ4^-^Vγ5^-^ (most likely meaning Vγ6^+^) cells in CD24^-^γδ T cells of the GFI1 KO young mice and a slightly but significantly increased number of this population at days 8–10, also seen at D18–20 after birth. These data suggested that the absence of GFI1 leads to a γδ T cell developmental defect with biased production of Vγ6^+^ γδ T cells over the other γδ T cell subtypes.

### Evidence for the cell-intrinsic role of GFI1 in γδ T cells

**Figure 5:**
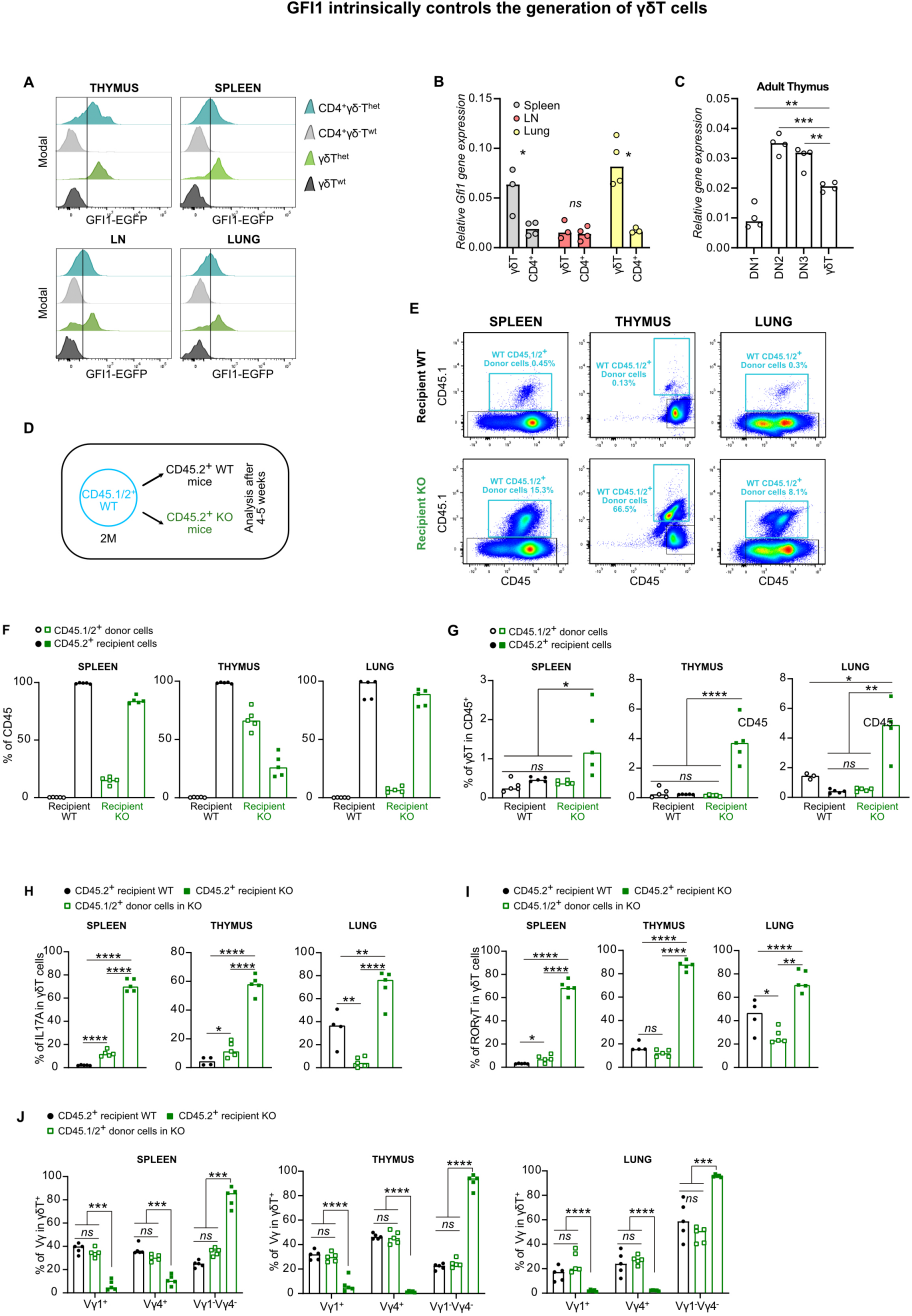
GFI1 intrinsically controls the generation of γδ T cells. **(A)** GFP^+^ cells indicating *Gfi1* expression in either γδ T cells (CD45^+^CD4^-^γδT^+^) or CD4^+^ T cells (CD45^+^CD4^+^γδT^-^) from spleen, LN, lung, and thymus from GFI1:GFP reporter mice (59) (het) and controls (wt). **(B, C)** Relative *Gfi1* expression in γδ T and CD4^+^ T cells from spleen, LN, and lung **(B)** and in thymic γδ T, DN1, DN2, and DN3 cells **(C)** measured by RT-PCR. *Gapdh* was used as a housekeeping gene. **(D)** Scheme representing the transplantation strategy of CD45.1/2^+^ WT BM cells into either CD45.2^+^ WT or GFI1 KO recipient mice. **(E)** Representative plots showing the CD45.1/2^+^ donor cells in the spleen, thymus, and lung of non-irradiated WT and KO recipients. **(F)** Frequencies measured by flow cytometry of either WT CD45.1/2^+^ donor cells or CD45.2^+^ recipient cells in the spleen, lung, and thymus of transplanted WT and GFI1 KO recipient mice. **(G)** Frequencies measured by flow cytometry of either WT donor γδ T cells (in CD45.1/2^+^ cells) or endogenous recipient γδ T cells (in CD45.2^+^ cells) in the spleen, lung, and thymus of transplanted WT and GFI1 KO recipient mice. **(H)** IL-17A frequencies in either donor WT γδ T cells (CD45.1/2^+^ cells) or endogenous recipient γδ T cells (in CD45.2^+^ WT or in CD45.2^+^ GFI1 KO cells) in the spleen, lung and thymus of transplanted WT and GFI1 KO recipient mice. **(I)** RORγt frequencies in either WT donor γδ T cells (CD45.1/2^+^ cells) or endogenous recipient γδ T cells (in CD45.2^+^ WT or in CD45.2^+^ GFI1 KO cells) in the spleen, lung and thymus of transplanted WT and GFI1 KO recipient mice. **(J)** Vγ1^-^Vγ4^-^ frequencies in either WT donor γδ T cells (CD45.1/2^+^ cells) or endogenous recipient γδ T cells (in CD45.2^+^ WT or in CD45.2^+^ GFI1 KO cells) in the spleen, thymus, and lung of transplanted WT and GFI1 KO recipient mice. Each data point represents one individual mouse [**(F–J)** *n* = 5, two independent experiments). Statistical testing was performed with multiple paired *t*- test using Prism v9 (GraphPad). *p*-Values are shown as *< 0.05, **< 0.01, ***< 0.005, and ****< 0.001 where each statistical significance was found.

To determine whether the loss of GFI1 can directly impact γδ T cell development, we compared the GFI1 expression levels in different organs. Flow cytometric analyses of cells from the GFI1 reporter mouse (59) indicated that γδ T cells from spleen, LN, thymus, and lung express higher levels of *Gfi1* than conventional CD4^+^ T cells (**Figure 5A**). Additionally, a higher expression of *Gfi1* mRNA in sorted splenic and lung γδ T cells compared to naïve CD4^+^ T cells, where GFI1 is poorly expressed (57, 59), was also confirmed by q-RT-PCR (**Figure 5B**). *Gfi1* expression in thymic γδ T cells was even higher than in DN1 but lower than in DN3 pre-T cells (**Figure 5C**), while *Gfi1b* mRNA, which was used as a negative control, was not detected in γδ T cells (**Supplementary Figure 5A**).

Next, we wished to determine whether the bias toward the γδT17 subtype in GFI1 KO mice was a consequence of the specific environment created by the absence of GFI1 in the hematopoietic compartment. To clarify this, we transplanted CD45.1/2^+^ WT bone marrow (BM) cells (65, 66) into non-irradiated CD45.2^+^ GFI1 KO mice and analyzed the animals after 4 to 5 weeks. This enabled us to study the impact of the GFI1 KO environment on WT γδ T cells generated during adulthood (**Figure 5D**). We were able to identify a small pool (between 15% and 20%) of WT hematopoietic cells identified as CD45.1/2^+^ cells among CD45.2^+^ GFI1 KO-deficient cells representing 80%–85% of the hematopoietic cells in the spleen and lung of the recipients (**Figure 5F**). As a control, we also transplanted CD45.1/2^+^ WT BM cells into non-irradiated CD45.2^+^ WT recipient mice. However, as expected, the frequencies of CD45.1/2^+^ WT donor cells in non-irradiated CD45.2^+^ WT recipients were very low in the organs analyzed (**Figures 5E,F**). The frequencies of WT donor splenic γδ T cells in GFI1 KO and WT recipients were similar, while the frequencies of the endogenous GFI1 KO host γδ T cells were still elevated compared to WT donor or recipient γδ T cells (**Figure 5G**; **Supplementary Figure 5B**). We obtained the same results in lungs and thymus of the GFI1 KO recipient mice when they were transplanted with WT BM cells (**Figure 5G**; **Supplementary Figure 5B**). We found that the splenic, thymic, and lung WT γδ T cells in the KO recipient mice did not show an overexpression of RORγt or overproduction of IL-17A when compared to the endogenous GFI1 KO host γδ T cells (**Figures 5H, I**; **Supplementary Figures 5C, D**), even though a slight increase of IL-17A and RORγt could be seen compared to recipient WT γδ T cells in the spleen (**Figures 5H, I**; **Supplementary Figures 5C, D**). Moreover, the distribution of the different Vγ chain subtypes was similar between the WT γδ T donors in the GFI1 KO recipient and the WT γδ T cells in all analyzed organs (**Figure 5J**; **Supplementary Figure 5E**). This suggested that the GFI1 KO environment has no or very little effect on γδ T cells and cannot explain the preferential bias toward the γδT producing IL-17 in *Gfi1*-deficient mice. However, further experiments are required to definitively prove a cell-intrinsic role of GFI1 in γδ T cells.

### Identification of a potentially precursor population in adult GFI1 KO mice

**Figure 6:**
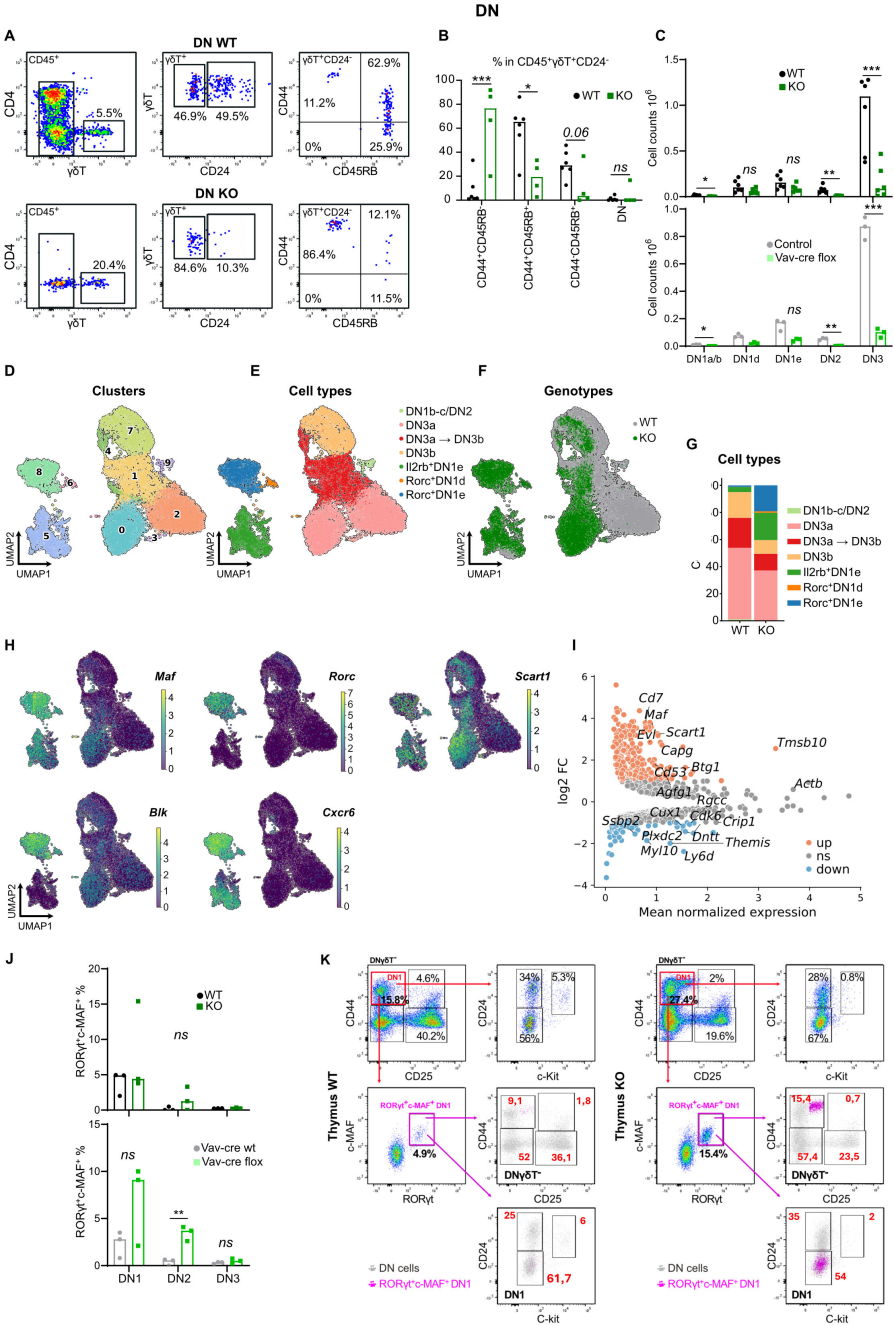
Precursors in thymus of adult mice. **(A)** Representative plots of WT or GFI1 KO thymic DN progenitors cultured on OP9-DL1 in the presence of FLT3 and IL7 for 12–14 days. After gating on singlets CD45^+^γδT^+^CD24^-^ cells, mature CD24^-^ γδT subsets were distinguished according to the expression of CD44 and CD45RB. **(B)** Frequencies of each indicated subset from **(A)**. **(C)** DN cell counts in the thymus of WT and GFI1 KO mice or control (*vav-cre GF1^wt/wt^*) and *vav-cre GFI1^flox/flox^* mice. DN1a/b cells were characterized as CD4^-^CD8^-^γδT^-^CD44^+^CD25^-^CD24^+^Ckit^+^, DN1d cells were characterized as CD4^-^CD8^-^γδT^-^CD44^+^CD25^-^CD24^+^Ckit^-^, DN1e cells were characterized as CD4^-^CD8^-^γδT^-^CD44^+^CD25^-^CD24^-/low^Ckit^-^, DN2 cells were characterized as CD4^-^CD8^-^γδT^-^CD44^+^CD25^+^, and DN3 cells were characterized as CD4^-^CD8^-^γδT^-^CD44^-^CD25^+^. **(D–F)** UMAP representation based on gene expression profiling of pooled DN1, DN2, and DN3 from WT and GFI1 KO mice colored by cluster [10 clusters, **(D)**], cell type **(E)**, and genotype **(F)**. **(G)** Bar plots showing the distribution of each cell type found in the pool of DN1, DN2, and DN3 cells in the indicated genotypes. **(H)** UMAP representation showing the expression of *Maf*, *Rorc*, *Blk*, and *Cxcr6* genes. Color represents the normalized expression of the gene. **(I)** Differential gene expression showing key up- or downregulated genes in GFI1 KO DN1–3 cells versus WT DN1–3 cells. **(J)** Frequencies of c-MAF^+^RORγt^+^ cells in DN1, DN2, and DN3 cells from WT and GFI1 KO mice or control and *vav-cre GFI1^flox/flox^* mice. **(K)** Representative plots showing in pink c-MAF^+^RORγt^+^DN1 cells in the DN1 subsets characterized by the expression of c-Kit and CD24 after gating on Lin^-^ CD4^-^CD8^-^γδT^-^CD44^+^CD25^-/low^ from WT and GFI1 KO thymus. (Lin^-^ = CD19, CD11b, CD11c, NK1.1). Adult mice meaning mice older than 5 weeks. Each data point represents one individual mouse [**(B)** *n* ≥ 4, two independent experiments, **(C)** *n* ≥ 5, two independent experiments, **(C)** (lower panel): *n* = 3, one experiment **(J)** *n* ≥ 3, two independent experiments. Statistical testing was performed with multiple paired *t*-test using Prism v9 (GraphPad). *p*-values are shown as *<0.05, **<0.01, and ***<0.005 where each statistical significance was found.

γδ T cells are thought to differentiate from progenitors located in the thymic DN compartment, which include DN1, DN2, DN3, and DN4 subsets (33). We set out to determine whether DN cells from GFI1 KO mice may have gained the ability to preferentially produce Vγ6^+^ γδ T cells. To test this, we cultured total DN cells from adult WT and GFI1 KO thymi on OP9-DL1 cells (**Supplementary Figure 6A**) by excluding γδ T cells, NK cells, myeloid cells, B cells, and conventional T cells (**Supplementary Figures 6B, C**). Under these conditions, we observed that GFI1 KO DN cells can give rise to higher proportions of the CD24^-^CD44^+^CD45RB^-^ γδ T cells (known as γδT17) in the CD45^+^ cells compared to WT DN cells (**Figures 6A, B**). These data suggested that the bias toward the γδT17 subset in the absence of GFI1 has its origin in early precursors of γδ T cell differentiation, which are not yet fully committed to the γδ T cell lineage.

It is already known that most γδ T cells differentiate from thymic DN2 or DN3 cells (40, 44), but recently the DN1d and DN1e subsets have also been shown to generate γδ T cells (44, 45). The DN2 and DN3 subsets are strongly decreased in GFI1 KO mice compared to WT animals, and the DN1a/b subpopulations are almost absent (**Figure 6C**). Even though DN1d and DN1e subsets were significantly decreased in numbers in GFI1 KO mice, their frequencies were increased in *Gfi1*-deficient mice compared to WT or cre-expressing GFI1 WT animals (**Supplementary Figure 6D**).

To gain more insight into the origin of the expanded γδT17 cell population in GFI1 KO mice, we performed a scRNA-seq analysis of pools of DN1, DN2, and DN3 cells from adult GFI1 WT and KO thymus. Our analysis showed that these cells fall into 10 distinct clusters (**Figure 6D**; **Supplementary Figures 6E,F**), which could be annotated as DN1, DN2, and DN3 cells and their subtypes (45, 63, 67) (**Figure 6E**; **Supplementary Figure 6E**). Interestingly, clusters 6 and 8 contained populations of the DN1d and DN1e subsets that were identified as *Rorc^+^*DN1d and *Rorc^+^*DN1e, expressing genes associated to the γδT17 subtype such as *Maf*, *Rorc*, *Blk*, *Cxcr6*, and *Scart1* and were largely found in adult GFI1 KO thymus (**Figures 6D–H**). Another cluster (cluster 5) identified as *Il2rb^+^*DN1e was also overrepresented in GFI1 KO thymus compared to WT thymus (**Figures 6D–G**). These data show that DN1d and DN1e cells were still present in adult GFI1 KO mice, with *Rorc*^+^DN1e cells being the most expanded population in GFI1 KO DN population (**Figure 6G**). This correlated with the high *Gfi1* expression levels found in DN1e cells compared to other DN1 cells (**Supplementary Figure 6G**).

We also observed that cluster 0 is markedly enriched in GFI1-deficient cells (**Figures 6D–F**), but inspection of the integrated UMAP together with differential expression analysis suggested that clusters 0 and 2 represent transcriptionally distinct DN3a states that are strongly associated with genotype. Whereas cluster 2 is predominantly composed of WT cells, cluster 0 is largely populated by GFI1-deficient cells (**Figures 6D–F**). Consistent with this interpretation, differential expression analysis of DN3a, DN3a→DN3b, and DN3b populations revealed substantial transcriptional changes upon GFI1 deletion (**Supplementary Figure 6H**), indicating that loss of GFI1 alters the transcriptional state of DN3 thymocytes, resulting in their segregation during unsupervised clustering.

The most upregulated genes in the pool of DN1–DN3 cells from adult GFI1 KO thymus compared to WT control DN1–DN3 cells contained genes related to Vγ6^+^ γδT17 cells, such as *Maf* and *Scart1* (**Figure 6I**), confirming that a γδT17 program was already active in DN1 cells from GFI1 KO mice. We confirmed, by flow cytometry, the higher proportions of c-MAF^+^ cells in GFI1 KO and in *vav-cre Gfi1^fl/fl^* DN1e, DN2, and DN3 cells (**Supplementary Figure 6I**) and identified an expanded c-MAF^+^RORγt^+^ DN1e population in GFI1 KO and in *vav-cre Gfi1^fl/fl^* thymus as compared to cre-expressing GFI1 WT controls (**Figures 6J, K**). This c-MAF^+^RORγt^+^ DN1e population overlapped with the abnormal DN1 population that we had described previously (17) (**Figure 6K**), which expressed a high level of CD44 and a low level of CD25. This suggested that GFI1 restricts or controls a DN1e population bearing the potential to give rise to Vγ6^+^ γδT17 cells.

### Characterization of the DN1 MAF^+^/RORγt^+^ and DN3 compartments in GFI1 KO mice

**Figure 7:**
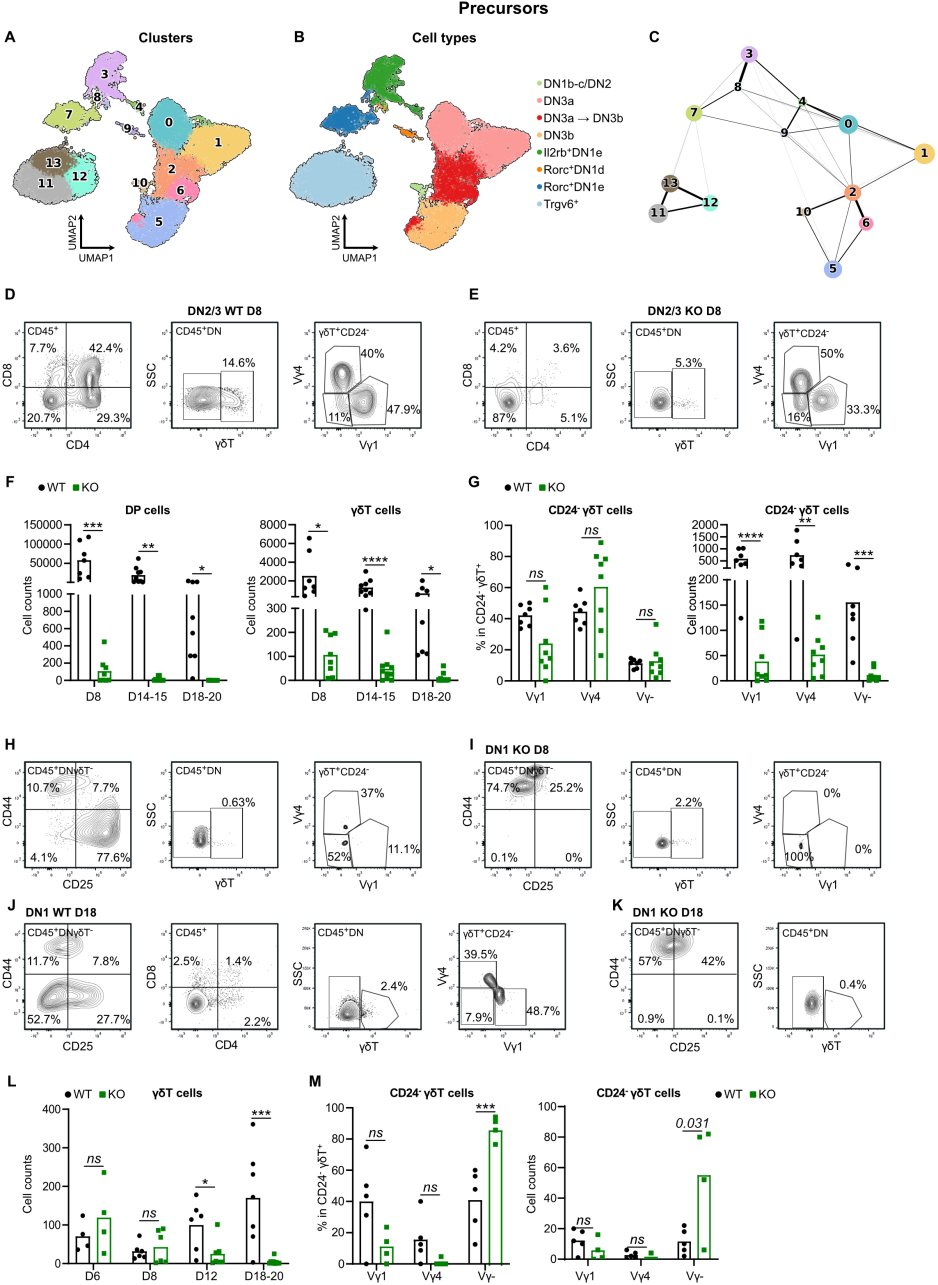
Potential of differentiation of precursors in thymus of adult mice. **(A, B)** UMAP representation based on gene expression profiling by sc-RNA-seq of thymic DN1, DN2, DN3, and γδ T cells from WT and GFI1 KO mice colored by cluster **(A)** and cell type **(B)**. **(C)** Partition- based Graph Abstraction (PAGA) graph after running PAGA on thymic DN1–3 pre-T cells and *Trgv6^+^* thymic γδ T cells from WT and KO at cluster resolution. **(D, E)** Representative plots showing the DP (CD45^+^CD4^+^CD8^+^) population, the γδ T cells (CD45^+^CD4^-^CD8^-^γδT^+^), and the CD24^-^γδ T subsets according to the expression of Vγ chains after gating on live singlets CD45^+^CD4^-^CD8^-^γδT^+^CD24^-^ cells from sorted WT **(D)** and GFI1 KO **(E)** DN2/3 cells cultured on OP9-DL4 as described in **Supplementary Figure 7I**. **(F)** Cell counts of generated DP cells (CD45^+^CD4^+^CD8^+^) and γδ T cells (CD45^+^CD4^-^CD8^-^γδT^+^) from WT and GFI1 KO DN2–3 cultured on OP9-DL4 as described in **Supplementary Figure 7I** at different time points of the coculture. **(G)** Cell counts and frequencies of the indicated subsets based on the expression of Vγ chains of mature CD24^-^ γδ T cells after coculture of sorted DN2–3 cells from WT or GFI1 KO mice on OP9-DL4 cells as described in **Supplementary Figure 7I**. **(H–K)** Representative plots showing the DN subsets based on CD44 and CD25 expression after gating on CD45^+^CD4^-^CD8^-^γδT^-^ cells, DP (CD45^+^CD4^+^CD8^+^) population, the γδ T cells (CD45^+^CD4^-^CD8^-^γδT^+^), and the CD24^-^γδ T subsets according to the expression of Vγ chains after gating on live singlets CD45^+^CD4^-^CD8^-^γδT^+^CD24^-^ cells from sorted WT **(H, J)** and GFI1 KO **(I, K)** DN1 cells cultured on OP9-DL4 as described in **Supplementary Figure 7** at different time points D8 **(H, I)** and D18 **(J, K)**. **(L)** γδ T cell counts generated from WT or GFI1 KO DN1 cultured on OP9-DL4 as described in **Supplementary Figure 7** at different time points. **(M)** Cell counts and frequencies of the indicated subsets of mature CD24^-^ γδ T cells after coculture of sorted DN1 cells from WT or GFI1 KO mice on OP9-DL4 cells as described in **Supplementary Figure 7I**. Each data point represents one individual mouse [**(F, G)** *n* ≥ 7, three independent experiments; **(L, M)** *n* = 4, two independent experiments]. Adult mice meaning mice older than 5 weeks. Statistical testing was performed with paired *t*-test and multiple paired *t*- test using Prism v9 (GraphPad). *p*-values are shown as *<0.05, **<0.01, ***<0.005, and ****<0.001 where each statistical significance was found.

To determine if this DN1e subpopulation that was expanded in GFI1 KO mice can preferentially differentiate into Vγ6^+^ γδT17 cells, we performed a trajectory inference analysis using partition-based graph abstraction (PAGA) by combining the scRNA-seq data obtained with DN1–3 cells and the *Trgv6^+^* cluster from WT and GFI1 KO thymic γδ T cells (**Figures 7A–C**). PAGA can infer developmental trajectories from scRNA-seq data by calculating the probability of transition between cells (68). This provides indications about relationships and connections of cells and their precursors. Using this method, a potential trajectory was obtained from cluster 7 containing *Rorc*^+^DN1e, almost exclusively found in GFI1 KO thymus, to the three clusters containing the Vγ6^+^γδT17 cells identified as *Trgv6^+^* (clusters 11, 12, and 13) that represented almost the totality of γδ T cells in GFI1 KO thymus. This indicated that the *Rorc*^+^DN1e subset enriched in GFI1 KO mice is developmentally linked to the γδT17 cells expressing the Vγ6 chain.

The cluster containing DN1–DN2 cells (cluster 10) was connected to the different DN3a and/or DN3b clusters (clusters 2 and 5) but was not linked to the *Trgv6^+^* clusters (clusters 11, 12, and 13) containing Vγ6^+^γδT17 cells (**Figures 7A–C**), reinforcing the idea that the expanded Vγ6^+^ γδT17 population in GFI1 KO thymus does not originate from the DN2 or DN3 compartment but most likely from the DN1e subset. The analysis of *Cd2-cre*, Gfi1^flox/flox^ mice, where a deletion of *Gfi1* occurs after the DN1 stage in the DN2–3 precursors during T cell development, did not show changes in numbers for γδ T cells negative for Vγ1 and Vγ4 chain expression, expressing RORγt^+^ or producing IL-17A compared to cre-expressing GFI1 WT control mice (**Supplementary Figures 7A–H**). One of the differences was the decreased numbers of Vγ1^+^ γδ T cells in the thymus and in the spleen of the *Cd2-cre* Gfi1^flox/flox^ mice compared to cre-expressing GFI1 WT control mice, meaning that GFI1 could have an impact on the production of this particular γδ T cell subset when deleted at the DN2/3 stage (**Supplementary Figures 7B–D**). We also observed a slightly increased proportion of RORγt^+^ cells in the spleen and a slightly increased frequency of IL-17A^+^ cells in the thymus, but nothing comparable to what was found in the total GFI1 KO mice or in the *vav-cre Gfi1^fl/fl^* mice. This strongly suggests that the DN2/3 T cell precursors are not the source of the accumulated Vγ6^+^ γδ T cell population seen in mice lacking GFI1.

This was also confirmed when we cultured DN2/3 precursors on OP9-DL4 cells in the presence of IL7 and FLT3 (**Supplementary Figures 7I, J**). After several days of coculture, the WT DN2/DN3 cells differentiated as expected mostly into DP cells, while GFI1 KO DN2/DN3 cells were still mostly found in the DN compartment and did not differentiate into DP cells (**Figures 7D–F**). Both WT and GFI1 KO DN2/DN3 cells were able to produce γδ T cells, but DN2/DN3 from GFI1 KO thymus produced a smaller amount of γδ T cells compared to WT DN2–3 cells (**Figures 7D–F**), reducing the number of the CD24^-^ subtypes (**Figure 7G**). The γδ T cells differentiated from DN2/3 lacking GFI1 did not show a preference to generate higher proportions of CD24^-^Vγ1^-^Vγ4^-^ γδ T cells over the other subtypes but produced all γδ T cell populations in similar proportions to WT DN2–3 cells (**Figures 7D, E, G**). We further tested whether a developmental link exists between GFI1 KO DN1 cells and the Vγ6^+^ γδ T cells by also culturing sorted DN1 cells from WT or GFI1 KO mice on OP9-DL4 cells in the presence of IL7 and FLT3 (**Supplementary Figures 7I, J**). As expected, WT DN1 cells were able to progress though the different stages of T cell differentiation, while GFI1 KO DN1 remained mostly in the DN1 stage after 8 or 18 days of culture (**Figures 7H–K**). Only very few cells developed into γδ T cells from WT DN1 and GFI1 KO DN1 cells, with a peak of γδ T cell production after 12 days for WT precursors, while GFI1 KO DN1 cells produce most of the γδ T cells earlier in the coculture before being lost (**Figure 7L**). We gated the cells surviving in the coculture on CD24^-^γδ T cells and found that GFI1 KO did indeed produce higher proportions of Vγ1^-^Vγ4^-^ γδ T cells (most likely meaning Vγ6^+^) compared to WT DN1 cells (**Figure 7M**). However, the number of cells produced was very low (**Figure 7M**), suggesting that GFI1 KO DN1 cells most likely require other signals to either generate the Vγ6^+^ γδ T cells or to fully support this biased γδ T cell population, leading to their accumulation in adult Gfi1-deficient mice. Our data indicate that the expanded γδT17 cell population in GFI1 KO mice are unlikely to originate from DN2–DN3 cells. DN1 subsets, particularly DN1e, may instead contribute to this bias. However, this interpretation remains tentative, as additional experiments are required to confirm the developmental origin of these cells.

### GFI1 can directly regulate *Maf*

**Figure 8:**
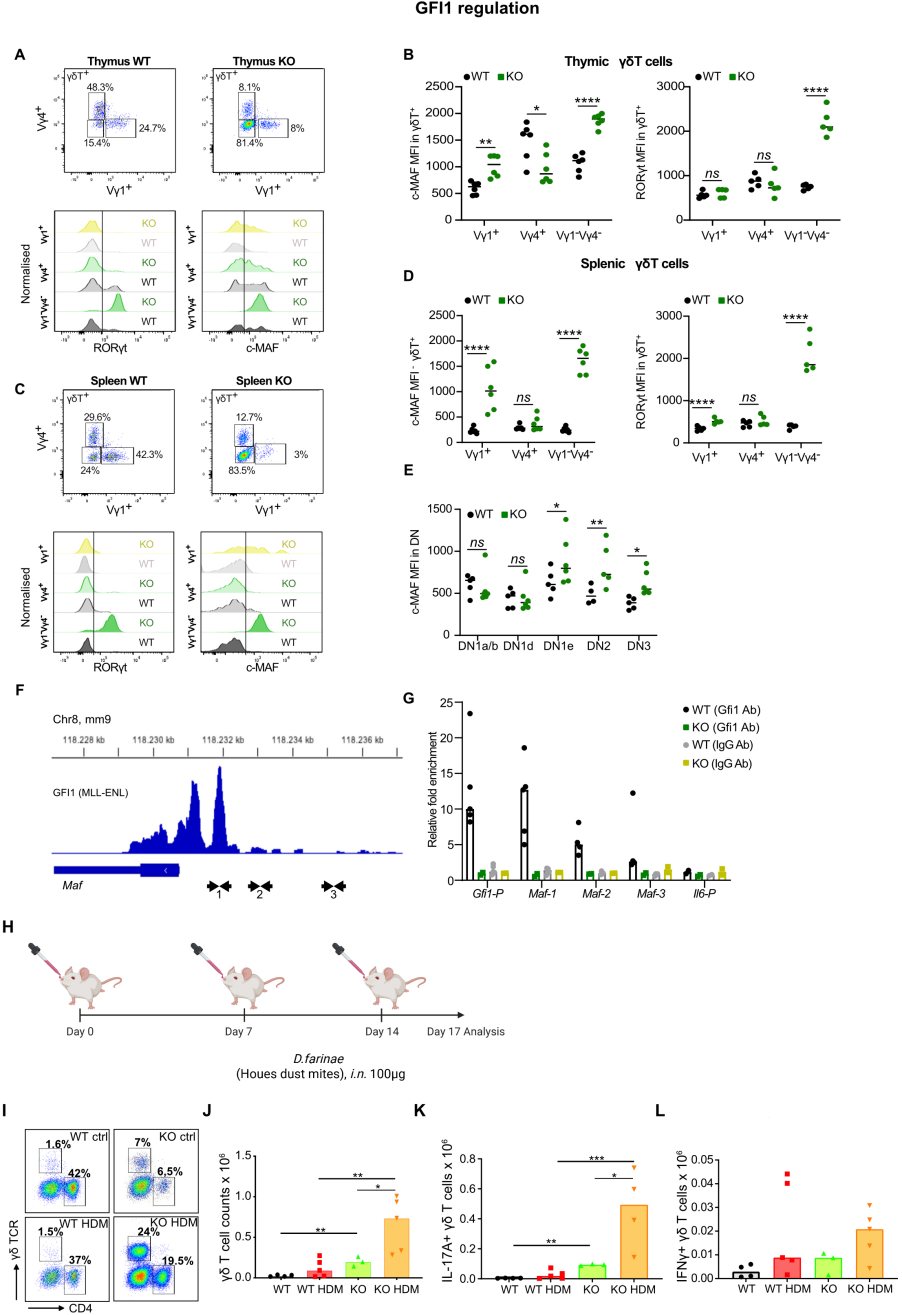
GFI1 regulation and function in an allergic response in adult mice. **(A)** RORγ and c-MAF MFI in the indicated subsets of thymic γδ T cells based on Vγ1 and Vγ4 expression from WT and GFI1 KO mice. **(B)** c-MAF and RORγt MFI quantification for the indicated thymic γδ T cell subsets comparing WT and GFI1 KO mice. **(C)** RORγ and c-MAF MFI in the indicated subsets of splenic γδ T cells based on Vγ1 and Vγ4 expression from WT and GFI1 KO mice. **(D)** c-MAF and RORγt MFI quantification for the indicated splenic γδ T cell subsets comparing WT and GFI1 KO mice. **(E)** c-MAF MFI quantification comparing WT and GFI1 KO mice for the indicated thymic DN progenitors. **(F)** Peaks for GFI1 on the *Maf* promoter region from the MLL-ENL ChIP-Seq (GSE31657). **(G)** ChIP-PCR on different potential binding sites (arrowheads) on *Maf* promoter for GFI1 identified in **(F)** with WT and GFI1 KO DN cells. *Gfi1* promoter (*Gfi1-P*) was used as positive control, and *Il6 promoter* region was used as negative control. **(H)** Expansion of γδ T cells in GFI1 KO lungs after intranasal instillation of house dust mite (HDM) allergen extracts (Sigma). Experimental setup and timeline of the HDM treatment. **(I)** FACS plots of CD45^+^CD3^+^ cells from lungs before and after HDM for γδ TCR and CD4 in WT and GFI1 KO mice. **(J)** γδ T cell counts in lungs of untreated or HDM-treated WT and GFI1 KO mice. **(K)** Cell counts of IL-17A^+^ and **(L)** IFN-γ+ γδ T cells before and after HDM treatment in WT and GFI1 KO lungs. Each data point represents one individual mouse [**(B, D, E)** *n* ≥ 5, two to three independent experiments, **(G)** *n* = 5, two independent experiments, **(J-L)**: *n* ≥ 3, two independent experiments). Statistical testing was performed with multiple paired *t*-test using Prism v9 (GraphPad). *p*-values are shown as *<0.05, **<0.01, ***<0.005, and ****<0.001 where each statistical significance was found.

The scRNA-seq analysis indicated that the expression of *Maf* was increased in clusters from GFI1 KO cells, and flow cytometry data confirmed this for the c-MAF protein, known to be a regulator of γδT17 cell differentiation and maintenance (69). We also confirmed the overexpression of MAF in GFI1 KO Vγ1^-^Vγ4^-^ thymic γδ T cells and also in Vγ1^+^ thymic γδ T cells compared to WT control cells (**Figures 8A, B**). However, this upregulation of MAF was only associated with a higher expression of RORγt in the Vγ1^-^Vγ4^-^ thymic γδ T cells (**Figure 8D**). We observed similar data in splenic γδ T cells, with an upregulation of c-MAF in GFI1 KO Vγ1^-^Vγ4^-^ and Vγ1^+^ splenic γδ T cells, associated with a strong RORγt upregulation only in Vγ1^-^Vγ4^-^ splenic γδ T cells (**Figures 8C, D**). We also found that total DN3 cells from GFI1 KO mice that have a very different transcriptome profile compared to WT DN3 cells showed upregulation of *Maf* (**Supplementary Figure 6I**), which was also confirmed by flow cytometry for c-MAF expression in DN2 and DN3 cells (**Figure 8E**).

In addition, we also found a higher expression of c-MAF specifically in the GFI1 KO DN1e cells compared to WT cells (**Figure 8E**), but not in DN1d or DN1a/b cells. This correlated with the high expression of GFI1 shown in DN1e cells (**Supplementary Figure 6G**). These data suggested that *Maf* is a potential direct GFI1 target. To test this hypothesis, we interrogated available ChIP-Seq data from MLL-ENL lymphoblastic cells and observed several peaks with a GFI1 binding motif binding in the *Maf* 5′ region and peak for GFI1 (**Figure 8F**). Binding of GFI1 on *Maf* promoter was also found in other available ChIP-Seq data with different cell types (**Supplementary Figure 8B**). A query of the JASPAR database confirmed the location of several GFI1 binding motifs of the *Maf* gene from the transcription start site up to several kilobases upstream (**Figure 8F**). ChIP-qPCR with purified WT and GFI1 KO DN cells and primers covering these putative binding sites indicated binding of GFI1 located in regions where the primer pairs were designed (**Figure 8G**). As controls, we used GFI1 KO DN cells, where no enrichment was found in this region, and the *IL-6* promoter, where GFI1 does not bind, confirming occupation of the Maf promoter by GFI1 in WT DN pre-T cells (**Figure 8G**). This suggested that GFI1 directly represses the expression of *Maf* and, with this, the expansion of Vγ6^+^ γδ T cells.

### GFI1 restricts γδT17 cells in an inflammatory allergy reaction

Next, we tested whether GFI1 deficiency is also associated with an increase of γδT17 cells in the context of an infectious agent that elicits a Th2 response. We used a house dust mite (*D. farinae*, HDM)-induced allergic airway model (70) which provides a relevant setting to test whether GFI1 KO mice exhibit altered γδ T cell responses despite their known defect in classical Th2 immunity (25). House dust mite exposure drives a strong type 2 inflammatory environment but also engages γδT17 cells. After three intranasal applications of a HDM preparation at 100 µg each at 7-day intervals, we observed a strong expansion of γδ T cells that express IL-17 in the lung of GFI1 KO mice compared to HDM-treated WT animals (**Figures 8H–K**). An expansion of IFN-γ-expressing γδ T cells was not observed (**Figure 8L**). This suggested that GFI1 controls the expansion of γδT17, but not γδT1 cells in a Th2 immune response.

## Discussion

In this study, we report the expression of GFI1 in γδ T cells and their precursors and present evidence for a novel regulatory role of this transcription factor in restricting γδ T cell fate and cellularity across various tissues, including thymus, spleen, lung, and lymph nodes. Our findings demonstrate that genetic ablation of GFI1 results in a substantial expansion of RORγt^+^Vγ6^+^γδ T cells producing IL-17 and a concurrent decrease in Vγ1^+^ and Vγ4^+^ γδ T cells. This bias toward the γδT17 subtype emerges postnatally in GFI1 KO mice and persists in the adult thymus, where the Vγ6^+^ subset that secretes IL-17 becomes the predominant population among γδ T cells. GFI1 also regulates the transcriptional program of Vγ6 T cells in addition to controlling their abundance.

Furthermore, GFI1 KO mice exhibit an increased number of c-MAF^+^/RORγt^+^ cells in the DN1e subset, which has been described as containing precursors for γδT17 cells (63). *Gfi1*-deficient DN thymocytes and γδ T cells exhibit an upregulation of the bZip transcription factor c-MAF, which is known to regulate γδT17 fate (69). We further demonstrate that GFI1 occupies the promoter region of the *Maf* gene at a cognate GFI1 binding site, suggesting that GFI1 controls the cellularity of γδ T cells and their progenitors through the potential regulation of a c-MAF-dependent transcriptional network.

The specific expansion of only Vγ6^+^ γδ T cells without affecting the fate of other γδT subtypes (Vγ4^+^), which could also adopt a γδT17 profile (33, 41, 64), supports the idea of an intrinsic effect of GFI1 in γδ T cell development. In addition, the experiments with *vav-cre Gfi1^fl/fl^* mice, which delete *Gfi1* only in hematopoietic cells, also ruled out the possibility that the non-hematopoietic environment, such as epithelial cells, is causing the specific expansion of Vγ6^+^ γδT17 cells seen in these animals. Transplantation experiments of WT cells into GFI1 KO animals did not reproduce the strong bias toward the γδT17 subtype observed in GFI1 KO mice, even though we observed a slight increase of both IL-17A and RORγt frequencies in donor WT γδ T cells in the spleen of the GFI1 KO recipients. This effect could be due to the transplantation itself or may suggest that the environment of the GFI1 KO spleen could have a slight impact on the fate of Vγ4^+^ γδ T, which is the other γδ T subset capable of becoming γδT17 cells.

However, it remained unclear which cell population is responsible for the expansion of Vγ6^+^ γδT17 in GFI1 KO mice. We showed that Vγ6^+^ γδT17 cells start to increase in number in GFI1 KO mice only after birth. It is thus possible that *Gfi1-*deficient precursors for Vγ6^+^ γδT17 cells from the fetal thymus continue to produce these cells when they reach adult tissues, whereas their WT counterparts cease their production. Transplantation of adult BM cells from GFI1 KO mice did not recapitulate the expansion of Vγ6^+^ γδT17 cells in recipient animals (data not shown), which rules out the presence of a γδ T precursor within the adult thymic DN pre-T cell population. It remains unclear, therefore, which cells give rise to c-MAF^+^/RORγt^+^ cells in the DN1e subset in the adult thymus where they are under the restriction of GFI1 in WT mice. These cells were detected in flow cytometry analyses in WT mice, albeit at a very low number. It is therefore conceivable that these cells expand into Vγ6^+^ γδT17 cells in the thymus when GFI1 is absent. Both scRNA-seq and flow cytometry show this expanded population of c-MAF^+^/RORγt^+^ cells in the DN1e pre-T cells from GFI1 KO mice very clearly. The transcription pattern of this DN1e subpopulation in GFI1 KO mice supports this notion and suggests that a DN1e subset exists with a specific transcriptional signature which is maintained after birth in the adult thymus that is associated with Vγ6^+^ γδ T cells producing IL-17. Although these data point to a fetal origin of the expanded Vγ6^+^ γδT17 cell population in GFI1 KO mice, further experimentation with fetal and adult pre-T cells are necessary to firmly support such a conclusion. However, it is also possible that signals supporting the Vγ6 bias and the subsequent expansion of Vγ6^+^ γδT17 cells only emerge in adulthood. Such signals may arise postnatally, for example, during weaning, or through the establishment of an environment shaped by prolonged exposure to external antigens and conditions *ex utero*.

The decrease of Vγ1^+^ and Vγ4^+^ γδ T cells that we observed in GFI1 KO mice, alongside the expansion of Vγ6^+^ γδT17, could be a consequence of the decreased cell number in the DN3 subset. Lower numbers of Vγ1^+^ and Vγ4^+^ γδ T cells, were observed in fetal and adult thymus of GFI1 KO mice as compared to WT controls, in contrast to Vγ6^+^ γδ T cells, which expanded only after birth. It is thus likely that separate precursors exist for the Vγ1^+^ and Vγ4^+^ γδ T cells and the Vγ6^+^ subset. In addition, DN3 cells lacking GFI1 have a distinct transcriptomic profile from their WT counterparts, which could also affect the production of Vγ1^+^ and Vγ4^+^ γδ T cells since they develop from this thymic DN population. Surprisingly, an upregulation of MAF was also observed in the thymic and splenic Vγ1^+^ γδ T cells but not in the Vγ4^+^ γδ T cells, the other population where MAF can be expressed. We did not observe a differential expression of GFI1 (data not shown) between the different γδ T cell populations which could have explained this difference in MAF regulation. Another open question is why *Gfi1*-deficient Vγ6^+^ γδ T cells only expand in the periphery. It is possible that the environment in the thymus allows the initial bias toward a Vγ6^+^ γδ T cell fate in the GFI1 KO but restricts their expansion. Once the cells egress to the periphery, this restriction may be lifted, allowing the cells to expand. More experiments are required to reveal the additional regulatory or compensatory pathways that underlie this phenotype and that regulate MAF expression in different γδ T cell subtypes in addition to GFI1.

A DN1-like (CD117^−^CD24^−^CD25^−^CD44^+^) precursor subpopulation (DN1e) was more recently identified to be programmed or pre-committed to become Tγδ17 cells independently of TCR signaling (44, 45). These cells express *Rorc*, *Sox4*, *Tcf7*, *Tcf12*, *Maf*, *Il7r*, *Scart2*, and *Blk* and are predisposed to become CCR6^+^ IL-17A-producing cells (45). c-MAF promotes the expression of genes essential for Tγδ17 cell identity, such as *Rorγt* and *Blk*, which are key players in the Tγδ17 program and whose expression is also found to be increased in GFI1 KO γδ T cells (69, 71). Our data suggest that lineage-committed precursors and γδT17 cells are controlled not only by TCF1 and HEB/SOX13 (72, 73), which regulate c-MAF, but also by GFI1, which acts upstream of c-MAF. This would be consistent with the model that lineage fate can be prewired cell-intrinsically and is not necessarily specified by antigen receptor signals (45). This also lends further support to the view that the MAF^+^/RORγt^+^ cell population that we found expanded in thymic DN1e cells from GFI1 KO mice represents the precursor for the expanded IL-17-producing Vγ6^+^ γδ T cells. However, further experimentations would be needed to support this hypothesis because the coculture of DN1 cells from GFI1 KO mice on OP9-DL4 layer or in FTOC (data not shown) was not robust enough to support the expansion of Vγ6^+^ γδ T, even though a bias was observed toward this subtype. It may suggest that other signals in the periphery, such as the cytokine environment specifically found in GFI1 KO mice, are required to support the intrinsically biased γδ T populations to expand in *Gfi1*-deficient mice.

It has previously been shown that c-MAF is highly expressed in RORγt^+^ γδ T cells, which are γδT17 cells, but not in other γδ T cell subsets such as γδT1 cells (69). c-MAF functions by promoting the expression of key genes in the type 17 program (e.g., *Rorc*, *IL-17a*, *Blk*) and possibly also *IL-23R* and antagonizes negative regulators like TCF1, resulting in the upregulation of *Ifng*. c-MAF helps γδ T cells adapt to specific tissue microenvironments by regulating metabolic pathways and integrates signals from Il1-β, IL-23, and interactions with epithelial cells to fine-tune γδ T cell responses (74). These reports identified c-MAF as a universal and essential regulator of γδT17 cell differentiation and function but still lacked insights into the molecular mechanisms that control c-MAF expression.

Our results using ChIP-qPCR and ChIP-seq data from the public domain showed that GFI1 occupies the *Maf* promoter region. This observation, together with our finding that c-MAF expression is upregulated in all tested *Gfi1-*deficient γδ T cells, indicates that GFI1 acts as an upstream negative regulator of c-MAF. It is therefore conceivable that the direct transcriptional repression of the *Maf* gene by GFI1 is necessary to maintain and restrict the development of Vγ6 γδT17 cells and that the absence of GFI1 leads to the developmental defect in these γδ T cells seen in GFI1 KO animals. The upregulation of c-MAF and c-MAF target genes such as *IL-17* and *Blk* supports such a role of GFI1, but further studies are needed to establish a direct mechanistic link.

It is also known that GFI1 antagonizes RORγt expression through GATA3 (3, 23, 71) and that c-MAF induces the expression of RORγt and synergizes with RORγt to regulate IL-17-producing cells. It is thus conceivable that a GFI1/c-MAF/RORγt regulatory circuit exists, which controls the expression of IL-17A and ensures the cellularity and functionality of γδT17 cells. GFI1 has been shown to regulate Notch/IL7 signaling (18) which is important for γδ T cells, suggesting that it might also be involved at different levels such as γδ T cell development. Our data do not exclude contribution from these pathways, and further experiments would be required to define the link between Notch/IL7 signaling and GFI1 in γδ T cells.

The ability of c-MAF to fine-tune IL-17 production has been highlighted as a potential therapeutic target for controlling γδ T-cell-mediated inflammation without compromising their protective roles (75). Given our findings that GFI1 KO mice react with a strong expansion of IL-17 producing γδ T cells against house dust mites, this may become relevant for allergen-induced immune responses and inflammatory reactions. Our finding that IFN-γ^+^ γδ T cells were not expanded in HDM-challenged GFI1 KO mice confirmed that the bias toward IL-17^+^Vγ6 γδ T cells under the conditions of GFI1 deficiency is maintained and even increased in disease model. Strategies to modulate c-MAF activity or its interaction with RORγt are being explored as therapeutic options to balance inflammation and immunity. Our findings which place GFI1 upstream of c-MAF may point to new possibilities to modulate this regulatory circuit since GFI1 is in a complex with LSD1 for which small molecule inhibitors exist (76).

## Materials and methods

### Mice

The generation of GFI1 KO, GFI1-EGFP, and GFI1-fl/fl mice was previously described (13, 28, 59). C57BL6/J CD45.2 congenic mice (B6.SJL-*Ptprc^a^ Pepc^b^*/BoyJ) and *Vav-cre* mice (B6.Cg-Tg(VAV1-cre)1Graf/MdfJ) were obtained from Jackson labs (Bar Harbour, MN, USA). C57BL6/J CD45.1/2 mice were a gift from Dr. Javier Di Noia, IRCM, Montreal, Canada, and hCD2-iCre mice (B6.Cg-Tg(CD2-icre)4Kio/J) were a gift from Dr. André Veillette, IRCM, Montreal, Canada. All mice were maintained on the C57BL6/J background. The mice were housed and bred in the IRCM specific-pathogen-free facility in a 12-h dark/light cycle with food and drink *ad libitum*. All mouse work was reviewed and approved by the animal protection committee at the IRCM (protocols #2020-09), according to the guidelines of the Canadian Council of Animal Care.

### Tissue dissociation

Lungs from adult mice (at least 5 weeks old) were perfused with 10 mL of PBS *via* the right ventricle and then were cut into small pieces with scissors and a scalpel. Lung pieces were dissociated and digested in a buffer with collagenase IV (1 mg/mL) (Gibco) and DNase I (0.2 mg/mL) (Sigma) in RPMI medium for 30 min at 37 °C. The cells were aspirated several times with a syringe and needle (18 gauge) every 10 min to further the dissociation and digestion process. Lung cell suspensions were filtered and washed in PBS. Thymi, spleen, and cervical and inguinal lymph nodes from adult mice (at least 5 weeks old) were mechanically dissociated to obtain a cell suspension which was filtered. Then, red blood cell lysis buffer (Sigma) was added to the lung, thymic, splenic, or LN cell pellet for 4 min and washed before staining or sorting.

### Bone marrow chimera

Bone marrow (BM) cells from CD45.1/2 mice were obtained by flushing femurs and tibiae with cold PBS using a syringe with 23G needle. The red blood cells were subsequently lysed, and the cells were washed in PBS before being injected into the tail vein in 100 µL of PBS containing 2 × 10^6^ BM cells into either CD45.2 WT or GFI1 KO recipient mice. At 4 or 5 weeks after the transplantation, the recipient mice were sacrificed, and splenic, thymic, and lung cells were analyzed by flow cytometry.

### OP9-DL1 and OP9-DL4 culture

The OP9-DL1 and OP9-DL4 cells were a gift from the lab of Dr. Zúñiga-Pflücker (77). The OP9-DL1 cells were plated in 96-well flat-bottom culture plate at a density of 3,000 cells in 100 uL of OP9 medium (AMEM, 20% FBS, 1% Pen/strep, 2.2 g/L sodium bicarbonate, 50 μM β-mercaptoethanol). The day after, the same number of WT and GFI1 KO sorted DN1, DN2–DN3, or total DN cells were co-cultured on OP9-DL1 or OP9-DL4 monolayers in DMEM-glutamax medium containing 10% of FBS, 1% Pen/strep, 50 μM β-mercaptoethanol, and 10 mM Hepes with Flt3 (5 ng/mL, Peprotech) and IL7 (1 ng/mL, Peprotech). Thymocytes were transferred to a new OP9-DL1 or OP9-DL4 monolayer with fresh cytokines every 3 days.

### Flow cytometry and cell sorting

For the analysis of cells from spleen, lung, lymph nodes, and thymus from mice by flow cytometry, a Fortessa (BD Biosciences) flow cytometer and FlowJo software were used. Cell sorting was performed on a FACSAria III Sorter (BD Biosciences). For IL-17A and IFN-γ intracellular staining, cells were cultured for at least 3 h with GolgiPlug (BD Biosciences), PMA (50 μM, Sigma), and ionomycin (1 mM, Sigma) and stained using the eBioscience cytokine intracellular staining kit (Invitrogen). For IL-22 staining (78), cells were first cultured with IL-23 (20 ng/mL, R&D Systems) overnight before stimulation with PMA (50 μM, Sigma) and ionomycin (1 mM, Sigma) in the presence of GolgiPlug (BD Biosciences) for the last 4 h. Intracellular staining for the transcription factors RORγt and c-MAF was also done using eBioscience FoxP3 intracellular kit (Invitrogen). For phosphoSTAT3 intracellular staining, after surface staining, the cells were stimulated for 15 min at 37 °C with either IL-23 (20 ng/mL, R&D Systems) or IL7 (10 ng/mL, Peprotech) and washed with cold PBS. The cells were subsequently fixed for 15 min at 37 °C with Biolegend fixation buffer (79) before being permeabilized with the Biolegend True-phos Perm buffer (425401) for 30 min at 4 °C. After washing, the cells were incubated with Phospho-STAT3 antibody for 30 min at 4 °C. For the splenic γδ T cell culture, sorted splenic γδ T cells were cultured *in vitro* in DMEM medium containing 10% FBS, 1% Pen/Strep, 10 mM Hepes, and 50 μM β-mercaptoethanol in the presence or not of IL-23 (20 ng/mL, R&D Systems) and Il1-β (10 ng/mL, Biolegend) for 16 h, and GolgiPlug (BD Biosciences) was added for the last 4 h before cytokine staining. All antibodies directly conjugated to a fluorochrome or to biotin were purchased from BioLegend, eBiosciences, or BD Biosciences (**Supplementary Table 4**). For antibodies against TCR γ variable chains, the Tonegawa nomenclature is used throughout. For Vγ6 staining, the culture supernatant of the anti-Vγ6 IgM secreting-17D1 hybridoma cells was directly added to each well for staining (50 μL for 30 min at 4°C), followed by incubation with a secondary PE-conjugated rat anti-mouse IgM antibody (Invitrogen; clone RM-7B4; 1:100, 30 min at 4°C).

### PCR

RNA extraction was performed using Tri-Reagent (Life Technologies) for cell numbers between 1 × 10^6^ and 3 × 10^6^ or using the RNeasy Micro kit from QIAGEN for a lower number of cells following the company’s instructions (RNeasy Micro kit; QIAGEN). RT-PCR was performed using Superscript II (Invitrogen). RNA was incubated with oligodeoxy thymidine (Invitrogen), EDTA (Invitrogen), and dNTP (Invitrogen) for 10 min at 65 °C, then DTT (Invitrogen), Superscript buffer 5× (Invitrogen), RNaseOUT buffer (Invitrogen), and the enzyme Superscript II (Invitrogen) were added, and the reaction was subjected to PCR cycles (step 1, 25 °C for 10 min; step 2, 42 °C for 60 min; step 3, 70 °C for 15 min) to generate cDNA for real-time quantitative PCR. Real-time quantitative PCR was performed in triplicates on the ViiA7 real-time PCR machine (Applied Biosystems) in SYBR Green Master Mix (Applied Biosystems) containing specific primers for mouse. The primers used are *Gfi1 Forward*: GTGCCATCAGAGGAGGTGAA, *Gfi1 Reverse*: TCTGATAACCTGCGGCCAAT; *Gfi1b Forward*: CAGTCCTACGCCCACCTTGG, *Gfi1b Reverse*: AGTGTCCGAGTGGATGAGCA, and *Gapdh Forward*: ACTGAGCAAGAGAGGCC, *Gapdh Reverse*: TATGGGGGTCTGGGATGGAA.

### Single-cell RNA sequencing

Single-cell RNA sequencing on lungs was performed using 10x Genomics platform with the Chromium Next GEM Single Cell 3′ Kit v3.1. Total lung cells from adult mice (between 5 and 8 weeks old) were sorted on live cells using Hoecsht to stain the dead cells. Then, the cells were loaded on a Chromium controller on a chip G to target the recovery of 10,000 cells per sample, and libraries were constructed following the manufacturer’s recommendations. Library size distribution was assessed on a 2100 bioanalyzer (Agilent Technologies) using a High Sensitivity DNA Kit, and libraries were quantified using qPCR. Equimolar libraries were sequenced in paired-end reads (PE 28-91) on a Novaseq 6000 system (Illumina), with an average depth of 25,000 reads per cell. The single-cell RNA sequencing on sorted γδ T cells (from the spleen or the thymus DN1–DN2 of adult mice) or on sorted DN1–DN3 cells was performed using 10x Genomics platform with the Chromium Next GEM Single Cell 3′ v3.1 Kit and the 3’ Feature barcode kit for cell surface protein. To accelerate the sorting step, splenic cells or thymic cells were preselected to remove B cells (CD19), myeloid cells (Gr1, CD11b, and CD11c), and NK cells (NK1.1 and CD49b) using biotinylated antibodies and Mojo magnet selection (Biolegend) before FACS staining. CD8^+^ cells were also partially removed from the thymus using Mojo Magnet selection (Biolegend). Then, WT or GFI1 KO cells were stained with TotalSeq-B oligo-conjugated antibodies (Totalseq-B0302 and Totalseq-B0303, Biolegend) to hashtag the samples in order to reduce costs and decrease technical variability during the incubation with the fluorochrome-coupled antibodies for FACS sorting. The sorted cells were loaded on a chromium controller on a chip G to target a recovery of 20,000 cells per sample (10,000 cells per condition WT or KO), and libraries were constructed following the manufacturer’s recommendations. Library size distribution was assessed on a 2100 bioanalyzer (Agilent Technologies) using a High Sensitivity DNA Kit, and libraries were quantified using qPCR. Equimolar libraries were sequenced in paired-end reads (PE 100) on a Novaseq X system (Illumina). An average depth of 25,000 or 5,000 reads per cell was targeted for gene expression or hashtag libraries, respectively. Duplicates were used for all the single-cell RNA sequencing experiments, which means two WT and two GFI1 KO samples for each cell type. **Figures 1A–C** present only the analysis of CD3^+^ cells, which represents a subset of the dataset.

### Single-cell RNA-seq analyses

scRNAseq data were analyzed using python (v3.12.4) and the package scanpy (v1.10.4). Cells with <500 reads, <200 genes, or >20% mitochondrial genes were removed as outliers. Importantly, ribosomal genes, predicted genes with *Gm-*identifier, and mitochondrial genes were excluded for subsequent analysis. The samples were demultiplexed using hashsolo (80). The datasets were normalized using the normalize_total function with a target sum of 10,000, followed by log1p transformation and scaling with a maximum value of 10. The top 2,000 highly variable features were calculated using the flavor “seurat_v3” and the raw counts. When integrating the thymic DN and γδ T cells, harmonypy v0.0.10 was used to correct for batch effects (81). Dimensionality reduction was performed with default settings, while the resolution for clustering using the Leiden algorithm was set to 0.5 for the DN dataset, 0.8 for the thymic γδ T cell and lung datasets, and 1 for the splenic γδ T cell dataset. To characterize the clusters, differential gene expression analysis was done using the rank_genes_groups function. Graph abstraction was performed using the function paga in scanpy with default parameters using clusters as partitions (82).

### Chromatin immunoprecipitation

Chromatin immunoprecipitation (Ch-IP) for GFI1 was performed on 10 × 10^6^ fresh cells. Briefly, cells were cross-linked with 1% formaldehyde for 10 min before quenching with 125 mM glycine for 5 min. The cells were initially lysed in cell lysis buffer (5 mM PIPES, 85 mM KCl, 0.5% Nonidet P-40, freshly added protease inhibitors mixture, and 0.8 mM PMSF) for 15 min on ice with occasional vortexing. After centrifugation at 4 °C at 4,000 rpm for 5 min, the pellet was resuspended in nuclei lysis buffer (50 mM Tris [pH 8], 10 mM EDTA, 0.1% SDS, freshly added protease inhibitor mixture, and 0.8 mM PMSF) and incubated on ice for 20 min with occasional vortexing. The lysed cells were sonicated using a Covaris E220 to generate 200–600-bp fragments. One volume of immunoprecipitation dilution buffer (300 mM NaCl and 2% Triton X-100) was added to sonicated nuclei lysates and was immune-precipitated with 5 μg of anti-Gfi1 (AF3540; R&D Systems). Beads were added to harvest protein–DNA complexes. After de-crosslinking, the eluate was digested with RNase A and proteinase K. The chromatin‐associated DNA was extracted using QIAquick PCR Purification Kit (Qiagen). The primers for the ChIP-PCR are the following: *Gfi1 Promoter Forward*: AATTCGGGAGCTGAAGGCAA, *Gfi1 Promoter Reverse*: AGAGGGGCACACAGTTTAGC; *Maf-1 Forward*: CTCTCGAACTCCGAGGAAAGC, *Maf-1 Reverse*: GAGCGGCAGCGATTAGCAT; *Maf-2 Forward*: TGGTCAATAATGACAAGCGCA, *Maf-2 Reverse*: ATACAAATCGGAACCCCGGC; *Maf-3 Forward*: TCAATCAGTGTAGAAAACTGAACC, *Maf-3 Reverse*: ACAAGTCCGGAAACCCAGAT; *Il6 Promoter Forward:* GGCGGAGCTATTGAGACTGT, *Il6 Promoter Reverse:* AAACCGGCAAGTGAGCAGAT and *Chr2 Forward*: TGGGCATATCCCTGGAGCTT, *Chr2 Reverse*: GGCCATCCCACAGTCACAAC.

### Statistical analysis

Multiple paired *t*-test or paired *t*-test was used to calculate *p*-values where indicated. A *p*-value ≤0.05 was considered statistically significant. Survival curves were analyzed by log-rank Mantel–Cox test using GraphPad Prism (GraphPad Software, La Jolla, CA, USA). The *p*-values are as follows: *p < 0.05, **p < 0.01, ***p < 0.005, and ****p < 0.001.

## Data Availability

The data presented in the study are deposited in the GEO repository, accession number GSE294462.

## Supplementary material

Supplementary Figure 1 Phenotype of γδ T cells in adult GFI1 KO mice. **(A)** Violin plots showing the number of reads and genes as well as the percentage of mitochondrial genes quantified per cell in the lung tissue in the scRNA-seq data. **(B)** Dot plot showing the key differentially expressed genes in each cluster shown in **Figure 1A**. Color represents the scaled mean expression of the gene in the respective cluster, and dot size represents the fraction of cells in the cluster expressing the gene. **(C)** Representative plots on small intestine (SI) and colon lamina propria cells showing γδ T cells and CD4 T cells after gating on live (according to FSC/SSC) singlets CD45^+^CD3^+^ cells. SI and colon lamina propria cells were isolated following published protocols (83). **(D, E)** CD4^+^ T cell counts in the spleen, LN, and lung of WT and GFI1 KO mice **(D)** or control and *vav-cre GFI1^flox/flox^* mice **(E)**. Each Data point represents one individual mouse [**(C)** n ≥ 10, three to four independent experiments, **(D)** n=4, two independent experiments]. Adult mice meaning mice older than 5 weeks. Statistical testing was performed with paired *t*-test using Prism v9 (GraphPad). *p*-Values are shown as * < 0.05, ** < 0.01, *** < 0.005, and **** < 0.001 where each statistical significance was found.

Supplementary Figure 2 Expansion of the RORγt^+^IL-17a^+^ γδT subset in adult GFI1 KO mice. **(A)** Representative plots showing the expression of CD27 on gated live singlets CD45^+^γδT^+^CD4^-^ cells from WT and GFI1 KO spleen, LN and lung. **(B)** Representative normalized histograms of RORγt^+^ cells in either singlets CD45^+^CD4^-^γδT^+^ or CD45^+^CD4^+^γδT^-^ splenic, LN or lung cells from control and *Vav-cre Gfi1^flox/flox^* mice. **(C)** Frequencies of RORγt^+^ cells within singlets CD45^+^CD4^-^ γδT^+^ cells from splenic, LN and lung compared to control and *vav-cre GFI1^flox/flox^* mice. **(D)** splenic, LN and lung CD45^+^CD4^-^γδT^+^RORγt^+^ cell counts in control and *vav-cre GFI1^flox/flox^* mice. **(E)** Frequencies of RORγt^+^ cells in splenic, LN and lung CD45^+^CD4^+^γδT^-^ T cells in WT and GFI1 KO mice. **(F)** Representative plots showing the expression of IL-22 and IL-17A in gated singlets CD45^+^CD4^-^γδT^+^ cells in the spleen, LN and lung from WT and GFI1 KO mice. Cells were stimulated with IL-23 (20ng/mL) overnight and treated with PMA, ionomycin and GolgiPlug for the last 4 hours before staining. **(G)** Percentages of IL-17A^+^IL-22^+^ cells in γδ T cells from the spleen, LN and lung of WT and GFI1 KO mice. **(H)** IL-17A^+^IL-22^+^γδT^+^ cell counts in the spleen, LN and lung of WT and GFI1 KO mice. Each Data point represents one individual mouse [**(C, D)** n=4, two independent experiments, **(E)** n=8, three independent experiments (lung, n=3, one experiment), **(G, H)** n ≥ 4, two independent experiments). Adult mice meaning mice older than 5 weeks. Statistical testing was performed with paired *t*-test using Prism v9 (GraphPad). *p*-Values are shown as * < 0.05, ** < 0.01, *** < 0.005 and **** < 0.001 where each statistical significance was found. H:

Supplementary Figure 3 Expansion of Vγ6^+^ γδT17 cells in the periphery of adult GFI1 KO mice. **(A, B)** MFI of GFP^+^ cells from the GFI1 reporter mouse showing *Gfi1* expression in the indicated γδ T cells subtypes. Each Data point represents one individual mouse [**(A, B)** n=3, one experiment). **(C)** Violin plots showing the number of reads and genes as well as the percentage of mitochondrial genes quantified per cell in the splenic γδ T cells in the scRNA-seq data. **(D)** Dot plot displaying the mean expression level of an expanded set of marker genes across the identified 15 clusters shown in **Figure 3A**. Dot size indicates the fraction of cells within a given cluster expressing the respective gene, whereas color intensity reflects the mean normalized expression level of that gene within the cluster. The plot does not represent differential expression between GFI1 KO and WT cells. **(E)** Dot plot displaying the mean expression level of an expanded set of marker genes in the 6 clusters (0, 1, 3, 4, 5 and 12) expressing genes associated with the Vγ6^+^ γδ T cell subtype. **(F)** Differential gene expression showing key up- or down- regulated genes in GFI1 KO splenic *Trgv1^+^* γδ T cells versus WT splenic γδ T cells. **(G)** Differential gene expression showing key up- or down- regulated genes in GFI1 KO splenic *Trgv4^+^* γδ T cells versus WT splenic γδ T cells.

Supplementary Figure 4 Expansion of Vγ6^+^ γδT17 cells in the thymus of adult GFI1 KO mice. **(A)** Cell numbers (left) and frequencies in CD45^+^ cells (right) of γδ T cells (CD4^-^γδT^+^) in the thymus of control and *vav-cre GFI1^flox/flox^* mice. **(B)** Representative normalized histogram of RORγt^+^ cells in either CD45^+^CD4^-^γδT^+^ or CD45^+^CD4^+^γδT^-^ total thymocytes from control and *vav-cre*, *Gfi1*^flox/flox^ mice. **(C)** Frequencies of RORγt^+^ cells in thymic CD45^+^CD4^-^γδT^+^ comparing control and *vav-cre*, *Gfi1*^flox/flox^ mice (left) and thymic CD45^+^CD4^-^γδT^+^RORγt^+^ cell counts in control and *vav-cre, Gfi1*^flox/flox^ mice (right). **(D)** Violin plots showing the number of reads and genes as well as the percentage of mitochondrial genes quantified per cell in the thymic γδ T cells in the scRNA-seq data. **(E)** Differential gene expression showing key up- or down- regulated genes in GFI1 KO thymic γδ T cells versus WT splenic γδ T cells. **(F)** Representative plots showing CD44 and CD45RB expression in gated thymic CD45^+^CD4^-^γδT^+^CD24^-^ cells from WT and GFI1KO mice. **(G)** Cell counts of thymic CD45^+^CD4^-^γδT^-^CD24^-^CD44^+^CD45RB^-^ (γδT17), CD45^+^CD4^-^γδT^-^CD24^-^CD44^+^CD45RB^+^ and CD45^+^CD4^-^γδT^-^CD24^-^CD44^-^CD45RB^+^ cells in WT and GFI1 KO mice. **(H)** Representative plots of CD24 expression in γδ T cells and Vγ chains expression in gated CD24^-^ γδ T cells from D8–10 and D18-D20 old WT and GFI1 KO mice. **(I)** Cell numbers of thymic CD24^-^ γδT^+^ cells in D8–10 and D18-D20 old WT and GFI1 KO mice. **(J, K)** Vγ chain frequencies **(J)** and Vγ chain cell counts **(K)** in thymic CD24^-^ γδ T cells from D8–10 and D18-D20 old WT and GFI1 KO mice. Vγ^-^ = Vγ1^-^Vγ4^-^Vγ5^-^. Each Data point represents one individual mouse [**(A, C)** n=4, two independent experiments, **(G**) n=5, two independent experiments, **I, J K**: n ≥ 5, 2–5 independent experiments]. Statistical testing was performed with multiple paired *t*-test for **(G)**, multiple unpaired *t*-test for **(I, J, K)** or paired *t*-test for **(A, C)**, using Prism v9 (GraphPad). *p*-Values are shown as * < 0.05, ** < 0.01, *** < 0.005, and **** < 0.001 where each statistical significance was found.

Supplementary Figure 5 GFI1 intrinsically controls the generation of γδ T cells. **(A)** *Gfi1b* mRNA expression in γδ T cells from thymus, spleen, LN, and lung. *Gapdh* was used as the housekeeping gene. Each Data point represents one individual mouse; n=3, one experiment. **(B)** Representative plots showing CD45.1/2^+^ donor γδ T cells and CD45.2^+^ recipient γδ T cells in the spleen, thymus and lung of non-irradiated WT and KO recipients after gating on live, singlets cells. **(C, D)** Representative plots showing IL-17A^+^ (C) and RORγt^+^ (D) cells previously gated on CD45.1/2^+^ donor γδ T cells or CD45.2^+^ recipient (WT and KO) γδ T cells from the spleen, thymus and lung of non-irradiated WT and KO recipients after gating on live, singlets cells. **(E)** Representative plots showing Vγ1 and Vγ4 expression in CD45.1/2^+^ donor γδ T cells or in CD45.2^+^ recipient (WT and KO) γδ T cells from the spleen, thymus and lung of non-irradiated WT and KO recipients after gating on live, singlets cells.

Supplementary Figure 6 Precursors in thymus of adult mice. **(A)** Scheme showing the culture of sorted thymic DN cells from WT and KO mice on an OP9-DL1 layer in the presence of Flt3 (5 ng/mL) and IL7 (1 ng/mL) for 12–14 days. **(B)** Representative plots showing the purity of the thymic DN population before selection (total thymocytes) and after selection. **(C)** Representative plots showing the purity of the thymic DN population after selection from WT and GFI1 KO thymi. **(D)** DN cell frequencies in the thymus of WT and GFI1 KO mice or control and *vav-cre GFI1^flox/flox^* mice. DN1a/b cells were characterized as CD4^-^CD8^-^γδT^-^CD44^+^CD25^-^CD24^+^Ckit^+^, DN1d cells were characterized as CD4^-^CD8^-^γδT^-^CD44^+^CD25^-^CD24^+^Ckit^-^, DN1e cells were characterized as CD4^-^CD8^-^γδT^-^CD44^+^CD25^-^CD24^-/low^Ckit^-^, DN2 cells were characterized as CD4^-^CD8^-^γδT^-^CD44^+^CD25^+^ and DN3 cells were characterized as CD4^-^CD8^-^γδT^-^CD44^-^CD25^+^. **(E)** Dot plot showing the key differentially expressed genes in each cluster shown in **Figure 6C**. Color represents the mean expression of the gene in the respective cluster, and dot size represents the fraction of cells in the cluster expressing the gene. **(F)** Violin plots showing the number of reads and genes as well as the percentage of mitochondrial genes quantified per cell in the thymic DN cells in the scRNA-seq data. **(G)** GFP^+^ cells indicating Gfi1 expression in the indicated thymic DN populations from GFI1:GFP reporter mice (het) and controls (wt). **(H)** Differential gene expression showing key up- or down- regulated genes in GFI1 KO DN3a, DN3a→b and DN3b cells versus WT cells. **I**: c-MAF % in DN1a/b, DN1d, DN1e, DN2 and DN3 cells for WT and GFI1 KO mice or control and *vav-cre GFI1^flox/flox^* mice analyzed by flow cytometry. Each Data point represents one individual mouse [**(D)** left panel: n=6, two to three independent experiments, **(D)** right panel: n=3, on experiment**, (I)** left panel: n=6, two to three independent experiments, **(I)** right panel: n=3, on experiment]. Statistical testing was performed with multiple paired *t*- test using Prism v9 (GraphPad). *p*-Values are shown as * < 0.05, ** < 0.01, *** < 0.005 and **** < 0.001 where each statistical significance was found.

Supplementary Figure 7 Potential of differentiation of precursors in thymus in adult mice. **(A)** Representative plots of splenocytes and thymocytes from control and *Cd2-cre* Gfi1^flox/flox^ mice analyzed by flow cytometry and stained to identify γδ T cells (CD45^+^CD4^-^γδT^+^) after gating on live cells (according to the FSC/SSC) and singlets. **(B)** Cell numbers and frequencies in CD45^+^ cells of γδ T cells (CD4^-^γδT^+^) in the spleen and in the thymus of control and *Cd2-cre* Gfi1^flox/flox^ mice. **(C, D)** Cell counts of Vγ1^+^ γδ T cells, Vγ4^+^ γδ T cells and Vγ1^-^Vγ4^-^ γδ T cells in the spleen **(C)** and in the thymus **(D)** of control and *Cd2-cre* Gfi1^flox/flox^ mice. **(E)** Representative plots of splenic and thymic cells from control and *Cd2-cre* Gfi1^flox/flox^ mice treated with PMA, ionomycin and GolgiPlug for 3.5 hours and analyzed by flow cytometry. The cells were gated on singlets CD45^+^CD4^-^γδT^+^ before gating for IFN-γ and IL-17A. **(F)** Representative normalized histogram of RORγt^+^ cells in either splenic or thymic CD45^+^CD4^-^γδT^+^ and CD45^+^CD4^+^γδT^-^ from control and *Cd2-cre* Gfi1^flox/flox^ mice. **(G)** Frequencies of IL-17A^+^ cells in γδT^+^ cells and CD45^+^CD4^-^γδT^+^IL-17A^+^ cell counts in the spleen and thymus of control and *Cd2-cre* Gfi1^flox/flox^ mice. **(H)** Frequencies of RORγt^+^ cells in γδT^+^ cells and CD45^+^CD4^-^γδT^+^RORγt^+^ cell counts in the spleen and thymus of control and *Cd2-cre* Gfi1^flox/flox^ mice. **(I)** Scheme showing the culture of sorted thymic DN1 or DN2/3 cells from WT and KO mice on an OP9-DL4 layer in the presence of Flt3 (5 ng/mL) and IL7 (1 ng/mL) for maximum 20 days. **(J)** Representative plots showing the gating strategy to sort DN1 and DN2/DN3 for the coculture with the OP9-DL4 cells described in **Supplementary Figure 7I**. Each Data point represents one individual mouse [**(B, C, D)** n=9, three independent experiments, **(G)** n=5, two independent experiments, **(H)** n=7, three independent experiments]. Statistical testing was performed with paired *t*- test **(G)** using Prism v9 (GraphPad). *p*-Values are shown as * < 0.05, ** < 0.01, *** < 0.005 and **** < 0.001 where each statistical significance was found

Supplementary Figure 8 GFI1 regulation in adult mice. Peak for GFI1 on *Maf* promoter from ChIP-seq analysis from Mast cells (GSE42518), ILC2 cells (GSE50806) and Th1 cells (GSE70893).

Supplementary Table 1 Differentially expressed gene in *Trgv6+* cells from WT and GFI1 KO spleens.

Supplementary Table 2 Differentially expressed gene in *Trgv1+* cells from WT and GFI1 KO spleens.

Supplementary Table 3 Differentially expressed gene in *Trgv4+* cells from WT and GFI1 KO spleens.
