# Supplementary material for: GFI1 as a novel regulator of γδ T cell development and the IL-17/IFN-γ lineage commitment
Source: Front Immunol. 2026 Aug 5;17:1849622. doi: 10.3389/fimmu.2026.1849622 (PMC13485770; doi:10.3389/fimmu.2026.1849622)
Supplement: Supplementary file 1 [file DataSheet1.pdf]

Column1	Column2	Column3	Column4
names	scores	logfoldchanges	pvals
Zeb2	34.71703	3.46316	4.360101607329874e-264
Tmeff2	33.597855	5.4765162	1.803132281149191e-247
Maf	28.19928	0.98235536	5.967746560362195e-175
Slc15a3	26.631077	4.4427714	2.9650159890260066e-156
Clnk	26.62269	1.2344191	3.7080450815251984e-156
Cxcr6	25.33069	0.7810676	1.4669389554121785e-141
Prickle1	25.17707	2.9191146	7.14320769352578e-140
Capg	22.8175	0.98842454	3.0737568443192613e-115
Ppp1r14c	21.533504	2.0601268	7.559495747549292e-103
Atg7	21.419853	1.4414544	8.726237598855128e-102
Myo16	21.125294	5.275096	4.657293253944184e-99
Icos	20.967031	1.4730823	1.31196694046309e-97
Gzmb	20.68243	2.778538	4.986520346483432e-95
Npnt	20.324755	1.8809469	7.767749252980625e-92
Tmem176a	19.85345	0.79827374	1.0288610613874225e-87
Itgb5	19.78057	1.9446692	4.377283846704664e-87
Blk	19.518116	0.69897866	7.702660590478608e-85
Ltb4r1	19.499825	1.21294	1.1015919902042373e-84
Arnt2	19.477787	1.6990113	1.6944598650421463e-84
B2m	19.474398	0.39064878	1.8104129615786947e-84
Mcc	19.391254	4.713424	9.147957052403234e-84
Ccnd2	19.133585	0.9948411	1.3263344974424289e-81
Jund	19.116947	0.88795495	1.8247839084478027e-81
Dnaja1	18.889439	0.7496603	1.3932140348909282e-79
Lgals1	18.13108	0.7708711	1.8116140587788193e-73
AU020206	18.078976	0.9426729	4.6666400781324225e-73
Tmem163	17.924747	1.6036736	7.559323792520477e-72
Hopx	17.820024	1.2862333	4.941568905032813e-71
Havcr2	17.775366	2.6646476	1.0967865563832861e-70
Tmem176b	17.736645	0.6820641	2.1860354825817032e-70
Chsy1	17.660109	1.4135551	8.507629801655614e-70
Ikzf2	17.561005	0.72293204	4.89996737728041e-69
Fgl2	17.25277	1.0373836	1.066612146103947e-66
Plcb4	17.233156	0.88840294	1.4975157713862657e-66
Calm1	17.116879	0.43434766	1.1107217870209561e-65
Pkib	16.682714	2.3897388	1.7507902872641477e-62
Hspa5	16.353184	0.904313	4.1283075077022906e-60
Ccdc50	16.284342	1.0178399	1.2749315736342194e-59
Mgat5	16.256815	0.8529248	1.9986247233198363e-59
Arl6ip1	16.210426	0.56582505	4.256120627404358e-59
Manf	16.15894	0.75896066	9.82387438449052e-59
Nbea	15.99684	2.3557613	1.3442790542520525e-57

Ablim3	15.961752	1.7503706	2.3601390830285392e-57
Scart1	15.611515	0.39541814	6.0776663535061365e-55
Cfl1	15.197781	0.41638824	3.658082699687067e-52
Osbpl3	15.183794	0.71687186	4.528172905331662e-52
Ly6g5b	15.159962	1.3170106	6.510710942610722e-52
Hsp90b1	15.089231	0.5575986	1.9065403882242512e-51
Atrnl1	15.012423	0.9256099	6.088244531492186e-51
Selenop	14.633761	0.6306838	1.7106198652522334e-48
Ostf1	14.586169	0.5586448	3.439769610432878e-48
Sdf2l1	14.570507	1.1468949	4.326659864967776e-48
Edil3	14.5306425	3.8445363	7.749061227620158e-48
Sec11c	14.527585	0.6236494	8.10268106459478e-48
Actb	14.371654	0.34086388	7.794748956173352e-47
Abi3bp	14.275729	0.85491097	3.100263824573538e-46
Mbd2	14.252976	0.56633353	4.295762689324903e-46
Plek	14.183017	2.2638526	1.1671905136731728e-45
Selenom	14.118158	1.5650615	2.9356380451962803e-45
Jaml	13.951999	0.6793879	3.059124459813758e-44
Vim	13.921396	0.6436226	4.696401032987562e-44
Etv6	13.684974	0.8223553	1.248522309692791e-42
Tnf	13.296836	1.482081	2.4147369572945257e-40
Gcnt2	13.291434	1.1220576	2.595535037297143e-40
Ccr1	13.263458	3.715188	3.770949460202713e-40
Lat	13.19866	0.59732765	8.931023001719223e-40
Miat	12.973523	1.5816283	1.728966296258984e-38
Plec	12.940001	0.79711217	2.676271006758077e-38
Sema4b	12.871167	1.1341531	6.540620237330611e-38
Hsp90ab1	12.845016	0.34899262	9.173385115202262e-38
Arhgap6	12.788249	2.7667964	1.9072224155457676e-37
H2-D1	12.574226	0.26233968	2.9264988216724573e-36
Pdia6	12.514041	0.7335895	6.255869436533569e-36
Cd44	12.497598	0.5204984	7.694122444229004e-36
Tagln2	12.319047	0.47856742	7.153488645625106e-35
Fryl	12.232121	0.50750136	2.0939839160702837e-34
Hsd17b13	11.965851	3.3327055	5.364470165369519e-33
Prdx1	11.959419	0.54285073	5.7965859114581476e-33
Anp32a	11.959214	0.44687226	5.8109118359293376e-33
Ppp1r14b	11.958397	0.7149878	5.8683580961271576e-33
Trpm6	11.955343	1.8391974	6.088141953737091e-33
Mir142hg	11.860747	0.29349378	1.8927865053652873e-32
Cd48	11.80604	0.4452548	3.6327232362177754e-32
Aprt	11.783233	0.63694555	4.763048056579504e-32
Hsp90aa1	11.759805	0.6408889	6.28792030268757e-32
Fkbp5	11.703785	0.7467636	1.2189298574102384e-31

Calr	11.641216	0.599128	2.5436073975466264e-31
Wls	11.633582	0.71842813	2.781752456580073e-31
Cish	11.60639	1.862288	3.8242236013477687e-31
Tmtc2	11.478464	1.0579761	1.6926003808849806e-30
Cox17	11.457318	0.60763776	2.1610298189462906e-30
Wdfy2	11.42228	0.8047667	3.2362849501971874e-30
Pdcd1	11.399735	1.3231239	4.193930898557579e-30
Cd247	11.361195	0.4222646	6.524602937202247e-30
Txn1	11.354163	0.5182599	7.07138176750596e-30
Pram1	11.3476305	2.3391798	7.619996281160092e-30
Klrb1b	11.302359	1.4641284	1.2774091717926598e-29
Dennd5a	11.261159	0.9527498	2.040568739327318e-29
Pkp3	11.174718	0.7492853	5.422249846497106e-29
Tpm3	11.168895	0.41497475	5.789667525485587e-29
Cdh10	11.157942	0.88276863	6.549034779557679e-29
Ezr	11.112007	0.64452714	1.0966649245950377e-28
Diaph1	11.09814	0.42578733	1.280825544317929e-28
Ern1	11.031116	0.9704091	2.704901824110033e-28
Pdia4	11.025207	0.7333788	2.888558296744215e-28
Pdia3	10.948408	0.4558751	6.762571441801534e-28
Il1r1	10.923035	0.52332306	8.945628524319384e-28
Zmiz1	10.906163	0.7533603	1.0770683852482718e-27
Fut7	10.886544	1.8598436	1.3361492017309328e-27
Lrrfip1	10.840482	0.5693936	2.2130264457262077e-27
Pon2	10.806128	0.6957087	3.2197724747939675e-27
Nfic	10.626406	0.8005413	2.246023839660142e-26
Cpe	10.582807	1.7579837	3.5806993661865657e-26
Hsph1	10.550728	0.9062953	5.040524620106707e-26
Stmn2	10.537	1.3883808	5.833030466486265e-26
Cmtm7	10.499867	0.54184294	8.650150681845552e-26
Dynll1	10.454153	0.4571992	1.4024661219966403e-25
Far1	10.452602	0.78488404	1.4255940685021276e-25
Zfp36l1	10.275412	0.49536297	9.095544556315813e-25
Mast3	10.275254	0.7635369	9.110409009673218e-25
Anxa2	10.1255045	0.59879154	4.257757443906323e-24
Ech1	10.115969	0.7400849	4.693513273614653e-24
Snd1	10.088334	0.70188534	6.221634148877678e-24
Tgfbr3	9.828009	2.0340922	8.528838992412569e-23
Surf4	9.827809	0.66990215	8.545708958016033e-23
Gng2	9.715359	0.51129615	2.593336377938505e-22
Atf6	9.673284	0.56200665	3.9160782145271864e-22
Rom1	9.666903	0.9406362	4.168035286354026e-22
Plin2	9.658798	1.2079761	4.511264526353767e-22
Cd52	9.59104	0.26353586	8.72016939239201e-22

Grk3	9.496327	1.2817754	2.174227536386033e-21
Arhgef11	9.482252	1.0994017	2.4885333284489135e-21
Ddx21	9.445905	0.64792037	3.523485883047754e-21
Rexo2	9.435473	0.45734346	3.892311138197165e-21
Eif4g2	9.432028	0.42376736	4.022367055828099e-21
H2-K1	9.402498	0.2441199	5.328281740210462e-21
Tpm4	9.3883095	0.5686343	6.097102659128674e-21
Pfn1	9.367769	0.22499284	7.408165241832737e-21
Fkbp1a	9.317548	0.44292918	1.1906006868523936e-20
Ptpn7	9.272647	0.6917639	1.8158234106858562e-20
Rbpj	9.213243	0.71423966	3.1641769284153665e-20
Emp3	9.2114935	0.3443894	3.216193432063374e-20
Selenos	9.157636	0.6704964	5.304683807258315e-20
Mast4	9.152047	1.1941359	5.586454139108859e-20
Sdc4	9.147582	2.4497573	5.822191404797794e-20
Ccr2	9.11496	0.31005242	7.869952950801369e-20
Arpc1b	9.111528	0.3479179	8.122923200941484e-20
Iqgap1	9.109615	0.36598596	8.267445876710991e-20
Cdc42	9.102473	0.24073258	8.82974078318444e-20
Rilpl2	9.072531	0.71263164	1.1628391814850877e-19
Pde6d	9.051486	0.77141654	1.4103599335653336e-19
Popdc2	9.034469	2.409853	1.6480048444509808e-19
Rorc	9.018813	0.44224027	1.9013836296415944e-19
Satb1	9.018192	0.69208664	1.9121890072451976e-19
Sdccag8	8.948012	0.55191696	3.619391072191621e-19
Cdkn2d	8.941421	0.7084388	3.8419915563382864e-19
Rhoa	8.940491	0.28428325	3.8744598484851483e-19
Cd80	8.918943	2.4112008	4.70757452281952e-19
H2-Q7	8.833577	0.4685389	1.0138002452206662e-18
Tmed10	8.80146	0.50901544	1.3504736317276986e-18
Tmsb4x	8.7668915	0.13155335	1.8366592070462855e-18
Septin11	8.738497	0.37163478	2.3623215483146074e-18
Wnk1	8.724276	0.40789458	2.678895149471772e-18
Dlgap1	8.723037	4.684868	2.7083782619124163e-18
Pou2f2	8.711248	1.4155169	3.005451409947249e-18
Slco3a1	8.6972475	0.47356853	3.400314734290672e-18
Cst7	8.670614	0.84873027	4.2979566728926195e-18
Cap2	8.669266	1.9613818	4.34916957095486e-18
Hnrnpl	8.659355	0.49548265	4.744417215498605e-18
Fmn1	8.624219	1.3430779	6.453141970477587e-18
Akirin1	8.6139965	0.43691126	7.055675111977006e-18
Rrbp1	8.593691	0.5653892	8.421914846981113e-18
Socs2	8.56917	0.8124645	1.0423335540712852e-17
Alcam	8.5506	1.4039578	1.2245055390041553e-17

Aim2	8.5294895	0.71405154	1.4699527463687043e-17
Cox5a	8.522172	0.33958262	1.5658840719488225e-17
Igsf9b	8.464853	1.9279704	2.564748586112917e-17
Hip1	8.4254875	0.6302542	3.592516028220537e-17
Gng5	8.35324	0.30743542	6.641523416841303e-17
Creld2	8.332715	1.2193313	7.900905106243655e-17
Pkm	8.303206	0.45057115	1.0133873073020686e-16
Ppm1g	8.272857	0.55881584	1.3078703300208067e-16
Aopep	8.258855	0.5322852	1.47076275593454e-16
Cacybp	8.258561	0.5523593	1.4743905945216576e-16
Crybg3	8.2201605	1.0143162	2.0323107827040857e-16
Niban2	8.164395	1.420612	3.2304872981033573e-16
Psd2	8.159357	1.7416338	3.3681259077257997e-16
Nucb2	8.144651	1.5713407	3.803755323430489e-16
Rora	8.130674	0.35080162	4.269101122803732e-16
Unc119	8.125469	0.72258496	4.456353962895006e-16
Hcls1	8.10678	0.51425797	5.197892897350638e-16
Ywhaz	8.095684	0.25298572	5.694352727546058e-16
Actr2	8.090025	0.30326438	5.965273271309186e-16
Bcl2a1a	8.066448	0.7123943	7.237275793462695e-16
St3gal3	8.056716	0.386141	7.837153082785286e-16
Runx2	8.051841	0.37802798	8.15579607104303e-16
Pi4k2a	8.045338	0.56595594	8.60079182870121e-16
C030034L19Rik	8.031716	0.52482414	9.611821466045782e-16
Cd72	8.026633	1.6274445	1.0018377693385659e-15
Dnajb11	8.020137	0.50629073	1.0562781372370528e-15
Selenok	7.987847	0.35698605	1.3731600043453032e-15
Cerk	7.9407268	0.61575764	2.0099985442162385e-15
Gpr55	7.9324503	3.030138	2.1486387114206454e-15
Acot9	7.9012923	0.53181803	2.7602618296660015e-15
Spcs2	7.8911915	0.28738126	2.9931442219135454e-15
Arl15	7.8770776	0.4282658	3.351272250680668e-15
Dnaaf9	7.868245	0.9050586	3.596500222415388e-15
Hspa8	7.8562727	0.21032506	3.957323201818943e-15
Camk2n1	7.8394732	1.0293319	4.524411065040461e-15
Ptpn1	7.8372984	0.44719234	4.603431670629375e-15
Arhgap23	7.832652	1.6055961	4.77684870704808e-15
1810037I17Rik	7.796652	0.4351013	6.3571288297115446e-15
Ubl3	7.77566	0.5639354	7.505491900642696e-15
Gpr65	7.74862	0.9588072	9.289657210268668e-15
Susd3	7.746217	1.1597608	9.467096694291615e-15
Fkbp3	7.743626	0.4206498	9.662089814446168e-15
Rab8b	7.7415586	0.42465317	9.820558113944697e-15
Rala	7.7287574	0.47179484	1.0860158161443016e-14

Drap1	7.702142	0.4451751	1.3380429547659968e-14
Atp2a2	7.6603456	0.66560096	1.8543342818418974e-14
Slc1a5	7.6546745	0.5127317	1.9380276065698734e-14
Pgam1	7.6456995	0.58470726	2.078124282033046e-14
Lnx2	7.6405005	1.0199966	2.163787437673142e-14
Smad3	7.5980897	0.56162506	3.005345483061369e-14
Smox	7.586236	0.62967473	3.293324387626142e-14
Myl12a	7.5831966	0.31570137	3.37142762995572e-14
St13	7.570835	0.5076835	3.7083188122043237e-14
Jpt1	7.5603366	0.46936423	4.020278891247784e-14
Ankrd12	7.548088	0.35299236	4.4169479067497434e-14
Cmip	7.5212407	0.47888684	5.4258823082978446e-14
Pten	7.4505715	0.35138783	9.293686937229165e-14
Il2rg	7.4501348	0.3065226	9.324502803575193e-14
Clic1	7.4187717	0.23003542	1.182113122000423e-13
Gnaq	7.4049487	0.6323043	1.3120113905148140e-13
Spn	7.352924	0.376077	1.9391711825563975e-13
Arfgef1	7.32168	0.47225678	2.448852995371932e-13
Cib1	7.291966	0.67443883	3.0546384408230955e-13
Set	7.2511563	0.28424695	4.1322853473349753e-13
Capn2	7.2490115	0.60653234	4.1982466792120465e-13
Dnajc1	7.2327876	0.35439825	4.731784047645435e-13
Klf7	7.223653	0.9465411	5.06094474132731e-13
Il27ra	7.185335	0.4006562	6.704249703138636e-13
Xbp1	7.1578436	0.64182985	8.195609500770095e-13
Arpc2	7.138373	0.20389706	9.444222695132933e-13
Zfp36	7.126314	0.69987595	1.0309217343769994e-12
Bzw1	7.090745	0.3189208	1.3339205274882063e-12
Sytl3	7.0754747	1.6356908	1.489377539851317e-12
Arrb2	7.064804	0.7225218	1.6084303968711306e-12
Plk3	7.0558677	1.614882	1.7152700370885308e-12
Coro2b	7.052668	0.54107267	1.755191496954717e-12
Ankrd44	7.0466495	0.24935974	1.832774985910935e-12
Tanc1	7.039674	0.968574	1.9269035639974674e-12
Tmed9	7.0353127	0.3321229	1.988146300629445e-12
Trim14	7.018245	0.5515337	2.2467163213823218e-12
Serpinb6a	7.004541	0.4874606	2.4779662156231477e-12
Trgc1	6.972837	0.21485437	3.106130271633622e-12
Stk10	6.96527	0.33754465	3.277742673861267e-12
Pld1	6.950523	1.9490958	3.6393434064903665e-12
Eid1	6.9418454	0.5041324	3.870108076530952e-12
Stip1	6.9382358	0.5608509	3.97026615135156e-12
Naga	6.933423	0.7702199	4.107772217279235e-12
Inpp4a	6.922532	0.8763017	4.436410580459985e-12

Bcl2a1d	6.9202	0.65181506	4.510062205549715e-12
Lcp1	6.888026	0.2634035	5.657175746314926e-12
Ndufa1	6.8740726	0.48382503	6.239449902188664e-12
Mapre1	6.8715324	0.4718294	6.3515860395915885e-12
Ddx5	6.8612595	0.18706438	6.825610368112035e-12
Canx	6.8527894	0.3595211	7.242356186946113e-12
St3gal1	6.8519044	0.5629148	7.287329614566952e-12
Gpx1	6.8507247	0.2996196	7.347669255682112e-12
Gnai2	6.8331943	0.24970396	8.304440827613935e-12
Tbcb	6.8050427	0.49128968	1.0101913540282727e-11
Pfkp	6.7885313	0.4829343	1.1328090296220467e-11
Gmds	6.775828	0.58320725	1.2369574425928446e-11
Misp3	6.7723403	1.505764	1.2671564119445055e-11
Ebpl	6.770522	0.76286113	1.2831841224180046e-11
Myo1e	6.763184	0.38297158	1.349913396780562e-11
Hmgn1	6.7625012	0.7396046	1.3562973082655186e-11
Specc1	6.756666	1.3231119	1.4120283494589917e-11
Fkbp2	6.746601	0.6320682	1.5134843104519684e-11
Lgals3bp	6.736052	0.56048024	1.6274810094630658e-11
Ahr	6.72913	1.2274156	1.7068051558428263e-11
Lrrc17	6.696359	3.1907592	2.1367629486511377e-11
Rac1	6.696068	0.3052576	2.141022019836286e-11
Adam12	6.677064	0.32154834	2.437769612748683e-11
Nrbp1	6.6400356	0.634753	3.136072737945039e-11
Slc16a6	6.6336365	0.67222637	3.2751569735540676e-11
Purb	6.619255	0.3637211	3.610138729958173e-11
Hnrnpll	6.6162367	0.5521935	3.684585678565966e-11
Hnrnpa2b1	6.6142936	0.24319991	3.733299802549058e-11
Phf11b	6.6132927	0.58809966	3.758647209783454e-11
Ptgdr2	6.6115932	3.1426032	3.802056135790233e-11
Myo5a	6.608997	0.97868836	3.8693328257024804e-11
Cpd	6.561232	0.83002436	5.336495434384962e-11
Rara	6.545407	0.42223126	5.933368518756930e-11
Tln1	6.5224957	0.30057755	6.914695660215488e-11
Arf1	6.5058656	0.33005172	7.724739795155793e-11
Rpn1	6.50457	0.5265384	7.79158887872705e-11
Tram1	6.499368	0.3687965	8.06580285054841e-11
Calm2	6.4945464	0.30613613	8.328385259760672e-11
Trpv2	6.4721823	0.43819436	9.659741285353808e-11
Cmtm6	6.471541	0.46478847	9.70086168498762e-11
Ddit4	6.4570994	0.7227096	1.0672863240842184e-10
Gatad2a	6.4508014	0.48070967	1.1126012090281332e-10
Flna	6.45062	0.36085787	1.1139325334860311e-10
Zc3h12c	6.445068	3.3192928	1.1554868011697292e-10

Rnf43	6.4238534	0.43304494	1.3286717025213748e-10
Arhgdia	6.422766	0.354225	1.3382004893546306e-10
Esyt1	6.4085836	0.35861975	1.4687785264529845e-10
Sat1	6.408295	0.40411407	1.4715567160562964e-10
Mat2a	6.395295	0.47387898	1.602376034817067e-10
Socs1	6.3905478	0.6597833	1.652925864994819e-10
Bcl2a1b	6.3683143	0.41911253	1.9111702410210797e-10
Trgv6	6.3639054	0.40604728	1.9668706082329052e-10
Fmnl1	6.358971	0.38118705	2.0310962394681177e-10
Magt1	6.3500676	0.48839447	2.1522033289639215e-10
Nxpe3	6.349399	0.561652	2.1615748950564258e-10
Marchf2	6.3399816	0.4939249	2.2979241427264822e-10
Tmed2	6.3186574	0.30214408	2.6384562232077867e-10
Rap1gds1	6.3162184	0.36021775	2.6804137855494616e-10
Podnl1	6.3073325	0.4190378	2.8388531969394025e-10
Add3	6.28908	0.37120393	3.1935284833628193e-10
Map4	6.2883554	0.37072024	3.208472646642841e-10
Akirin2	6.282283	0.4899508	3.336371774413558e-10
Ssb	6.272809	0.32178846	3.5459194527194394e-10
Med10	6.265183	0.5552187	3.7238809543386335e-10
Ywhah	6.2423344	0.32809255	4.310879457101749e-10
Lat2	6.2338467	2.3150191	4.5511798481380257e-10
Cyb5a	6.2254333	0.32396334	4.802261317802488e-10
Slc25a4	6.215508	0.3850139	5.115883042965926e-10
Myl12b	6.213705	0.21632656	5.174969585251209e-10
Ptges3	6.2055025	0.30105463	5.452242120075221e-10
Timm23	6.200455	0.29790935	5.630013490430389e-10
Adam8	6.1981378	0.57674956	5.713509611912613e-10
Itgb2	6.1901283	0.4444608	6.01151675067793e-10
Rap1a	6.1693	0.29196456	6.859286228112062e-10
Srsf5	6.1669264	0.37544516	6.963006140682846e-10
Vcp	6.1610594	0.44051033	7.225994152925074e-10
Itm2c	6.149224	0.7719185	7.786315268665009e-10
Azin1	6.144631	0.5666537	8.01497566834699e-10
Sumo2	6.1274357	0.20610914	8.930665581602016e-10
Gnb2	6.1268415	0.31186604	8.964065050850757e-10
Syt11	6.1158795	0.75817204	9.602598233580408e-10
Haspin	6.1014442	1.1220758	1.051143467899882e-09
Tuba1a	6.078682	0.6461084	1.2117446819690273e-09
Farp1	6.0561633	0.6537212	1.394065481078399e-09
Adprh	6.051083	0.564953	1.43875151992308e-09
Mgat2	6.0433946	0.51036435	1.5090485733782316e-09
Nedd9	6.0428925	0.39304194	1.513753721626308e-09
Cltc	6.0420847	0.42521948	1.5213565447606353e-09

Pitpna	6.039601	0.30876082	1.5449577519922992e-09
Plxnd1	6.0218625	0.48331067	1.7242150317326709e-09
Hnrnpd	6.0211525	0.3421268	1.7317967509198045e-09
Txndc17	6.0187073	0.39682162	1.758153867632331e-09
Rnf17	6.016637	1.0563366	1.7807811044603548e-09
Hdlbp	5.98454	0.49213675	2.170020294358764e-09
Sh3bgrl3	5.967541	0.20175502	2.408554167606115e-09
Riox2	5.9584684	0.42946672	2.546128410443652e-09
Pou6f2	5.952233	3.4784758	2.6450891327808597e-09
Mapkapk3	5.949042	0.62678343	2.697163261233013e-09
Zfp609	5.933362	0.90692216	2.9679315827257406e-09
Arl5a	5.929714	0.64139277	3.034627248025413e-09
Ap1s2	5.9239893	0.4504837	3.1422392281865262e-09
Sfxn1	5.921806	0.6369322	3.1842560789468264e-09
Hacd4	5.9195538	0.77266383	3.228160579293822e-09
Zbtb7c	5.9187636	2.8264453	3.2437069612136575e-09
Kif5b	5.917085	0.31986678	3.2769706417396366e-09
Snx3	5.9068832	0.3377741	3.4864028698837034e-09
Kin	5.8996525	0.6214389	3.6426798227037934e-09
Inpp5d	5.8718905	0.31367072	4.308537044994205e-09
Ptpn22	5.864214	0.4145345	4.512657323924856e-09
Hydin	5.8543744	4.880533	4.788086088566192e-09
Tnfaip8	5.85209	0.3419475	4.854339401414741e-09
Plekho2	5.8510323	0.6018051	4.885311890001478e-09
Malat1	5.8442707	0.2254437	5.087920192017246e-09
Cyba	5.8241496	0.2637813	5.740415165156352e-09
Auts2	5.8162947	0.76729316	6.016633246808925e-09
Diaph2	5.8034163	0.37758034	6.497718008146353e-09
Matn2	5.8001604	0.5416989	6.625153964348053e-09
Ybx3	5.795092	0.4181588	6.828367554197967e-09
Arpc5l	5.78402	0.40362844	7.293627512742032e-09
Itga5	5.7821097	0.6502017	7.376955215725748e-09
Bbln	5.7683163	0.5401914	8.006741584060625e-09
Sla	5.767574	0.28753388	8.04209581567284e-09
Cyp2j6	5.7621818	2.769871	8.303357843888989e-09
Uso1	5.7474375	0.5363077	9.060605985613547e-09
Rhof	5.736086	0.62806034	9.688942423487722e-09
4930503L19Rik	5.726214	0.71970624	1.0269659005207544e-08
Hspd1	5.701488	0.41147608	1.1876600162155734e-08
Adrb2	5.6954784	0.89556605	1.2302660079643527e-08
Cklf	5.680348	0.42891756	1.3442103153033476e-08
Tubb4b	5.6741867	0.5587649	1.3934927797162412e-08
Morf4l2	5.635765	0.5822067	1.7428281548254267e-08
Prr13	5.627851	0.22828943	1.8246872583954715e-08

1700097N02Rik	5.61117	0.7281418	2.0096342011750054e-08
Kmt2a	5.605475	0.28071752	2.0768483805623918e-08
Cnn2	5.572264	0.30407917	2.51449688222811e-08
Mier1	5.5686607	0.27736056	2.567049152534736e-08
Ptpre	5.563836	0.4938568	2.6390791200656015e-08
Vsir	5.559041	0.3680743	2.712607079946258e-08
Actn4	5.5515876	0.41752708	2.830870688025107e-08
Il12rb1	5.550087	0.63783175	2.8552718258038215e-08
Casp1	5.541038	0.95781314	3.006837563199983e-08
Frmd4a	5.5217695	1.0166497	3.3560257477212946e-08
Bcl11b	5.496102	0.21263462	3.882784873399452e-08
Smarca4	5.4958253	0.45448413	3.88886945162826e-08
Syncrip	5.4944706	0.355754	3.918837579432078e-08
Nxnl2	5.492516	0.55567557	3.962476114785172e-08
Adgrg5	5.467386	0.7798023	4.56720750321792e-08
Nktr	5.4491453	0.3223348	5.061245864798423e-08
Calm3	5.429006	0.3467298	5.666871049528638e-08
Fat3	5.420097	3.1426516	5.956677889604201e-08
Igsf11	5.4134274	3.8095853	6.182969314084352e-08
Cers2	5.4103494	0.5581204	6.290183779036055e-08
Gnb1	5.404696	0.22000259	6.491834810239942e-08
Tspo	5.403906	0.15444086	6.52051561725597e-08
Dok2	5.3943725	0.39527017	6.876339101234014e-08
Arhgap26	5.389762	0.22925428	7.055108334348035e-08
Ehbp1l1	5.3561916	0.6726018	8.499425231577465e-08
Skap2	5.345937	0.9333574	8.995066134026478e-08
Peds1	5.3413467	0.6455812	9.225857908577903e-08
Abhd15	5.3412814	0.5259464	9.229185305361783e-08
Sgip1	5.3370094	4.389807	9.449214122575292e-08
Mafg	5.3341513	0.9149474	9.599235884734538e-08
Tuba4a	5.296948	0.609963	1.1775411755006964e-07
Fabp5	5.294907	1.1015817	1.1907686339545325e-07
Ptpn6	5.286396	0.36334205	1.2474978370164812e-07
Arhgap30	5.284156	0.27653283	1.262856940321269e-07
Septin7	5.2416587	0.268858	1.5913964608549135e-07
Rap1b	5.230046	0.20019424	1.6946814841671205e-07
Nherf1	5.219003	0.3517816	1.798886336194719e-07
Cd82	5.218326	0.2742335	1.8054759656026404e-07
Prc1	5.214895	1.2390138	1.8392177885249635e-07
Crybg1	5.20671	0.6356675	1.9221809034832035e-07
Itm2b	5.2054296	0.12358367	1.9354828333143836e-07
Rabgap1l	5.1959677	0.21396449	2.0365770023838306e-07
Ppfibp1	5.17456	1.5736693	2.2844865345746317e-07
Hyou1	5.170279	0.79811233	2.3374462212993614e-07

Plxdc1	5.1490054	0.7479488	2.6187162854182144e-07
Il2ra	5.1185603	0.36151615	3.0787660728750527e-07
Psd4	5.0980024	0.4446621	3.432563341161468e-07
Dnajb9	5.0897317	0.82398695	3.5857036951103036e-07
Coro1c	5.084943	0.5336445	3.6773681926922184e-07
Lrrc4	5.083977	0.5877807	3.6961209322437385e-07
Fermt3	5.0746017	0.30525023	3.883089771151067e-07
Prep	5.072436	0.73998255	3.9275575048503004e-07
Cbfb	5.0710216	0.3643127	3.9568574228466615e-07
Il17a	5.063951	2.9006155	4.106549265141816e-07
Man2a1	5.0500894	0.29230428	4.4160337704120366e-07
Rap1gap2	5.046839	0.32328737	4.491787782365022e-07
Vamp8	5.044525	0.3306392	4.546492113909648e-07
Rsu1	5.0436397	0.48149413	4.567588280782932e-07
Cks2	5.0379	0.67265856	4.706667590931573e-07
Eif4e3	5.0318723	0.4076274	4.857124385278637e-07
Mob1a	5.026112	0.3078848	5.005235179303346e-07
Ubxn4	5.0180373	0.28094047	5.220201408667601e-07
Lrrc8c	5.017779	0.34922633	5.227227199452219e-07
Pole4	5.0098586	0.36310932	5.446998748458571e-07
Fam20a	5.0019536	1.9929495	5.675228455792009e-07
Tab2	5.0003104	0.3127437	5.723802075801211e-07
Capns1	4.9829345	0.3336261	6.262718205565269e-07
Kcnab2	4.9600444	0.41980985	7.04770729246746e-07
Krtcap2	4.9527035	0.21641584	7.3189436233801e-07
Sike1	4.9516845	0.34068173	7.35737980929976e-07
Edem1	4.945401	0.5220796	7.598716010287347e-07
Arl4a	4.937309	0.53173476	7.920798082284382e-07
Macir	4.9353867	0.47413498	7.999209218853115e-07
Ran	4.934317	0.23477781	8.043161471672944e-07
Zbtb38	4.928759	0.56271935	8.275362928266068e-07
Nfatc2	4.916305	0.563942	8.819283454752001e-07
Iffo2	4.914187	0.76564467	8.915158337774118e-07
Uevld	4.9061184	1.0909992	9.289646054178106e-07
Fam78b	4.902078	0.49131316	9.482811420608374e-07
Laptm5	4.8928328	0.10705361	9.939475552332336e-07
Snx10	4.892212	1.4098161	9.97089186021405e-07
9330111N05Rik	4.8902664	1.0115554	1.0069970068613724e-06
Eif5	4.8796606	0.22958072	1.062686441007072e-06
Hspa4l	4.857341	0.69087416	1.1897271881551914e-06
Ccdc86	4.857023	1.2986027	1.1916379782269333e-06
Srsf2	4.8403	0.32292324	1.296430810448025e-06
Abracl	4.8348994	0.24115244	1.3321300424083303e-06
Hk1	4.8342576	0.4464457	1.336433848484354e-06

Msc	4.822033	1.3481617	1.4210254907785348e-06
Fry	4.808807	0.32466105	1.5183384443552247e-06
Srrm2	4.7914367	0.16873205	1.6559139247566946e-06
Szrd1	4.7900195	0.4121487	1.6676512814404407e-06
Zfp706	4.7891374	0.28790873	1.6749997975089284e-06
Tuba1b	4.7829223	0.69424295	1.7276498236996735e-06
Serp1	4.777132	0.41202298	1.7781293596466887e-06
Ackr3	4.77524	2.3133018	1.7949329504396085e-06
Maml2	4.767513	0.3750706	1.8651425953002376e-06
Spcs3	4.761684	0.3762884	1.9198417087645954e-06
Zap70	4.7607393	0.31201145	1.9288512119231228e-06
Slbp	4.722095	0.400295	2.334275200137323e-06
Impa1	4.7208	0.32991686	2.3491907837571606e-06
Bmp2k	4.691469	0.8439039	2.712502801941501e-06
Sppl2a	4.6886826	0.3326477	2.749696789856733e-06
Prkar1a	4.678757	0.19489211	2.886192315677772e-06
Cbx3	4.65901	0.25465363	3.1773362367773245e-06
Ptbp3	4.6559234	0.24748215	3.225317933505381e-06
Ybx1	4.6488647	0.15778701	3.3376688051609113e-06
Txnrd1	4.6257133	0.42490765	3.733113041944131e-06
Prf1	4.623987	1.2748159	3.7643289777779757e-06
Gatad1	4.609846	0.33129907	4.0296677913312e-06
P4hb	4.6019382	0.3947322	4.185777669819555e-06
Mxi1	4.5998497	0.77801716	4.227961816110368e-06
Sfi1	4.576282	0.7125477	4.733120220126443e-06
Zbtb16	4.559494	0.22870381	5.127697725259901e-06
Dnajb1	4.5553946	0.66674906	5.228735954557743e-06
Smpdl3a	4.5490103	0.2331096	5.389882474101827e-06
Ndst1	4.5386124	0.79326415	5.662560532044786e-06
Gpsm3	4.5248013	0.24702533	6.0452313789908586e-06
Itsn1	4.5217323	1.2380515	6.133559173407775e-06
Cyth4	4.515461	0.28393632	6.317918515123579e-06
Reep5	4.5101876	0.25675774	6.477026314580476e-06
Cisd1	4.507624	0.4393621	6.555762242661849e-06
Ccdc12	4.505381	0.21753299	6.625394999543102e-06
Mmd	4.504421	0.44603115	6.655400513930775e-06
Uqcrb	4.5015635	0.24423134	6.745538888865061e-06
Eif4a1	4.4839525	0.2224047	7.3272931664611825e-06
Emc10	4.482265	0.47499195	7.385487417679148e-06
Pex13	4.4817066	0.49713057	7.404846057744961e-06
Chordc1	4.468662	0.302931	7.87104178799241e-06
Arpc4	4.464984	0.22947802	8.00747088252935e-06
Ipmk	4.452602	0.67930907	8.483600812266994e-06
Prdm1	4.450017	0.90545005	8.58634231778191e-06

Serinc2	4.4430537	1.8414326	8.869105380123267e-06
Lsm6	4.4402103	0.31812236	8.987090397371785e-06
Tpst2	4.436001	0.21404627	9.164545118444666e-06
Pik3ap1	4.4347887	0.3455676	9.216257650395744e-06
Steap3	4.4293017	1.4871776	9.453866134577511e-06
Eif4e	4.4290876	0.40989852	9.463246122671017e-06
Anp32e	4.4282618	0.35023206	9.49954680514482e-06
Tacc1	4.4264054	0.25421384	9.581644388294511e-06
Nme1	4.421429	0.253789	9.805021552941306e-06
Nupr1	4.413527	1.4437716	1.0170011366283278e-05
Nckap1	4.404561	0.88756573	1.0599834230854592e-05
Rhbdf2	4.4024634	0.73831975	1.0702857440514702e-05
Bag1	4.398619	0.29625845	1.089417181647834e-05
Map4k5	4.3888006	1.0383648	1.1397738859650079e-05
Mib1	4.3843656	0.45688906	1.1632436418066334e-05
Morrbid	4.383938	0.59494555	1.165531535840993e-05
Zp1	4.3779187	2.990386	1.1981797707265505e-05
Rasgrp1	4.3741903	0.21977895	1.2188394724677871e-05
Lrrc49	4.3642383	1.8777196	1.2756649029862875e-05
Alyref	4.36275	0.32872728	1.2843775721721198e-05
Tmbim6	4.361021	0.19156328	1.2945701657091775e-05
Rab11a	4.356924	0.3645093	1.3190296114966112e-05
Coro2a	4.3539414	0.29121464	1.3371143282260417e-05
Wdr1	4.3534274	0.30095398	1.340254313721033e-05
Dync1li2	4.352584	0.68741494	1.3454242291591657e-05
Suz12	4.348808	0.31292692	1.3687954031093122e-05
Elob	4.339827	0.21521795	1.4259481964331062e-05
Srsf9	4.317998	0.34034935	1.574510555114857e-05
Rgmb	4.317362	1.4547207	1.5790509197813275e-05
Bet1	4.3025556	0.5162089	1.6883936331629246e-05
A330023F24Rik	4.298316	0.5714222	1.7210055969087335e-05
Fam111a	4.29181	0.41006798	1.772224802983541e-05
Rab1a	4.288281	0.31806132	1.8006137874464463e-05
Ncf4	4.2842793	0.5601035	1.8333263595387853e-05
Plekhm3	4.2840505	0.48546237	1.8352133483031865e-05
Anxa5	4.2797785	0.28390923	1.8707952471227064e-05
Il18rap	4.2696095	0.3887408	1.9581566899157884e-05
Slc35b1	4.2683673	0.5840208	1.969087245931433e-05
Nlrc3	4.2664485	0.40504104	1.986094288401996e-05
Ankib1	4.254363	0.28586107	2.096448262332354e-05
Klrc1	4.249943	0.66632575	2.1382530441724107e-05
Ahsa2	4.239221	0.6109348	2.2429678874457814e-05
B3gat3	4.234263	0.32129017	2.2930276335331457e-05
Ptprcap	4.2268505	0.1288971	2.3698494464118578e-05

Itgal	4.220273	0.16116281	2.4400642826129678e-05
Ube2d3	4.213859	0.1638384	2.5104377339214266e-05
Cyfip1	4.204421	0.43215528	2.6175133238129557e-05
Spg21	4.201052	0.40657672	2.656774168595772e-05
Eloc	4.1999145	0.27766687	2.670160269724449e-05
Nlrc5	4.1949263	0.3779658	2.7296029737730166e-05
Cstb	4.1912546	0.3223067	2.7741636681395855e-05
Atp8b4	4.171977	0.24536948	3.0196828068285162e-05
Mex3c	4.1700015	0.5052718	3.0459790308683612e-05
Leprotl1	4.1658363	0.23094329	3.1021333793654616e-05
Bsg	4.1627617	0.22138198	3.144217957228085e-05
Ptpn4	4.158457	0.25081858	3.204048608890247e-05
Ythdf2	4.154491	0.46742702	3.260128138232026e-05
Ifi204	4.150667	2.6938164	3.3150731794204254e-05
Ptp4a2	4.1480055	0.16944903	3.353843675732804e-05
Gem	4.1408963	1.5718515	3.459512624711601e-05
Cldnd1	4.1278543	0.44854864	3.661636152594804e-05
Ldha	4.1253147	0.19898364	3.70228483923585e-05
Gpd2	4.123069	0.43920758	3.7385833771778556e-05
Glipr2	4.109715	0.2886467	3.961480370174563e-05
Tec	4.107876	0.33820522	3.993145577074915e-05
Dync1i2	4.1072373	0.30876815	4.004200088290267e-05
Ostc	4.105181	0.23083895	4.0399776912919236e-05
Trdv4	4.103381	0.19624598	4.071557898792262e-05
Stap1	4.0861	0.39045405	4.3868491762049946e-05
Txndc11	4.0828648	0.5032249	4.4483920890053965e-05
Ncor1	4.0762815	0.09558351	4.5761631053636326e-05
Sh3glb1	4.0751348	0.21819212	4.598772167134506e-05
Bin1	4.073189	0.30143693	4.637379851826246e-05
Fkbp4	4.0560174	0.33994004	4.9916503328806695e-05
Tor1aip1	4.0530677	0.23906253	5.055039256434669e-05
Ube2n	4.047423	0.259329	5.178459691745755e-05
Myo1g	4.0448413	0.2780337	5.2358551388899385e-05
Ahsa1	4.033264	0.35724786	5.500737187141125e-05
Rnf11	4.028419	0.52531224	5.615323969653987e-05
Arhgef12	3.9957817	1.2558955	6.44811560709642e-05
Fth1	3.9905174	0.114048325	6.59293257389981e-05
Wdr95	3.972045	1.3017918	7.125828240972336e-05
Trak1	3.969891	0.55457073	7.190549631949526e-05
Ranbp1	3.9643655	0.24994713	7.35914607836249e-05
Vamp5	3.9600132	0.67851603	7.494563342316023e-05
Sit1	3.955664	0.5998645	7.632239875646977e-05
Rnf13	3.9531744	0.31354213	7.712119208615568e-05
Hsd17b11	3.951897	0.716742	7.75341364623795e-05

Tent5c	3.9478862	1.5314815	7.884421637714372e-05
G3bp1	3.9434004	0.21878828	8.033435136980747e-05
Plekha6	3.9384809	1.0102556	8.1999158017955e-05
Cct6a	3.9337602	0.27269998	8.362722243639626e-05
Cuta	3.9286683	0.3324285	8.54175858006425e-05
Trim37	3.9119754	0.7112574	9.15442774388034e-05
Xrn1	3.9103413	0.27146953	9.216577741718862e-05
Nedd4	3.9073586	0.3269065	9.331058758920654e-05
Ncoa1	3.9030867	0.3629934	9.497367839033142e-05
Cdk6	3.901521	0.290287	9.559014863627185e-05
Ctsd	3.8830576	0.25480154	0.00010315115665316528
Trp53i13	3.8808384	0.48019305	0.00010409702555782974
Dnah8	3.879543	0.37698796	0.0001046528758852243
Scamp2	3.8713198	0.38169745	0.00010824763054863273
Cpne8	3.8692017	1.7707781	0.00010919228621910486
Ttc7	3.8686461	0.34137467	0.00010944132742293371
Srpr	3.8676598	0.3035465	0.00010988479622024149
Mogs	3.8650336	0.52573127	0.0001110738798016821
Uap1	3.864199	0.68280387	0.0001114543931955021
Sdcbp2	3.8600695	0.76887727	0.00011335480241124917
Arl8a	3.8576958	0.5381326	0.00011446098739018014
Srsf3	3.8538277	0.16691464	0.00011628539700395558
Ccser2	3.8518107	0.41000715	0.00011724768259239454
Ubr5	3.8468761	0.29477808	0.00011963341790652803
Rasa4	3.83865	0.5251608	0.00012371264232894074
Eif4g1	3.8374379	0.29265317	0.00012432466734095594
Ptprj	3.829809	1.125355	0.00012824282767906456
Trabd	3.8271677	0.42175394	0.00012962613903162484
Nsd2	3.8263896	0.46158835	0.00013003649424924726
Anxa6	3.8235197	0.17206833	0.00013156008549558554
Plekhj1	3.820733	0.30662516	0.00013305559925542827
Cap1	3.810261	0.18412353	0.00013882016214074872
St3gal4	3.8049374	0.43154067	0.00014184010626870224
Gstm5	3.8034222	2.611287	0.00014271082232048581
Tmem154	3.8000772	0.38605243	0.00014465108497597895
Tmem37	3.7986393	0.71454746	0.00014549269825646176
Aldh9a1	3.798066	0.5388848	0.00014582958443415617
Msn	3.7844684	0.14050803	0.0001540374190147078
D16Ertd472e	3.7826028	0.2272371	0.00015519694525058144
Capza2	3.7770505	0.19003029	0.0001586966258629239
Herpud1	3.7758977	0.36383727	0.00015943240031769234
Arid3b	3.7736013	0.5433219	0.00016090783915735077
Creb3l2	3.772024	0.538597	0.00016192880243398136
Hif1a	3.7704968	0.3509904	0.000162922882871434

Agpat5	3.7697868	0.32434133	0.00016338706405626924
Gimap9	3.7693799	0.26574418	0.0001636537035823535
Ubash3b	3.76864	0.31708086	0.00016413937437783534
Ubtf	3.7669764	0.31740084	0.00016523660490722587
Frrs1	3.7657495	0.4978541	0.00016605022964354547
Son	3.765696	0.17584595	0.00016608577585698977
Tsg101	3.7634916	0.39507142	0.0001675573151825553
Cdc42bpb	3.751754	2.1037161	0.00017560170586590094
Ttyh2	3.737705	1.5565486	0.0001857075798333099
Eif3a	3.735866	0.1771282	0.0001870701382056469
Ptpn13	3.7235017	0.40484536	0.00019647853486962657
Ppp1ca	3.7207596	0.15978912	0.00019862440543267162
Hprt	3.7090487	0.24173416	0.00020803937068433133
Sh3pxd2b	3.706268	3.727809	0.00021033566020428832
Slc25a24	3.7061434	0.27681378	0.0002104392547863885
Psma3	3.7010782	0.18194155	0.0002146853862715017
Clock	3.7002285	0.3226111	0.00021540547225358037
Tceal3	3.6888206	3.136695	0.0002252960349273823
Ncoa7	3.685707	0.33479422	0.00022806847272356122
Comt	3.6744597	0.23039706	0.0002383532498026775
Mcur1	3.6718335	0.46924493	0.0002408165403538539
Or4c12	3.6717741	0.9429941	0.00024087254621724688
Commd7	3.6687438	0.2997326	0.00024374510324319694
Nfkbiz	3.6664386	0.65419996	0.0002459519905564907
Smyd2	3.6648996	0.45633286	0.00024743556141011296
Ccdc88c	3.6642492	0.198342	0.0002480653064482263
Trappc6b	3.6489198	0.2845011	0.00026334536640704236
Rbms1	3.6369948	0.19919808	0.00027583735638363774
Csf2	3.6320574	0.7327739	0.0002811704927567322
Ide	3.6283796	0.29472622	0.0002852056952138133
Ahnak	3.6266713	0.15330182	0.00028709827590892825
Ggh	3.6190631	0.35202566	0.00029567145917987653
Cmpk1	3.6107836	0.21794653	0.00030527335442841213
Pqlc3	3.6105666	0.59747213	0.000305528738304299
Casp7	3.6073375	0.7227656	0.0003093552558022717
Rbx1	3.6031456	0.13976412	0.00031438928143768114
Ier2	3.5980566	0.2512793	0.000320603814737473
Tm9sf3	3.5820024	0.1778532	0.00034097062872340717
Mtarc2	3.581744	0.28191584	0.00034130820655680033
Ppp1r18	3.5813043	0.17221023	0.00034188319536653254
Kpnb1	3.576768	0.4039931	0.0003478688233838792
Chd4	3.5741506	0.19240862	0.0003513666684523004
Ddx39a	3.563203	0.40636772	0.00036635688083622685
Map2k3	3.5611355	0.6732185	0.0003692544348985562

Sec61a1	3.5542283	0.3878167	0.00037908983859089707
Tax1bp3	3.5394337	0.70470583	0.00040098631646316214
Ptprs	3.539006	1.2724133	0.0004016367052642821
Cdk2ap2	3.5367334	0.12668787	0.0004051084519411338
Bnip2	3.536481	0.29208234	0.0004054959272665324
Ncbp3	3.5362015	0.39375946	0.0004059248325047324
Eif1ax	3.5359669	0.3044911	0.0004062856231209869
Nras	3.5324256	0.36895654	0.00041176593834300846
Cpsf2	3.5310442	0.42234302	0.0004139224734325304
Def6	3.5252452	0.22754237	0.0004230908802861718
Rnf6	3.5190897	0.31726396	0.00043303017524639047
Eif4ebp1	3.5117488	0.44786993	0.00044516837669601506
Ube2m	3.5104387	0.2818853	0.0004473678021013392
Tspan13	3.5066242	0.3989682	0.00045382946082238226
L1cam	3.4978724	0.43954796	0.000468985642623853
Tle3	3.4945657	0.38223323	0.0004748335631160516
Kmt5a	3.4807456	0.3658421	0.0005000201263859432
Kdelr2	3.4772668	0.37973696	0.0005065534597935941
Atosb	3.4734344	0.9512819	0.000513842804676396
Ufl1	3.4722104	0.45266867	0.0005161913977562297
Septin9	3.468108	0.23360299	0.0005241369238973828
Ctbp1	3.467089	0.31161797	0.000526127954229311
Vdr	3.4494364	1.8293773	0.000561758158552126
Cd69	3.448652	0.42940506	0.000563392168197478
Vasp	3.4464774	0.15852256	0.0005679460126078042
Tfg	3.4446118	0.37816283	0.0005718801570132021
Marveld1	3.4439225	0.49838796	0.0005733399431545791
Selenow	3.4410408	0.15215386	0.000579481020342657
Sirt2	3.4404438	0.34566045	0.0005807611889101389
Rnpep	3.4401913	0.38436887	0.0005813033455047215
Txnl4a	3.4396179	0.4174355	0.0005825361100153048
Carhsp1	3.4359667	0.7275064	0.0005904434755490659
Cry1	3.4341812	0.24339424	0.0005943465716919414
Tmem167	3.426882	0.17627633	0.0006105542814712333
Cst3	3.4243271	0.18431908	0.0006163238218413389
Arf4	3.4162495	0.23229419	0.0006349004246737874
Fam13b	3.4155483	0.2524039	0.0006365371371002557
Tgfb1	3.4118764	0.30003598	0.0006451733634132621
Rheb	3.407866	0.22220378	0.0006547305808551591
Tmem123	3.4004893	0.24991834	0.0006726533953469461
Fam89b	3.3986683	0.35933486	0.0006771478242937215
Eno1	3.3949666	0.21992098	0.0006863694891178863
Vdac2	3.3932228	0.19632241	0.0006907542143714977
Sptan1	3.386699	0.22273386	0.0007073896388620183

Thy1	3.3807545	0.19546865	0.0007228712833044139
Ctss	3.3792126	0.45372704	0.0007269378589533548
Cisd3	3.376625	0.5652238	0.0007338102891985032
Tusc3	3.3748038	0.31217825	0.0007386831583739962
B4galnt4	3.3731937	0.95856273	0.0007430166504428728
Gcnt1	3.3565483	0.6471318	0.0007892193705515897
Csde1	3.3565009	0.18325426	0.000789355044696638
Cct3	3.353013	0.24817333	0.0007993694114264077
Pheta2	3.347621	0.84971726	0.0008150838740535314
Ehd1	3.3471694	0.9155181	0.000816412829934034
Dpy19l1	3.3470507	0.35581517	0.0008167628891537897
Flot2	3.342027	0.65596336	0.0008316896635565302
F2r	3.3333256	0.2293229	0.000858144720803313
H2-M3	3.3240538	0.37558421	0.0008871912234031235
Pmaip1	3.3204648	0.9181801	0.0008986764756380967
Ift20	3.319547	0.26877454	0.0009016363959496571
Tmed7	3.3166802	0.34755573	0.0009109384313573894
Btbd10	3.3166177	0.3904623	0.0009111418442933422
Atp6v1g1	3.3165317	0.21053526	0.0009114228170740417
Ndfip1	3.315468	0.11597107	0.0009148979978945253
Tob2	3.3105187	0.40180603	0.0009312323263257749
S1pr3	3.3056526	3.3925943	0.0009475551825742919
Pon3	3.3046365	1.1777661	0.0009509965245432596
Slc15a2	3.2917492	7.2475815	0.0009956637274312634
Slk	3.2891052	0.26917127	0.0010050646641542353
Nipsnap3b	3.286387	0.47554022	0.0010148152817408935
Tmf1	3.2795718	0.30093428	0.001039647326275949
Rbbp7	3.2783003	0.30484155	0.001044342116065934
Slc25a12	3.2748363	0.41618094	0.0010572318290358058
Vps29	3.2733986	0.26545298	0.0010626253781643013
Gtf2h5	3.2676678	0.2519502	0.0010843754503195328
Usp6nl	3.258274	0.7057919	0.0011209205108058183
Cbx5	3.2575078	0.43361488	0.0011239520594319508
Coro7	3.251667	0.36381018	0.001147303082309443
Pigx	3.2355416	0.27143136	0.001214121918686969
Plekho1	3.230423	1.7661448	0.0012360723301065096
Tmem109	3.2298138	0.5699287	0.0012387082128594274
Rap2a	3.2284355	0.7856276	0.001244693490288982
Lrrc75b	3.2258925	0.8356976	0.0012558054260314643
Capn15	3.220854	0.50803643	0.0012780925881362025
Kars	3.2186584	0.6576533	0.0012879175386117038
Ptk2b	3.2143924	0.19289745	0.0013072086829851418
Snx2	3.2135518	0.1768833	0.0013110418038344382
Lamb3	3.212931	0.4053068	0.0013138792803402844

Pip4p2	3.2037897	0.44772714	0.0013563152116021562
Dusp2	3.202934	0.44085997	0.0013603511448395049
Gxylt1	3.2005901	0.37060562	0.0013714647341366742
Spon2	3.2004623	1.1157789	0.0013720728173447036
Hsd11b1	3.195311	0.20413	0.001396802325046525
Rassf1	3.1922274	0.26454663	0.001411801788714278
Eif1b	3.1897972	0.24630748	0.0014237266504998254
Tbc1d10c	3.1880028	0.19123976	0.0014325913250853406
Samd1	3.1825633	0.8519016	0.0014597760485215334
Pphln1	3.1821682	0.4335216	0.0014617690938837932
Efhd2	3.179334	0.302641	0.0014761387443904838
Eif1a	3.1782942	0.3920858	0.0014814431851067089
Spty2d1	3.1775634	0.40973526	0.001485181953771551
Sar1b	3.1755047	0.27395895	0.001495761132138268
Kras	3.1733034	0.29119724	0.001507149842805352
Anxa11	3.1727269	0.24942325	0.0015101446690196035
Utp11	3.1706653	0.2673953	0.0015209030592435418
Prelid3b	3.1705582	0.54468215	0.0015214630527622813
S100a6	3.1677449	0.12869768	0.0015362623776403127
Fdx1	3.1629858	0.52182037	0.0015615999476787106
Crmp1	3.1580126	0.3829857	0.0015884869645389794
Ndufs5	3.1571481	0.1831471	0.001593204125856942
Map2k2	3.1528196	0.26569495	0.0016170168645744764
Gimap4	3.1511827	0.23482406	0.0016261072795823064
E330020D12Rik	3.1503747	0.36699754	0.00163061206199805
Pde11a	3.1498399	0.6715571	0.0016335994799425403
Sae1	3.1496587	0.28518546	0.0016346130248407862
Sap30l	3.1463165	0.3642709	0.0016534095098838864
Ankrd10	3.143236	0.3102596	0.0016709116393015862
Septin10	3.1403244	2.268188	0.0016876082369444215
Cycs	3.1400065	0.2682855	0.00168944049596476
Stac2	3.1359484	2.5375056	0.0017129931180562947
Eea1	3.134264	0.42926228	0.0017228577445959898
Sfr1	3.1319942	0.21936111	0.0017362323926801502
Gnas	3.1313972	0.104696274	0.0017397669440316023
Psmd8	3.131326	0.19551563	0.0017401894218953178
Zfp362	3.130405	0.5328748	0.001745654912934972
Zcrb1	3.130176	0.21510226	0.0017470149152675693
Sel1l	3.1287918	0.3246717	0.0017552663887771297
Timm17a	3.1259458	0.32994035	0.0017723423803882885
Uhrf1bp1l	3.1255417	0.22612299	0.0017747788702557061
Lxn	3.1235127	0.37941483	0.0017870616922998003
Klf8	3.1216946	0.8308331	0.0017981340012390387
Ugp2	3.1208389	0.44524705	0.0018033662865927424

Tnfrsf1a	3.120643	0.72389996	0.0018045673211126267
Dynlt5	3.1186821	4.5040464	0.0018166181638193553
Lamc1	3.115952	0.43012878	0.001833521253775906
Batf	3.1138396	0.28288615	0.0018466976410900428
Oip5os1	3.1119058	0.19718035	0.0018588383367313197
Nans	3.1112134	0.42714968	0.0018632014128888966
Sh2b1	3.102821	0.5505285	0.0019168551570834902
Hnrnpa3	3.0997849	0.10906363	0.0019366124071106387
Hapstr1	3.0993214	0.28988227	0.0019396445865654394
Selenoi	3.0987303	0.5334046	0.0019435188858924822
Irf4	3.0984213	0.7766508	0.0019455464710516743
Cnih4	3.0946631	0.374205	0.001970364887062281
Dbi	3.091223	0.22109245	0.001993338487624985
Rcn2	3.082183	0.45608833	0.002054885485615825
Trim36	3.0820165	0.95605487	0.0020560342913176605
Grap2	3.0817552	0.13529107	0.002057840747926655
Vps37a	3.080047	0.32898927	0.0020696801852695406
Tpgs2	3.076078	0.48019522	0.0020974305052051502
Bscl2	3.0739834	0.36834955	0.0021122113633724906
Klf10	3.0606356	0.600486	0.0022086768032487415
Myo1f	3.053776	0.1364381	0.0022598068731224386
Ilk	3.0520382	0.40058842	0.002272932051782132
Rce1	3.0488474	0.5677394	0.0022972104867272597
Fbxl2	3.0361414	0.4183054	0.002396296070181843
Elk3	3.0360968	0.17668897	0.0023966237980099906
Macrod2	3.034463	0.75340736	0.002409643942298468
Gmeb1	3.0314178	0.47755772	0.0024340816638974147
Bub3	3.0305295	0.22876018	0.002441252939520515
St6galnac3	3.028857	0.11757437	0.0024548085480435673
Nup50	3.0179513	0.284808	0.002544898987709237
Raly	3.012975	0.1975815	0.0025870015372146645
Exoc6b	3.0116262	0.2240435	0.002598522410616009
Hmga2-ps1	3.0079038	0.65358764	0.0026305628225296953
Atp2b4	3.0069652	0.21577244	0.0026387000678015885
Mtfr1l	3.005658	0.3457327	0.0026500687352435065
Taok3	3.0046093	0.18106571	0.002659221854618388
Mlec	3.0001056	0.20600674	0.0026988604268960805
Pecam1	2.9947166	0.23787954	0.0027469999355671094
1810009A15Rik	2.9917755	0.29575822	0.002773601964272724
Blvra	2.9905486	0.5896811	0.0027847689705710406
Tamalin	2.9903584	0.6143913	0.0027865031210635733
Exoc2	2.9852784	0.19433974	0.002833204253656884
Cfdp1	2.9808042	0.23268245	0.002874924718994843
Slc6a6	2.9803467	0.3122061	0.0028792224099822776

Ppt2	2.9799101	0.39617696	0.002883330221499852
Mmgt2	2.9795654	0.38413525	0.0028865755355714294
Maged1	2.9784305	1.9150552	0.0028972862743191176
Sec24d	2.9749222	0.5003685	0.002930629688117593
Apol7e	2.9747646	0.4780298	0.0029321342260978742
Slc2a3	2.9673584	0.3461677	0.00300370573783585
Dubr	2.9651718	0.61212796	0.0030251382191451635
Tuba1c	2.9643579	0.7461885	0.003033152726084209
Stx11	2.9643164	0.47369725	0.003033562746017375
Rab14	2.9596462	0.16638793	0.003079924968152871
Sarnp	2.9573824	0.17176788	0.0031026299662820674
Ckap5	2.9556742	0.34709945	0.0031198639190100053
Cyth1	2.9551752	0.20064571	0.0031249156799491476
Kmt2c	2.952142	0.19376786	0.00315577786783406
Kmo	2.9515657	2.004271	0.00316167330240766
Smim15	2.9505913	0.27363977	0.0031716636801433096
Snx5	2.9499586	0.17762823	0.0031781667344075244
Acot11	2.9485056	0.6755133	0.0031931422816175465
Sec61g	2.9476857	0.1560377	0.003201623102605223
Memo1	2.9446912	0.3903386	0.0032327712088034265
Ahdc1	2.9439127	0.51425445	0.003240912330009026
Ciao2b	2.942178	0.25658685	0.0032591262169522853
Stab2	2.9409213	1.8645588	0.003272376993330757
Rangap1	2.9407668	0.32719576	0.003274009313461019
Strap	2.9395905	0.30023104	0.0032864644126541693
Stx1a	2.9375048	0.54305387	0.00330865003110861
Igtp	2.9361858	0.27206206	0.0033227522949363024
Ndufa11	2.9360611	0.2213289	0.003324089123024773
Tmem248	2.9280517	0.51432	0.003410932643849622
Trappc14	2.9250631	0.83438754	0.003443863542961066
Gbp7	2.9147842	0.309663	0.003559346207314317
Xpo1	2.9037356	0.3159384	0.0036873924912842904
Sertad1	2.901279	0.6764293	0.003716429543060452
Acpp	2.8999538	0.24613425	0.0037321753200453096
Capzb	2.8949273	0.15356939	0.00379246335926042
Ctdnep1	2.8944016	0.377692	0.003798820948678267
Smim3	2.887949	0.9008264	0.003877628585599397
Itga2	2.8847077	0.7964873	0.003917771583956995
Rasa2	2.884182	0.25498372	0.00392431971224965
Bcl10	2.8815467	0.259355	0.003957284305443074
Txnl1	2.8779194	0.21276058	0.0040030730551370115
Derl1	2.874928	0.26259786	0.00404119794997648
Mrpl4	2.8698537	0.3027892	0.004106616909356758
Plin3	2.8677177	0.22901274	0.00413444197788233

Hycc1	2.8635378	0.5409916	0.00418938789884569
Kri1	2.857819	0.5399483	0.00426563588237287
Acbd3	2.8562088	0.2554434	0.00428733003784151 9
Edf1	2.8509862	0.14245689	0.00435838624692150 1
Tmem126a	2.8505614	0.30971327	0.00436421282326026 9
Csnk2b	2.8494651	0.17721868	0.00437928046769055 4
ENSMUSG00000095041	2.8483036	0.20134503	0.00439529789613400 6
Ssna1	2.8463755	0.21150345	0.00442200162132349 3
Papola	2.8443167	0.14545596	0.00445067801983312 95
Wbp4	2.8435056	0.31029075	0.00446202097566338 2
Rell1	2.8429413	0.39381948	0.00446993079334921 2
Ccdc69	2.8416073	0.5325291	0.00448867345382291 5
Itpk1	2.8410637	0.32937708	0.00449633284785399 1
Fosl2	2.838987	1.1049799	0.00452569834111719 6
Pja2	2.8324602	0.51378375	0.00461913059245697 6
Eif2s1	2.8285239	0.19086161	0.00467632006979349 85
Ranbp2	2.8279564	0.22894722	0.00468461664275717 9
Kif5c	2.825883	0.8621234	0.00471504951191600 1
Rassf3	2.8219407	0.26306188	0.00477340083966916 1
Lrrcc1	2.8209484	0.39584643	0.00478819018625082 4
Nars	2.8201878	0.28157744	0.00479955379144271 3
Spacdr	2.819377	0.14043868	0.00481169889542175 5
Clcn5	2.8160615	0.5195894	0.00486163674217175 4
Tmem33	2.8109398	0.26241663	0.00493970297568220 1
Espn	-2.8189936	-1.190453	0.00481744746000870 2
Mitd1	-2.8195016	-0.31136215	0.00480982860963332 5
Zc3h6	-2.823637	-1.084366	0.00474821297983546 36
Klhl42	-2.824457	-0.9608764	0.00473608129147104 74
Phlpp1	-2.8275614	-1.4960607	0.00469040170555780 34
Cct4	-2.8331199	-0.18344328	0.00460961084527226 4
Slfn1	-2.8333724	-2.2918687	0.00460597060331630 6
Jmjd1c	-2.8352232	-0.18721604	0.00457936914008066 2
Serbp1	-2.8391268	-0.096899725	0.00452371840233779 7
Ptbp2	-2.8435175	-0.32951525	0.00446185458958853 55
Srsf7	-2.8451397	-0.11170942	0.00443919557983945 2
Bcl6	-2.8468063	-3.0814214	0.00441602273442427 4
Clta	-2.8541472	-0.1093439	0.00431525421861456 5
Zfp182	-2.8563812	-0.24083036	0.00428500375212807 15
Cd3e	-2.85896	-0.077582486	0.00425032615591711 8
Borcs8	-2.8621564	-0.36197102	0.00420769211990954 2
Aven	-2.8634903	-0.27003857	0.00419001652059831 2
Myc	-2.8650975	-2.5086246	0.00416880868420474 8
Creb1	-2.867486	-0.17333494	0.00413747081580843 46
Surf1	-2.8691437	-0.46392688	0.00411584721955029 2

Ugcg	-2.869215	-0.5163196	0.00411491947686319
Xkr6	-2.8714015	-0.5335988	0.004086560682176136
Atp11b	-2.8739624	-0.24167669	0.004053572581569152
Mrpl34	-2.875433	-0.26205415	0.004034738734841711
Slc38a1	-2.8808963	-0.17228235	0.003965461863554837
Trgv1	-2.8809052	-0.65726864	0.003965349738627276
Mtdh	-2.887221	-0.13939013	0.0038866106102990908
Hacd3	-2.8921318	-0.4278236	0.003826374017278464
Zmym4	-2.8950937	-0.35854325	0.003790453932944743
Rhoh	-2.9055955	-0.26007196	0.0036655501407887895
Atp5j2	-2.9074938	-0.11359189	0.0036433755921520734
Txlng	-2.9082572	-0.33570057	0.003634491639244811
Bod1l	-2.9110053	-0.21225843	0.0036026791358762774
Ccdc171	-2.9254434	-0.63629913	0.0034396575203380756
Rnf213	-2.925907	-0.45079497	0.0034345377524523553
Vps13a	-2.9269733	-0.2454411	0.003422782062253484
Abtb3	-2.9299204	-2.702331	0.003390488568675964
Bicra	-2.938096	-0.30706915	0.0033023471321111967
Atp5c1	-2.947151	-0.11782862	0.0032071651265272814
Lipa	-2.9500089	-0.59651077	0.0031776472653850143
Sf3b6	-2.951013	-0.12833302	0.0031673350509692534
Ifnar2	-2.9538293	-0.30440053	0.0031385745794992215
Relch	-2.9559445	-0.2766837	0.0031171306583642005
Bpgm	-2.9592273	-0.42642334	0.0030841148283134107
Serp2	-2.9635024	-1.073334	0.003041597605625506
Ubash3a	-2.9640074	-3.2476463	0.003036610191407224
Tnfrsf18	-2.9654036	-1.2783864	0.0030228602518851035
Cenpa	-2.9735198	-0.48975238	0.0029440534292552636
Cpt1a	-2.9740813	-0.42088974	0.0029386715235274964
Ndufa2	-2.98226	-0.17444563	0.002861289178648454
Ubr2	-2.9872272	-0.2124977	0.0028152046484373867
Ewsr1	-2.991107	-0.20829608	0.0027796806024461035
Cdca7l	-2.9919686	-0.8829734	0.00277184817505626
Khnyn	-2.9995382	-0.4986649	0.0027038925822291246
Txnip	-3.0006404	-0.14113303	0.002694125915676993
Ccr9	-3.0058482	-2.307945	0.002648412333212358
Ndufc2	-3.005857	-0.22371803	0.002648334712591233
Hdac7	-3.0143862	-0.5390228	0.002574997771684479
Nsd1	-3.0172799	-0.1665699	0.002550542880390254
Gpr18	-3.018477	-0.5399779	0.0025404867493729723
2010004M13Rik	-3.019282	-0.96787095	0.002533744843785265
C1galt1	-3.019603	-0.35305923	0.0025310625960577233
Fbxl12	-3.020616	-0.40507093	0.0025226106840781255
Ly6c2	-3.0230193	-1.9114965	0.0025026622570973327

Tmem106b	-3.0232658	-0.34343922	0.0025006238160397943
Prmt7	-3.0256426	-0.70697105	0.00248105392323257
Tcf20	-3.028239	-0.24715589	0.002459834058767267
Cotl1	-3.0290084	-0.1260081	0.002453577766581353
Wdr37	-3.0296144	-0.32147273	0.002448660285525871
Bicdl1	-3.0326478	-1.8436061	0.0024241840316602896
Selenot	-3.035372	-0.1664971	0.002402392039088888
Chp1	-3.036266	-0.36099946	0.0023952781271443005
Cd9	-3.0377545	-2.1269183	0.0023834801113716037
Cct2	-3.0398993	-0.16085522	0.002366571341756991
Shld1	-3.0457342	-0.530973	0.0023211299385031818
Letm2	-3.045936	-0.91283447	0.0023195710199008517
Slc22a15	-3.047671	-0.530876	0.0023062220879725005
Tmem234	-3.0498545	-0.18650016	0.0022895216465464684
Fbxo8	-3.0516133	-0.41777357	0.002276151033142907
Mrps9	-3.0518212	-0.3211472	0.0022745747865563362
4930453N24Rik	-3.0522313	-0.24080421	0.0022714702575579587
Ylpm1	-3.053277	-0.3557822	0.002263569029856688
Tpd52	-3.0540226	-0.9305325	0.002257950303635962
Atg16l2	-3.0628045	-0.4932543	0.0021927337698707137
Wdr83os	-3.0636957	-0.24096902	0.002186212474427682
Itgae	-3.0658257	-0.36637172	0.002170698532926841
Mfsd4a	-3.0664911	-0.5469414	0.0021658725083117214
Rcc2	-3.0678399	-0.32579118	0.002156121348279675
Rnf2	-3.0678785	-0.42271283	0.0021558427237291116
B3gnt5	-3.0708344	-0.51036465	0.0021346149210999246
Tspan5	-3.070977	-1.4844693	0.002133595728738555
Tut7	-3.071978	-0.23716417	0.0021264527032488086
Hexb	-3.0733833	-0.30267686	0.0021164640179998514
Lsp1	-3.0794706	-0.052541513	0.0020736887846077912
Irf9	-3.0914042	-0.54349893	0.001992122198740976
Eps8	-3.0924113	-4.132072	0.001985375225434698
Fam117b	-3.0935552	-0.35922474	0.001977738173715012
Numa1	-3.096303	-0.22126877	0.0019594995083437577
B230217O12Rik	-3.0976577	-0.9226408	0.0019505652781372405
Tbl1x	-3.0983174	-0.19873628	0.0019462292680965869
Tmem260	-3.099063	-0.466253	0.0019413375024832154
Tbc1d4	-3.1005306	-1.6534938	0.0019317428418485975
Tex10	-3.1032548	-0.4657829	0.0019140478518892034
Rtn4rl1	-3.1044462	-1.1602961	0.001906356791441705
Inpp4b	-3.1147666	-0.5087104	0.001840904922087892
Cxcr4	-3.1236908	-0.5780302	0.0017859795518913922
Acss2	-3.1268785	-0.9246998	0.0017667286962233797
Mbtd1	-3.1278322	-0.30000973	0.0017610067671414505

Abca2	-3.1294692	-0.6659519	0.0017512247205193543
Ryk	-3.1301732	-1.2689806	0.001747032584042007
Rinl	-3.1303039	-0.1504004	0.0017462553133797104
Fas	-3.1416614	-1.6781648	0.0016799224667025207
Gpr171	-3.1435478	-0.48066494	0.0016691317612232807
Cd3g	-3.1446648	-0.075570285	0.0016627723999285019
Ppil1	-3.1497478	-0.345223	0.001634114487840384
Mppe1	-3.1508203	-0.27897078	0.001628126388583788
Pdss2	-3.1536336	-0.4385103	0.0016125138380999574
Appl2	-3.1556003	-0.665134	0.0016016818528162863
Ncoa2	-3.1556418	-0.19371644	0.0016014535025249137
Prrc2b	-3.1609387	-0.23435043	0.0015726151967742337
Gbe1	-3.1609745	-0.56047666	0.0015724227385916219
Entpd5	-3.1646404	-0.4553796	0.0015527470031567513
Eml5	-3.1674597	-0.97757673	0.0015377700038854574
Bola2	-3.1712534	-0.35581195	0.0015178264871030026
Mtmr3	-3.1735053	-0.2641783	0.0015061014055715637
Ddx39b	-3.1769605	-0.21738139	0.0014882737430655564
Kdm3a	-3.1795657	-0.43515432	0.0014749589991879817
Mrps26	-3.1806324	-0.24817055	0.0014695403399989895
Slc16a10	-3.1865768	-0.86292166	0.001439672352922979
Rhobtb1	-3.1878335	-0.3679294	0.001433430514044024
Zkscan1	-3.1901567	-0.4896122	0.0014219568715669987
Bcas3	-3.1906707	-0.2664814	0.0014194300491307218
Zc3h13	-3.1909854	-0.19027811	0.0014178838685986596
Phtf2	-3.1912203	-0.5056778	0.0014167325365400285
Oard1	-3.196033	-0.39765522	0.0013933121828821
2310001H17Rik	-3.196731	-0.38861164	0.0013899445931346796
Hp1bp3	-3.2128832	-0.20814903	0.0013140967367817607
Jarid2	-3.2192764	-0.24585052	0.001285145167895699
Zfpm1	-3.2197905	-1.4574307	0.0012828434991947275
Rcsd1	-3.228563	-0.20684123	0.0012441376995002407
Prrc2c	-3.2294395	-0.099033035	0.0012403308910649289
Antxr2	-3.2313528	-0.2289829	0.0012320577776571509
Mpc1	-3.2357287	-0.2437003	0.0012133261862734429
Slc12a6	-3.2358832	-0.19442436	0.001212669754197895
Exoc4	-3.2411268	-0.18406326	0.0011905825143495702
Arpc3	-3.2450926	-0.09519683	0.0011741238351942493
1700025G04Rik	-3.2454255	-2.2321877	0.0011727526354474234
Rab11fip4	-3.250538	-0.8064563	0.0011518678972212242
Uqcrfs1	-3.251275	-0.2678938	0.0011488868561156907
Rab11fip1	-3.254816	-0.38834143	0.001134657878063354
Rnf144a	-3.2550569	-0.22844127	0.0011336969119231882
Atpif1	-3.2566788	-0.32948032	0.001127238891472254

Pglyrp2	-3.267445	-0.47164315	0.0010852293564868726
Nlk	-3.2706475	-0.327059	0.001073015446193449
Frat2	-3.2708912	-1.6333088	0.0010720915969646978
Rasgef1b	-3.2739658	-1.119399	0.0010604938987002718
Vps13b	-3.2847648	-0.21744645	0.0010206753204207825
Ube2c	-3.2888794	-1.8597229	0.0010058712368620744
Ifi47	-3.2958577	-0.3405814	0.000981216723565996
Ethe1	-3.2964995	-0.519635	0.0009789779633256816
Gpr146	-3.3050702	-1.1636652	0.0009495259998801935
Lsm7	-3.310471	-0.30532992	0.0009313905003960222
4833420G17Rik	-3.3105423	-0.32090572	0.0009311532486238901
Tmem108	-3.3116624	-2.4774885	0.0009274337590889409
Rab8a	-3.3129845	-0.18791278	0.0009230610963870652
Aak1	-3.3134658	-0.17023745	0.0009214739992675343
Mrpl35	-3.3187299	-0.47947082	0.000904278225626263
Tlk2	-3.3230376	-0.29399264	0.0008904289576000533
Bmt2	-3.32474	-0.33181918	0.0008850105115650371
Rnf114	-3.3344605	-0.38023403	0.0008546507443177611
Mtss1	-3.335928	-0.35824996	0.0008501519168011994
Mturn	-3.336635	-0.48539826	0.000847992314083923
Pcmtd2	-3.339178	-0.38921285	0.0008402669911242929
Lbh	-3.3450987	-0.26070282	0.0008225325773826498
Spock2	-3.35342	-0.83163714	0.0007981947371673648
Sidt2	-3.3580456	-0.4547728	0.0007849566979376919
Sik1	-3.3590913	-1.6928948	0.0007819922787243659
Slc25a36	-3.3607016	-0.48947388	0.0007774480604574531
Car5b	-3.3657608	-0.56238186	0.0007633288489358344
Uqcrc2	-3.368375	-0.25854704	0.0007561266082072247
Serpinb6b	-3.372876	-0.901775	0.0007438749411728705
Adamts6	-3.3757842	-0.64279693	0.0007360561949951358
Ext1	-3.3770409	-0.42797208	0.0007327015952974303
Ubr1	-3.387513	-0.33100364	0.0007052938595416806
Tbcel	-3.388535	-0.53852254	0.0007026708312583969
Mctp2	-3.3915741	-3.178877	0.0006949238396786412
Stat1	-3.402863	-0.34425002	0.0006668368690789032
Sdc1	-3.405828	-1.4548055	0.0006596373558896012
Bcl2l11	-3.4065232	-1.2496222	0.0006579597865713129
Mef2d	-3.4091375	-0.43437698	0.0006516864283018691
Scai	-3.4111369	-0.3436212	0.0006469263217469998
Slmap	-3.4123518	-0.30259323	0.000644049296995783
Kmt5b	-3.4167426	-0.28537562	0.0006337515239796936
Zfp827	-3.4227347	-1.8009913	0.0006199453412145523
Prrg1	-3.426986	-1.2454053	0.0006103205423896345
Gimap1os	-3.4362252	-0.9590997	0.0005898804493488554

Fars2	-3.4373481	-0.32071316	0.0005874399957804183
Fstl4	-3.4383879	-3.7073069	0.0005851887004339647
Ppp2r5a	-3.4462843	-0.20039162	0.0005683520366832418
Riok3	-3.4502652	-0.26191747	0.0005600361052125926
Slc25a27	-3.453548	-1.47202	0.0005532639695011557
Psap	-3.4605768	-0.17881505	0.0005390193557080169
Slc7a6os	-3.4697032	-0.60784805	0.000521033869037187
Bbx	-3.4790878	-0.33896324	0.0005031235017143381
Scaf8	-3.4831905	-0.25473458	0.0004954754865496963
Tmem229b	-3.4842958	-0.5076703	0.0004934339521671514
Nrip1	-3.4918356	-0.2631958	0.00047971336424723865
Dkkl1	-3.4945865	-1.0741743	0.0004747965721206219
Ccl5	-3.4958818	-0.84596527	0.0004724978523325617
Sumo1	-3.5004332	-0.18511812	0.0004645027704039785
Ndufs4	-3.5011282	-0.2035066	0.00046329275992822725
Mbnl2	-3.502774	-0.24101281	0.0004604397478692445
Ube2q2	-3.5031602	-0.45643598	0.00045977264744719796
Ramac	-3.5091138	-0.27219036	0.0004496024762367433
Resf1	-3.5203524	-0.24029166	0.000430973867052673
Adrb1	-3.5249126	-1.894101	0.0004236226465588987
Tbc1d15	-3.5277972	-0.36785415	0.00041903309823559964
Hipk2	-3.533415	-1.2475526	0.0004102280344131391
Lsm4	-3.5367186	-0.23727441	0.0004051312350295933
Senp7	-3.5478323	-0.27148083	0.00038841549670140426
Cited2	-3.5525262	-0.2825961	0.00038155112116518183
Uimc1	-3.5567656	-0.31902835	0.0003754490672775275
Lpar6	-3.5621128	-0.32153413	0.000367882097841582
Chaserr	-3.5621693	-0.22461733	0.0003678029901582252
Gpi1	-3.575244	-0.25227463	0.0003499016118661398
Ly75	-3.5765955	-0.91171175	0.00034809809621735556
Tsc22d3	-3.5774481	-0.47341123	0.00034696497010356416
Mrps35	-3.5784166	-0.570569	0.0003456820509362893
Gtf2i	-3.5789604	-0.40613678	0.0003449638306224589
Scmh1	-3.5872667	-2.2517018	0.0003341625099988283
Tra2a	-3.5948422	-0.26388645	0.000324588218969826
Ndufa7	-3.598027	-0.21268232	0.00032064042865764786
Zc3h12d	-3.6086714	-2.577637	0.00030776925363428556
Ubxn7	-3.6136148	-0.3903501	0.00030195765667306775
Cysltr1	-3.6149218	-0.9116478	0.0003004382067549252
Rcbtb2	-3.6200228	-0.3090646	0.0002945771233467398
Snx4	-3.6213775	-0.34739992	0.0002930386360774333
Cep97	-3.6229699	-0.51395893	0.0002912398624831085
Lsm10	-3.6312196	-0.48673356	0.0002820849253805507
Nsg2	-3.639627	-2.20295	0.0002730331866874178

Sos2	-3.6404352	-0.8379318	0.0002721776876523975
H2az2	-3.643715	-0.23029673	0.0002687310952946268
Snx14	-3.6489732	-0.35909027	0.0002632905624533541
Cab39	-3.6490178	-0.2016118	0.00026324490066004474
Icam2	-3.6518164	-0.47673994	0.000260392167411059
Ass1	-3.6522233	-0.5105108	0.0002599797019044968
Rgs2	-3.6527462	-1.007886	0.0002594507181808747
Mpped2	-3.654787	-2.907714	0.000257395521114724
Use1	-3.655803	-0.28636968	0.00025637811058347275
Zhx2	-3.657541	-0.6226538	0.0002546465403038924
Herc3	-3.6678853	-0.41523898	0.00024456481829194344
Dhx40	-3.6687944	-0.56470144	0.00024369696506532722
Ssbp2	-3.6727781	-2.9688435	0.0002399276869263552З
Itga6	-3.675544	-1.0726964	0.00023734307942356497
Zfp169	-3.677392	-0.83470684	0.00023563088822025195
Ms4a6c	-3.6808648	-2.650793	0.0002324442767961036
Cab39l	-3.6832175	-0.25296065	0.00023030837047823569
Gnl3	-3.684706	-0.70577526	0.00022896677390634395
Tnks2	-3.6876085	-0.2607081	0.00022637159898037052
Eif2a	-3.6927361	-0.40344936	0.00022185422691265762
Orai2	-3.7017345	-0.26977015	0.0002141305050239459
H2-Ab1	-3.7043936	-2.2698271	0.00021189710264193746
Epc2	-3.7070465	-0.3503505	0.00020969050781722708
Zfp395	-3.710775	-1.140923	0.0002066258689514224 5
Imp3	-3.7128723	-0.2858781	0.00020492039805764068
Otulinl	-3.716342	-0.24091797	0.00020212788479209292
Ikbke	-3.722967	-0.78511184	0.00019689529707139447
Camk1d	-3.730602	-0.09465734	0.00019102284771180193
Cd5	-3.7346392	-0.7262022	0.00018798446471892654
Snrpd2	-3.7393866	-0.20114861	0.00018446985664007527
Klf13	-3.7492437	-0.2243709	0.000177368664278271
Dnajc8	-3.7596	-0.22370812	0.00017018530128893396
Cnpy2	-3.7631977	-0.34913698	0.0001677545774821395
Pts	-3.767948	-0.432913	0.0001645950652895093
Plxdc2	-3.771394	-1.9230542	0.0001623381187374891
Sgk1	-3.7718039	-0.40064952	0.00016207156654230137
Pdcd2	-3.7869134	-0.67977333	0.00015253019148775727
Il17ra	-3.7953594	-0.33811694	0.00014742969698076707
5031425E22Rik	-3.7970676	-0.35530135	0.00014641783372107726
Nsmaf	-3.7992513	-0.33658943	0.00014513392888478708
Jmy	-3.8046253	-0.693724	0.00014201896129780402
Rasa1	-3.8105936	-0.29321715	0.00013863344098709424
Zfp800	-3.8154986	-0.31748834	0.0001359082728716626
Foxk1	-3.8322687	-0.4052786	0.00012696695806353728

Actn1	-3.8334422	-2.3173344	0.00012636252439400558
Ptger2	-3.8336322	-3.1689956	0.00012626484652431547
Psme2	-3.8376489	-0.22100082	0.0001242179582707245
Igbp1	-3.8431566	-0.36497745	0.00012146187116472881
1110038B12Rik	-3.8470216	-0.8831317	0.00011956238749610113
Sp4	-3.8474555	-0.348882	0.00011935098155049063
Trim25	-3.8483021	-0.5510797	0.00011893932070331241
Rigi	-3.8512402	-0.51961684	0.00011752114584147837
Hadh	-3.867761	-0.6027156	0.0001098393029423147
Ago2	-3.882597	-0.2586052	0.0001033467518388428
Acat1	-3.8827279	-0.30192405	0.00010329119249367185
Abcg3	-3.8856065	-0.8758887	0.00010207474725360985
Arl4c	-3.8917441	-0.39147252	9.95261720054628e-05
Rfx7	-3.892359	-0.24984027	9.927415870642556e-05
Pdgfb	-3.9083688	-4.5189114	9.29214073481415e-05
Agtrap	-3.9215739	-0.6411901	8.797243791255904e-05
Axin2	-3.926654	-0.55784327	8.613574012919895e-05
Hmg20a	-3.9293485	-0.41471913	8.517630423804284e-05
Appl1	-3.9306052	-0.31437135	8.473231080653856e-05
Elk4	-3.931244	-0.36528146	8.450747956554423e-05
Tfdp2	-3.935305	-0.67665625	8.309110532843831e-05
Cdk8	-3.935813	-0.19156682	8.291551614088409e-05
Klf12	-3.9380026	-1.2121162	8.216274202026574e-05
Cox6b1	-3.93824	-0.14259121	8.208141952835699e-05
Dhcr24	-3.9406734	-0.9740327	8.125324504048079e-05
Mei4	-3.9433944	-1.413995	8.033634259700492e-05
Dennd6a	-3.9471169	-0.5509142	7.909793848535855e-05
Zfp217	-3.9569888	-0.7335268	7.590046155491405e-05
Trac	-3.9722202	-0.52686733	7.120585577672969e-05
Prdx6	-3.9750423	-0.22600383	7.036670556943461e-05
Slc20a1	-3.9785926	-0.54701227	6.932442518002984e-05
Thap3	-3.9811385	-0.41553667	6.858596084851023e-05
Tcf12	-3.9879565	-0.21889164	6.664487426916286e-05
Slc35f2	-3.9954014	-0.97834194	6.458474707140863e-05
Prxl2a	-4.0034194	-2.7013774	6.243344066448467e-05
Eif4ebp2	-4.00694	-0.251977	6.151049609445454e-05
Mtif2	-4.0072103	-0.35000768	6.144015685526503e-05
Snhg1	-4.0100236	-0.5484086	6.0712670793533544e-05
Atp5d	-4.0107546	-0.1935599	6.052503224916469e-05
Arid4b	-4.0109563	-0.24107043	6.047326163380238e-05
Pnpla7	-4.0121746	-0.48622876	6.0162002736475445e-05
Stmn1	-4.0148425	-1.258921	5.948556154448759e-05
Pde4dip	-4.0183387	-0.6040715	5.860985803882944e-05
Cdk17	-4.0198836	-0.2115923	5.8226872138909703e-05

Phf14	-4.0239954	-0.27512422	5.721905621310103e-05
Acyp1	-4.0278187	-0.5329687	5.629671934286108e-05
R3hdm1	-4.0345445	-0.2850008	5.470829147279422e-05
Tjp3	-4.037355	-1.6199217	5.405723403482926e-05
Fam169b	-4.048784	-2.5881603	5.1484502944213976e-05
Slc25a33	-4.049678	-1.3529831	5.1288177928086165e-05
Scaper	-4.049746	-0.44847062	5.12732055643511e-05
Mrpl24	-4.0506463	-0.33435214	5.107634729236892e-05
Acsbg1	-4.05089	-0.24166366	5.1023195405176085e-05
Plcxd2	-4.061861	-1.0120252	4.868303139528834e-05
Acsl4	-4.06455	-0.3266613	4.8125278436872135e-05
Ndufa6	-4.0651674	-0.21920307	4.7997946674332195e-05
Cd28	-4.067954	-2.1835756	4.742768707188454e-05
Suclg1	-4.073438	-0.37286964	4.632411512316313e-05
Mbd5	-4.0750012	-0.30035907	4.601414822311779e-05
Pgls	-4.076359	-0.3622357	4.574644010540578e-05
Susd6	-4.07826	-0.37716562	4.537401326069815e-05
Hbp1	-4.0791245	-0.5457588	4.5205628037063915e-05
Rab21	-4.0792017	-0.2496604	4.519061218440262e-05
Clip1	-4.089849	-0.34032083	4.316539801049991e-05
Tsc22d1	-4.122608	-3.2300284	3.746067156502645e-05
Notch2	-4.127388	-0.43305206	3.669068395456608e-05
Large1	-4.1285973	-0.69918925	3.6498308660320266e-05
Septin8	-4.1340723	-0.40196058	3.5639108402704573e-05
Dkk3	-4.1443157	-2.6460674	3.408298648069435e-05
Rad50	-4.146716	-0.3330043	3.372777603181576e-05
Ptma	-4.1512733	-0.11103072	3.3063056637580746e-05
Sun2	-4.154051	-0.4731369	3.266402298636986e-05
Arl6ip5	-4.157438	-0.16953821	3.218368922208399e-05
Rprd2	-4.158353	-0.36898643	3.205507087111855e-05
Nop10	-4.162093	-0.25884753	3.153438289602764e-05
Pde4b	-4.1768756	-0.27412614	2.9554037483959843e-05
Bin2	-4.1787267	-0.21637587	2.9314590165197854e-05
Gramd4	-4.1859903	-0.84137684	2.8392565736030734e-05
Prkcz	-4.190898	-0.6670304	2.778526593400734e-05
Wdr26	-4.1929	-0.21069402	2.754105809057838e-05
Pdk1	-4.1938777	-1.7708384	2.7422595316123674e-05
Slc38a2	-4.2190166	-0.39891863	2.453702588984044e-05
N4bp2l1	-4.2207127	-0.659951	2.4353095412932298e-05
AI506816	-4.227944	-0.9507034	2.3583666386297768e-05
Traf3ip3	-4.229628	-0.27189124	2.3407779451679735e-05
Gimap5	-4.245638	-0.3951815	2.1797239339479694e-05
Tmem30a	-4.246125	-0.28787795	2.174992055161641 2e-05
Ifi27l2a	-4.2494407	-1.4967935	2.1430509058552357e-05

H1f2	-4.249886	-0.5614606	2.138791938125006e-05
Malt1	-4.2577233	-0.58686316	2.0651949749356945e-05
Rundc3b	-4.258534	-3.0626266	2.0577178141823073e-05
Tshz1	-4.259256	-0.41424513	2.0510840082654786e-05
H1f0	-4.2673483	-0.84049386	1.978099955965943e-05
Lpcat3	-4.2755866	-0.5291527	1.90634714899697e-05
Vars	-4.2760115	-0.5434692	1.902714990903805e-05
Ddx10	-4.288171	-0.36850995	1.8015048756764508e-05
Heg1	-4.28889	-0.6213516	1.795684280592504e-05
Pnrc1	-4.298726	-0.28252476	1.7178257837822013e-05
Chd6	-4.3012514	-0.403103	1.6983630775141422e-05
Samd9l	-4.3071337	-0.32929996	1.6538380298800108e-05
Klrd1	-4.3096113	-1.3685789	1.635418481795318e-05
Id3	-4.316034	-1.5091798	1.588575060861313e-05
Zmynd8	-4.3171988	-0.36235777	1.580219850449664e-05
Prkd3	-4.3215003	-0.5085082	1.5497184360719302e-05
Cers4	-4.3304157	-0.3801842	1.4882813344507922e-05
Aff4	-4.361562	-0.3140749	1.291374526833536e-05
Mapk14	-4.3684688	-0.40517873	1.2512076224669183e-05
Oxct1	-4.368513	-0.6802685	1.2509523938875638e-05
Ldlrad4	-4.3704057	-0.29294032	1.2401594223789781e-05
Prxl2c	-4.3765373	-0.45740482	1.2057952785414249e-05
Gpbp1l1	-4.385209	-0.33739024	1.1587439645842094e-05
Slc11a2	-4.38717	-1.0231123	1.148351050546559e-05
Cir1	-4.397624	-0.49881914	1.09442301837217e-05
Acp5	-4.3977995	-0.58988374	1.0935398030263186e-05
Ndufb5	-4.4000897	-0.27355015	1.0820604955271397e-05
Pfdn1	-4.403845	-0.3755036	1.0634895294708717e-05
Lcn4	-4.407882	-2.1168706	1.043862446831338e-05
Usp53	-4.409519	-0.66136783	1.0360037062676381e-05
Cox16	-4.410749	-0.23747328	1.0301361624401033e-05
Tanc2	-4.412107	-1.043057	1.0236960423212285e-05
2610035D17Rik	-4.4153867	-0.67152417	1.0082965525021643e-05
Snrpe	-4.418717	-0.19353868	9.928864565064899e-06
Lpp	-4.422766	-0.5107093	9.744525918828845e-06
Stag2	-4.42399	-0.2571309	9.68945158478317e-06
Coro1a	-4.4297624	-0.10892283	9.433703238221285e-06
C1qbp	-4.4355493	-0.45281968	9.183778085189649e-06
Nkg7	-4.4406385	-0.66279364	8.969241807433141e-06
Lars2	-4.4654326	-0.22767149	7.990710249707886e-06
Gatad2b	-4.4658246	-0.34270632	7.9760860484092e-06
ENSMUSG00000121501	-4.4710684	-0.32325485	7.782984503352117e-06
Tax1bp1	-4.4787064	-0.16651443	7.5096806236108725e-06
Snrpf	-4.492214	-0.18300255	7.048639657601603e-06

Scand1	-4.4926987	-0.27909124	7.0326256605239734e-06
Stk17b	-4.498055	-0.22236595	6.857794732963541e-06
Rac2	-4.503768	-0.1346772	6.675912085806796e-06
Ssr4	-4.5060644	-0.22717175	6.604107776930415e-06
Aplp2	-4.521358	-0.55187374	6.144417067996401e-06
Kdm5a	-4.5335236	-0.3239655	5.800782691540708e-06
Trim12c	-4.539851	-0.5678277	5.62939303444866e-06
Tnfaip8l2	-4.5436897	-0.3837632	5.5278060545074295e-06
Ccnd3	-4.5469546	-0.2215807	5.442777723896829e-06
A630014C17Rik	-4.548422	-3.5637293	5.404966765650098e-06
Lats2	-4.5511165	-0.3544817	5.3361983907820724e-06
Slamf6	-4.5535703	-2.6041124	5.274300880539335e-06
Tespa1	-4.5691223	-0.28145906	4.897703519563434e-06
Cnot6	-4.589312	-0.34262952	4.447090621142889e-06
Dph5	-4.5952153	-0.71369493	4.323030832332039e-06
Mif	-4.5974107	-0.27013788	4.277742385306894e-06
Prdx2	-4.5974345	-0.35942414	4.277254614241659e-06
Ifi209	-4.6069202	-0.34953216	4.0867702307375525e-06
Sik3	-4.61009	-0.29868436	4.024948719726337e-06
Atp5l	-4.617826	-0.18687893	3.877814293286026e-06
Map2k6	-4.6324453	-0.6661487	3.61372031127295e-06
Stat4	-4.6326385	-0.32705227	3.6103501467436818e-06
Dck	-4.636117	-0.81097436	3.550149016476172e-06
Tagap	-4.642974	-0.5804319	3.4343006903348783e-06
Grap	-4.6485586	-0.39027217	3.342623145645606e-06
Mndal	-4.6544023	-0.26408136	3.249217289533472e-06
Dip2b	-4.657061	-0.3776149	3.2075504256622193e-06
Ypel3	-4.6604805	-0.30733413	3.1547183301463434e-06
Man1c1	-4.662578	-0.5867937	3.1227262969452797e-06
Cnp	-4.6709704	-0.44502175	2.9977993363058183e-06
Samhd1	-4.674577	-0.22248065	2.9455997053879384e-06
Prkx	-4.6811695	-0.4103535	2.852432420432104e-06
Kcnk1	-4.6937566	-0.33167043	2.6823317401805778e-06
Itgb4	-4.6973424	-1.3150815	2.635684776852502e-06
Mosmo	-4.7032423	-0.70461017	2.5606216032684915e-06
Strbp	-4.7107735	-0.42828003	2.4677857237639038e-06
Slain2	-4.7234616	-0.32304975	2.318637163193816e-06
Srpk2	-4.7264266	-0.3205902	2.2850545449890124e-06
Tnfsf8	-4.7295933	-2.581017	2.2496997400454477e-06
Rnf145	-4.7311797	-0.5005728	2.232187234074071e-06
Cblb	-4.7472367	-0.40936202	2.062143099048892e-06
Ift80	-4.769016	-1.6596489	1.8512804430349116e-06
Cysltr2	-4.7883706	-2.0541785	1.6814086090387133e-06
Dock2	-4.7917395	-0.13576521	1.65341437508902e-06

Tmem156	-4.793537	-0.670554	1.638662996722401e-06
Mrps16	-4.7983108	-0.3424142	1.6000921096858405e-06
Gramd1a	-4.8015165	-0.6237529	1.5746855031493905e-06
Hnrnpa1	-4.820316	-0.3546412	1.43331212066303e-06
Slc14a1	-4.828747	-1.8721496	1.3739494517970832e-06
Trim34a	-4.834341	-1.0648681	1.3358751942089035e-06
Sesn3	-4.8455706	-0.49095237	1.2624837493669169e-06
Nr2c2	-4.8464885	-0.39821345	1.2566588778735567e-06
Pcbp2	-4.849486	-0.18099426	1.2378179965741379e-06
5830444B04Rik	-4.8498483	-1.3007469	1.2355584124148905e-06
Slc12a7	-4.873603	-0.58158976	1.095811064856266e-06
Ubald2	-4.887572	-0.6724551	1.0208732867140413e-06
Mien1	-4.899229	-0.32915902	9.6213400185108e-07
Rp9	-4.901805	-0.32202572	9.496017198829989e-07
Fhit	-4.906677	-0.8721407	9.263243675176043e-07
Ip6k1	-4.914873	-0.51341134	8.88398711465054e-07
Nub1	-4.915649	-0.44192615	8.848893953732478e-07
Nsmce1	-4.920987	-0.9782121	8.610873573954694e-07
Raet1e	-4.9229894	-0.5885192	8.523199186396367e-07
Atf7ip	-4.9352827	-0.3107418	8.003472181462771e-07
Tcp11l2	-4.941875	-0.62498623	7.737483299195445e-07
Kdm7a	-4.9459033	-0.49757767	7.579154942567672e-07
Bst2	-4.9495516	-0.5182731	7.438468652642138e-07
Apobec3	-4.951185	-0.2968191	7.376276574243809e-07
Atp5e	-4.952469	-0.20151737	7.32777908841619e-07
Wdr33	-4.9547977	-0.29475537	7.240548658206802e-07
Socs3	-4.968945	-3.4098763	6.731818996603223e-07
A930005H10Rik	-4.972471	-0.69326264	6.61047759301816e-07
Acvr2a	-4.9818053	-1.9725386	6.299373142356735e-07
Eeig1	-4.983089	-0.36322027	6.257718285195762e-07
Ssh2	-4.9865203	-0.26360375	6.14764900108012e-07
Zfp654	-4.990296	-0.43416244	6.028681957002362e-07
Slc9a9	-4.995884	-0.33006656	5.856681623398639e-07
St3gal6	-4.999048	-0.28804454	5.761404737799354e-07
Slc25a3	-5.008522	-0.24857958	5.484962666116048e-07
Skap1	-5.01119	-0.1932577	5.409455287909032e-07
Zbtb44	-5.0124373	-0.67179507	5.374484977260452e-07
D17H6S56E-5	-5.022853	-0.91275156	5.09094895138862e-07
Fut8	-5.0323925	-0.3728911	4.843966924536069e-07
Asap1	-5.059623	-0.78175586	4.200869095690339e-07
Arhgap5	-5.0806174	-0.85399026	3.7621025974746644e-07
Adss	-5.083107	-0.42913172	3.7131063313073485e-07
Kif21b	-5.0962377	-0.43335637	3.4646983643385466e-07
Kxd1	-5.1036053	-0.46201935	3.332427607580951e-07

Dennd4a	-5.112622	-0.70954645	3.1771801618482483e-07
Snhg8	-5.1138577	-0.5544125	3.1564527410967383e-07
Atad2b	-5.1302567	-0.37370402	2.893476818149058e-07
Arhgdib	-5.144466	-0.14588301	2.6828310397491316e-07
Atp5h	-5.1836567	-0.2123077	2.175773478138102e-07
Psme1	-5.190644	-0.29392534	2.0956802919224285e-07
Stk4	-5.1932106	-0.26136938	2.0669800374844247e-07
Itpkb	-5.2001743	-0.42673004	1.9910174431134202e-07
Ddt	-5.2017965	-0.66114134	1.9737144662599655e-07
Ifi208	-5.2090716	-0.92169654	1.897876318904967e-07
Ggt1	-5.2092886	-3.1620486	1.895659527594802e-07
Sesn1	-5.218124	-0.42940784	1.8074458072734707e-07
Sp110	-5.2361865	-0.5863498	1.6392845513043693e-07
Smap1	-5.2365575	-0.24691716	1.6359912227098342e-07
Bik	-5.2370243	-1.0657392	1.631863864990334e-07
Arf5	-5.2472405	-0.15618667	1.5439413460168888e-07
Elovl5	-5.2474637	-0.81095773	1.5420758686431873e-07
Lztfl1	-5.249225	-1.2539015	1.52740271586266e-07
Ubac2	-5.264076	-0.43082008	1.4089598843329873e-07
Cmss1	-5.2829914	-0.14317703	1.2709141654730423e-07
Lrch3	-5.2926674	-0.35060075	1.205451589439553e-07
Ramp3	-5.2988048	-3.393219	1.1656310542880694e-07
Insr	-5.3119745	-2.0682547	1.084438055170569e-07
Pan3	-5.3456483	-0.32891718	9.009390010845486e-08
Asxl2	-5.358735	-0.38953257	8.380673280688787e-08
Klrk1	-5.364855	-0.33013234	8.101439670656227e-08
Insyn2b	-5.368396	-0.40473497	7.944001217117423e-08
Chd7	-5.3759685	-0.52551126	7.617211122556058e-08
Clec2d	-5.378666	-0.33497286	7.5039780345993e-08
Macroh2a1	-5.3846164	-0.35503367	7.259927017660682e-08
Tnfrsf26	-5.385249	-2.0226517	7.234431377951395e-08
Rnf130	-5.40002	-2.0137796	6.663342888764659e-08
Grk6	-5.4217486	-0.49008155	5.901886329215514e-08
S100a10	-5.4360857	-0.117189534	5.446378628859309e-08
Prkcb	-5.4410706	-1.4425024	5.2961306209817584e-08
Sfpq	-5.44441	-0.29277763	5.1977427399649563e-08
Basp1	-5.463209	-3.7806473	4.676033739299672e-08
Oga	-5.4722195	-0.34564802	4.444338825396604e-08
Zc3hav1	-5.4770083	-0.2814335	4.325766215626313e-08
Lysmd2	-5.477968	-0.6179961	4.302379204236362e-08
Ttc28	-5.481673	-1.9481144	4.2132342938121685e-08
Arl5c	-5.500683	-0.737517	3.78323404755897e-08
Ric1	-5.5115647	-0.6120689	3.556574555554058e-08
Ifi206	-5.5142326	-0.53281075	3.503051164854088e-08

Vrk1	-5.5151296	-0.5847077	3.48522727361686e-08
S1pr4	-5.5251026	-0.46911272	3.2929286729792895e-08
Aimp1	-5.525483	-0.4559801	3.285803986406304e-08
Rras2	-5.5352273	-2.5789495	3.108251221415854e-08
Cd74	-5.536157	-1.7500445	3.091802851955723e-08
Abtb2	-5.5510826	-1.8959681	2.8390622534523713e-08
Septin6	-5.5528264	-0.30314475	2.8108742604459398e-08
Tns4	-5.589822	-0.4985263	2.2730295627190013e-08
Tent5a	-5.602786	-0.83930165	2.109333126341669e-08
Mirt1	-5.6151924	-2.1919074	1.9634376966819513e-08
Dpp4	-5.6278834	-2.9369211	1.82434169650388e-08
Rb1cc1	-5.649371	-0.40315574	1.6103563334081265e-08
Ddx6	-5.658967	-0.28888875	1.522867827421167e-08
Fhip1b	-5.668325	-0.7816043	1.4420018115336043e-08
Rasgrp2	-5.683408	-0.37434292	1.320368902376263e-08
Sh2d1a	-5.6834345	-1.9448004	1.3201624009194465e-08
Jak1	-5.712768	-0.19269924	1.1115299186088468e-08
Gramd3	-5.714999	-2.7384803	1.097044477888127e-08
Dennd1b	-5.7169957	-0.28531307	1.084238454642003e-08
Galnt10	-5.7786813	-1.291826	7.528832020522958e-09
Plekha2	-5.7835627	-0.62240523	7.313500938189788e-09
Pik3r5	-5.7882237	-0.64084804	7.113465432918187e-09
Dgkz	-5.7898726	-0.5295101	7.0439892430760946e-09
Rnf138	-5.7984405	-0.47666714	6.69345436023382e-09
Sh3kbp1	-5.820451	-0.26966345	5.868907664528014e-09
Mbp	-5.824946	-0.45278263	5.713115861591734e-09
Bltp1	-5.835427	-0.36756235	5.365307141251828e-09
Hint1	-5.835792	-0.25462553	5.353560506023936e-09
BE692007	-5.8971925	-2.5391765	3.6973806182399683e-09
Arid5b	-5.9232464	-0.44080338	3.1564697338766785e-09
Cbl	-5.942203	-0.41021264	2.8121638297909858e-09
Ogt	-5.9638724	-0.28863135	2.463292706104643e-09
Gphn	-5.97621	-0.35988533	2.2838849558584783e-09
Pxdc1	-5.98009	-4.2347665	2.230144905554497e-09
Sell	-5.9813824	-2.3130534	2.212520272009436e-09
Dnajc3	-5.998934	-0.2956748	1.9861741890858635e-09
L3mbtl3	-6.0089335	-0.69908965	1.867477870602733e-09
Gpcpd1	-6.012145	-0.53049207	1.8308460403238188e-09
Irf2	-6.018844	-0.35738686	1.7566704301127924e-09
Edaradd	-6.0540805	-1.2958413	1.4122180913434706e-09
Vav3	-6.0566626	-3.8445766	1.389748969722299e-09
Gclc	-6.094062	-0.6804806	1.100811698961853e-09
Spint2	-6.106649	-0.76156294	1.0174463360415595e-09
Rreb1	-6.113393	-0.8115152	9.753492997976633e-10

Pfdn5	-6.119349	-0.22094586	9.395829602822656e-10
Foxn3	-6.1365323	-0.32202092	8.434221012171333e-10
Pola1	-6.1439476	-0.78995264	8.0495486310323e-10
Rnf167	-6.1569476	-0.48877656	7.416033932430884e-10
Crlf3	-6.157512	-0.29044372	7.389658824891472e-10
Fyn	-6.1580467	-0.3567662	7.364756298903565e-10
Hlcs	-6.1669803	-0.8726787	6.960652764005989e-10
Nedd4l	-6.180613	-3.0508611	6.385314083606651e-10
Picalm	-6.198842	-0.392612	5.688012666457183e-10
Fyb	-6.2047005	-1.3686203	5.480122338489991e-10
Tm6sf1	-6.2132564	-0.9963386	5.189771344663369e-10
Hipk1	-6.22366	-0.39401805	4.856890427693973e-10
Churc1	-6.241253	-0.55650616	4.340792575569964e-10
Cox8a	-6.2426343	-0.2140202	4.302615111471347e-10
Lrrc8b	-6.2560773	-0.75981593	3.947809292245789e-10
Trim30a	-6.2679396	-0.8636713	3.65856185588201e-10
Zfas1	-6.2681003	-0.9109592	3.654795594503306e-10
Rnf19b	-6.277458	-0.5827078	3.4415266198810907e-10
Chd3	-6.279695	-0.5331755	3.392373202075457e-10
Hnrnpc	-6.3313813	-0.3090083	2.4297612523872787e-10
Usp3	-6.33639	-0.38290626	2.352109239526135e-10
Igf2r	-6.3598742	-0.68122005	2.0191903057130191e-10
Cdk19	-6.395607	-0.5949206	1.5991079649405292e-10
Jakmip1	-6.4088955	-0.65177757	1.4657769849656668e-10
Cmah	-6.49195	-0.6171434	8.473231976686139e-11
4930523C07Rik	-6.494686	-0.49126202	8.320665002368309e-11
Cd27	-6.4971848	-1.8910792	8.183698697984972e-11
Baz2b	-6.5320354	-0.9543879	6.488192796342776e-11
Pabpc1	-6.5443697	-0.23980173	5.97467475542886e-11
Trgv2	-6.5536447	-0.54548085	5.614945400929866e-11
Foxo1	-6.5679255	-0.5628516	5.102109180889287e-11
Ero1a	-6.5784125	-0.4088977	4.7549812672902667e-11
Cdyl2	-6.586392	-0.92656934	4.5064339794786773e-11
Uba52	-6.605426	-0.45619544	3.963763464502892e-11
Adcy7	-6.6107225	-0.5615232	3.8244814457271946e-11
Eif3i	-6.6587014	-0.383308	2.7625790805975455e-11
Sptbn1	-6.702351	-0.454519	2.0509239231157217e-11
Fbxl20	-6.704974	-0.7843898	2.0144166493708615e-11
Crim1	-6.707883	-2.6791391	1.9746838931944985e-11
Pik3ip1	-6.7175736	-1.4097668	1.8477546966078586e-11
Psmb1	-6.7432857	-0.28489882	1.548441208112565e-11
Pag1	-6.7870073	-0.5076789	1.144835334063366e-11
Gpr183	-6.7907624	-0.3933264	1.1154261642856098e-11
Cd79b	-6.7919297	-0.81489956	1.1064340944540904e-11

H3f3b	-6.8233223	-0.22062929	8.895880485128607e-12
Lax1	-6.8293977	-0.4828272	8.527198333199497e-12
Parp8	-6.8445845	-0.94095093	7.669828285197502e-12
Ppp3ca	-6.857118	-0.3037753	7.0263556473371645e-12
Crebrf	-6.864578	-0.5645147	6.668820945440609e-12
Zfp462	-6.887135	-0.8362135	5.692717442628189e-12
Lck	-6.892245	-1.930199	5.4918746098066195e-12
Pglyrp1	-6.893778	-2.7898934	5.4329879106922354e-12
Vps37b	-6.933655	-1.2206517	4.101045858955725e-12
Nr3c1	-6.9423947	-0.44253117	3.855076595380462e-12
Gata3	-6.969839	-0.60761917	3.1730345298164304e-12
Ptpn18	-6.9706564	-0.2583518	3.154661159693292e-12
Dnajc15	-6.9752607	-0.55518186	3.053036808132812e-12
Lmo4	-6.986214	-0.6007373	2.8240270333920457e-12
Atp5g2	-6.994194	-0.33591047	2.6678851069465233e-12
Trpc4ap	-7.0170956	-0.65445566	2.265273995541308e-12
Tox	-7.0684667	-0.42094356	1.5665477475088134e-12
Ankrd11	-7.084652	-0.2693664	1.3939437456167669e-12
Tnrc6b	-7.0959525	-0.3261453	1.2846341661114889e-12
Irak2	-7.09786	-0.9758415	1.26703480843483e-12
Limd2	-7.101419	-0.40527812	1.2348240368637875e-12
Srpk1	-7.104728	-0.41169548	1.2055935205370152e-12
Eif3m	-7.13905	-0.39841816	9.397807010050132e-13
Camk2d	-7.139543	-0.5139023	9.364154048381686e-13
Ddb2	-7.1614738	-1.3084644	7.981431435949928e-13
Hectd1	-7.204372	-0.33653635	5.831192752608652e-13
Trp53inp1	-7.212512	-0.9571533	5.492894962560547e-13
Nsa2	-7.2557936	-0.36789873	3.9931329358070487e-13
Daam1	-7.285157	-0.6674783	3.2129566083289964e-13
Peli1	-7.3036175	-0.70296997	2.8013166764079446e-13
Eif3h	-7.309815	-0.30106327	2.675109105891403e-13
Ahcyl2	-7.3122625	-0.62144375	2.626809115728176e-13
Shank2	-7.344983	-5.48041	2.057850847551142e-13
Paip2	-7.3453784	-0.3965053	2.0517805881321899e-13
Cdkn1b	-7.353649	-0.44710028	1.9286787991629562e-13
Ubb	-7.3945003	-0.17893556	1.4194089608422438e-13
St8sia4	-7.416238	-0.62413186	1.2049408610672022e-13
Nsd3	-7.449653	-0.40191433	9.358579476212774e-14
Prkd2	-7.505953	-0.7216863	6.098328623471752e-14
Epb41	-7.587543	-0.608696	3.260281419393466e-14
Lyst	-7.5898304	-0.99510086	3.2032390060871716e-14
Rnf125	-7.6011553	-1.2380673	2.9349856624524515e-14
Syne1	-7.601675	-0.72582376	2.9232162264573927e-14
Tomm6	-7.605947	-0.39312944	2.828246740572167e-14

Lncpint	-7.630994	-0.5374823	2.3295041200929738e-14
Rarg	-7.6773415	-0.61864877	1.624239731069095e-14
Ppp3cc	-7.701895	-0.8483337	1.3406280768840477e-14
Prkch	-7.710145	-2.4135072	1.2567474148199618e-14
Cox6c	-7.73638	-0.28564256	1.0228721383264763e-14
Eml4	-7.7453194	-0.57074463	9.534186327834072e-15
Acyp2	-7.7637296	-1.1794431	8.246770341797641e-15
Ccm2	-7.7985682	-0.5860688	6.2613577921704585e-15
Jag1	-7.8189387	-3.127593	5.327047720112691e-15
Themis	-7.828505	-2.886223	4.937059695662308e-15
Mrpl30	-7.8565044	-0.6841091	3.950012434598199e-15
Pced1b	-7.8598557	-0.6940565	3.845762250062997e-15
Tmem71	-7.911349	-0.527295	2.5461544212684143e-15
Eya2	-7.919441	-0.8221262	2.3858060547352247e-15
Cd7	-7.9434037	-0.7995642	1.9670765035604533e-15
Cd2	-8.000583	-1.3445965	1.2383191630850918e-15
Jak2	-8.003663	-0.89369106	1.2077134107726867e-15
Stim1	-8.021627	-0.4029418	1.043529899574942e-15
Gmfg	-8.037558	-0.30787218	9.164714233246754e-16
Hivep2	-8.102662	-0.5297262	5.376934441009719e-16
Entrep3	-8.161225	-0.8205315	3.316418050676834e-16
Ighm	-8.208114	-2.394643	2.2469009226670316e-16
P2ry10	-8.221696	-1.3444358	2.0064464709125763e-16
Faah	-8.227121	-1.6267797	1.917666404983454e-16
Nop53	-8.230318	-0.6313756	1.86717668171259e-16
Clec2i	-8.255433	-3.250527	1.5135277990861858e-16
Slc4a7	-8.257093	-0.8051569	1.492625410446567e-16
Cd96	-8.265251	-0.72601604	1.3940078649188784e-16
Cd55	-8.282969	-1.7445136	1.2014155460258722e-16
Pgs1	-8.28913	-1.4622403	1.140792394790084e-16
Clk1	-8.31377	-0.5996228	9.27084218327116e-17
Pip5k1b	-8.347949	-1.9542263	6.945826401955519e-17
Pde5a	-8.358434	-0.8670106	6.355655204547083e-17
Itgb3	-8.387827	-1.4662324	4.952120722622531e-17
AB124611	-8.428701	-1.0442905	3.4952094570618737e-17
Ltb	-8.438535	-0.29625687	3.213392642057298e-17
Park7	-8.4677	-0.43496782	2.5028693708801392e-17
Dennd4c	-8.491798	-0.6335828	2.0346325250763243e-17
Slfn5	-8.504838	-2.0187397	1.8185008878275228e-17
Tut4	-8.52255	-0.5194789	1.5607898726682577e-17
Klhl24	-8.621106	-1.085569	6.631061465823906e-18
Zswim6	-8.621525	-0.7839785	6.606844846188847e-18
Tspan32	-8.652977	-1.6358591	5.01733392385172e-18
Fam117a	-8.655306	-0.88763934	4.915924756477758e-18

Sp100	-8.675879	-0.3905904	4.103702826379091e-18
Mllt3	-8.685177	-0.5149441	3.781528785028788e-18
Cnot6l	-8.6867695	-0.5561728	3.728919462754951e-18
Tgfbr2	-8.712345	-0.50614506	2.976520390252276e-18
Eif3k	-8.718563	-0.4268353	2.8175493226386754e-18
Madd	-8.7431965	-0.71697	2.2660542183953708e-18
Snrpg	-8.783813	-0.30602044	1.5802345709569624e-18
Atp1b3	-8.80032	-0.3547783	1.3642731412162679e-18
Nebl	-8.835114	-0.54859334	9.999675171421587e-19
Hs3st3b1	-8.867177	-1.5947866	7.502341836316282e-19
Npm1	-8.871934	-0.3440182	7.188668440630813e-19
Cox4i1	-8.915911	-0.30421153	4.838250207897497e-19
Airn	-8.921959	-1.2393115	4.58112401680896e-19
Ctse	-9.015813	-1.0601939	1.9541658127068795e-19
Foxp1	-9.0171995	-0.5066466	1.9295830266366736e-19
Ripor2	-9.050446	-0.3294093	1.4238553039626472e-19
Uqcrh	-9.081506	-0.3511219	1.0708301198324453e-19
Eif3e	-9.129116	-0.45069572	6.906100212514942e-20
2810001G20Rik	-9.1373625	-1.1280351	6.399381093409544e-20
Map4k4	-9.215043	-0.6536493	3.111527180339416e-20
H3f3a	-9.245845	-0.19884455	2.3339017214184165e-20
Cxcr3	-9.253046	-3.4022882	2.1818491920362826e-20
2410006H16Rik	-9.320311	-0.75595015	1.1600013691598305e-20
Cdc25b	-9.326531	-0.9661756	1.0939213497393616e-20
Cd84	-9.343275	-0.7001108	9.339949801926573e-21
Trim12a	-9.375333	-1.0653574	6.895781820356716e-21
Nfatc3	-9.393648	-0.3844783	5.795741369483604e-21
Retreg1	-9.406806	-0.5820333	5.1144431531123804e-21
Npc2	-9.473853	-0.47514206	2.6970589628764485e-21
Ifi203	-9.57156	-0.5376607	1.053042895593803e-21
Trbc1	-9.572556	-0.65047	1.0429527395901732e-21
Trio	-9.609601	-1.5709807	7.283037899741497e-22
AW112010	-9.836321	-0.8460028	7.85300719473946e-23
Klf2	-9.938351	-0.4873137	2.834828077840853e-23
Zeb1	-10.004409	-0.86197364	1.4575932709709732e-23
Rasa3	-10.006114	-0.56200117	1.4326961227740124e-23
Ikzf1	-10.00617	-0.6002955	1.431879242382331e-23
Eef1d	-10.010975	-0.50966805	1.3640222396980802e-23
Arhgef18	-10.014679	-0.7814851	1.3138759422268993e-23
Hgsnat	-10.017142	-1.3579215	1.2815536871231619e-23
Dgka	-10.040977	-1.3465079	1.006724130808743e-23
Tle4	-10.085209	-0.8686265	6.422841647876595e-24
Ramp1	-10.098391	-0.72687095	5.615633731433436e-24
Fgf13	-10.134848	-1.1480997	3.869748337108664e-24

B4galnt1	-10.223829	-0.94433373	1.5509022330866984e-24
Add1	-10.269951	-0.6180078	9.625336691889405e-25
Sub1	-10.296466	-0.35016403	7.309785340218711e-25
Ppp1r12a	-10.324234	-0.4086576	5.475428207521326e-25
Elf1	-10.354779	-0.46988398	3.981030637553077e-25
Sh3bp5	-10.361274	-1.6412174	3.719756237671533e-25
Itk	-10.44566	-0.61069745	1.533859708283071e-25
Zfand6	-10.471766	-0.551458	1.1644918893313839e-25
Epsti1	-10.491127	-0.7750342	9.488937924070926e-26
Supt4a	-10.524436	-0.46774578	6.665946690550561e-26
Nfkb1	-10.672301	-0.66701305	1.3718535233704218e-26
Trgc2	-10.772863	-0.8738977	4.623931685316076e-27
Eef1g	-10.816798	-0.5140333	2.8661494540792356e-27
Btf3	-10.877121	-0.29865432	1.4816959533677335e-27
Trerf1	-10.885554	-1.7696297	1.3507371321518397e-27
Btg2	-10.899568	-0.65220463	1.1580578995533493e-27
Fcho2	-10.972822	-0.896976	5.163519526223343e-28
Sgms1	-11.046388	-0.7071545	2.2821323049197953e-28
Arhgap45	-11.106362	-0.47126395	1.1682184698820483e-28
Atp8a1	-11.279893	-0.9336678	1.6494649038330793e-29
Bcl2	-11.283158	-2.0933266	1.58936208946555e-29
Ablim1	-11.350236	-0.4685417	7.396310638539965e-30
Pard3	-11.4305725	-4.2796082	2.941644635063922e-30
Rgcc	-11.472562	-1.2048364	1.8121475879302946e-30
Eif3f	-11.487095	-0.4849458	1.5317747193238355e-30
Sptssa	-11.521306	-0.78887224	1.0303696799106953e-30
Man1a	-11.532019	-0.80695087	9.098458912338063e-31
Pcgf5	-11.539746	-1.1901482	8.317040907584778e-31
Ube2h	-11.747209	-0.84496593	7.298994556881751e-32
Arhgap15	-11.89342	-0.4042447	1.280535535542349e-32
Ppdpf	-11.905131	-0.8555351	1.1128857539483565e-32
Aff3	-12.050252	-3.4956834	1.9335892467896498e-33
Cryba4	-12.190441	-1.5745443	3.4952601199847806e-34
Cd53	-12.260064	-0.5044079	1.4838188979875455e-34
Thada	-12.436031	-1.0168183	1.665776725495478e-35
Slfn2	-12.476564	-0.68971926	1.00218167757108e-35
Rapgef6	-12.497557	-0.5724789	7.698148108837365e-36
Oprm1	-12.536886	-1.0046195	4.690638508798263e-36
Eef2	-12.547228	-0.50544095	4.116702223569157e-36
Tmem64	-12.642739	-0.71891135	1.227032835384585e-36
Rsrp1	-12.74667	-0.53044975	3.253490310530766e-37
Scml4	-12.987397	-1.9470237	1.442504667915746e-38
Mrps5	-12.988885	-1.1887642	1.4147268881354415e-38
Dtx1	-13.029846	-1.1749507	8.277385305643857e-39

Trbc2	-13.03012	-0.48516715	8.247788674787986e-39
Cox7a2l	-13.058925	-0.6446831	5.652037431666412e-39
Zbtb20	-13.096901	-1.0138998	3.4297989677523683e-39
St6gal1	-13.195333	-1.782091	9.334251493036675e-40
Zfp36l2	-13.404524	-1.1120799	5.688698501712629e-41
Ptprc	-13.413728	-0.45282692	5.024827288173086e-41
Klf3	-13.6138315	-1.5514029	3.314017422950439e-42
Gimap6	-13.801732	-1.0783546	2.488015168834389e-43
Gas5	-13.852702	-0.8433846	1.2251493950999467e-43
Adgre5	-13.952221	-1.167949	3.0495812006534914e-44
Itga4	-14.0064945	-1.5443947	1.4225497954167875e-44
Pdlim1	-14.084657	-1.648832	4.719480894784855e-45
Sidt1	-14.185714	-3.6292708	1.1231633334972598e-45
S1pr1	-14.207868	-0.8961537	8.188113820945533e-46
Atp2b1	-14.428847	-0.55336356	3.4072648797341774e-47
Pde7a	-14.529266	-1.0830559	7.906230908639937e-48
Ccr6	-14.938474	-5.7526064	1.8516774681157506e-50
Clint1	-14.944005	-0.53826433	1.7041654853661126e-50
Eef1a1	-14.965089	-0.27964273	1.2415736518417723e-50
Gpr132	-15.025764	-1.1533298	4.9783393342715224e-51
Kbtbd11	-15.031588	-3.434621	4.5594579227066093e-51
Utrn	-15.059593	-0.78023964	2.9861813981238426e-51
Fchsd2	-15.123647	-1.0553927	1.131017007473835e-51
Crip1	-15.153786	-0.55486345	7.15252916111283e-52
Bach2	-15.182766	-2.3391144	4.599706528256919e-52
Stk38	-15.322691	-1.0517684	5.393781735972374e-53
Klhl6	-15.470149	-0.94631433	5.517899665921695e-54
Fam107b	-15.720591	-0.92582023	1.0929978180714305e-55
Tasp1	-16.039444	-1.4660115	6.7758333530374e-58
Ipcef1	-16.07592	-1.0172942	3.7636759306585455e-58
Hmgb2	-16.209244	-0.7617958	4.338795000180022e-59
Stmp1	-16.280457	-0.9327386	1.3585251696571527e-59
Lef1	-16.453028	-1.9773172	7.977936915254296e-61
Igf1r	-16.576609	-1.0347495	1.0287146625938522e-61
Tpt1	-17.124292	-0.26014116	9.77926937911506e-66
Txk	-17.47542	-1.8612833	2.2051781309984516e-68
Ly6e	-17.519518	-0.8080704	1.016830769649531e-68
Fau	-17.608952	-0.3599504	2.1030614541448584e-69
Esyt2	-18.088741	-1.1504939	3.90912388683131e-73
Pdcd4	-18.363163	-0.9932535	2.590669100637001e-75
Camk4	-18.5807	-1.0829141	4.604573345421993e-77
Naca	-18.660074	-0.60670346	1.0458596599137686e-77
Evl	-18.868132	-2.7103055	2.0854365096164926e-79
Eef1b2	-19.199085	-0.6299331	3.766724264267522e-82

Scpep1	-19.583382	-1.5668669	2.1431026262970342e-85
Rack1	-20.617298	-0.5309179	1.9198683852848166e-94
Prkcq	-20.649055	-1.5737903	9.954691922860816e-95
Ms4a4b	-20.992882	-2.3028867	7.618475636585107e-98
Nav2	-21.27673	-2.7343638	1.8650703185349674e-100
Uhrf2	-21.78838	-1.5113598	2.9905197452825896e-105
Tmsb10	-21.926785	-0.43338782	1.4426634750294212e-106
Shisa5	-22.62017	-1.1167581	2.744174852422307e-113
Mbnl1	-23.176062	-0.73495	7.940378709425961e-119
Ms4a6b	-23.604647	-1.9792935	3.453104837101534e-123
Rplp0	-23.864042	-0.7179222	7.2395221133053e-126
Il23r	-25.163824	-1.7727617	9.975325184794465e-140
Rpsa	-26.006477	-0.7516248	4.183455592284266e-149
Rplp2	-26.198315	-0.5124267	2.7775978937075962e-151
Il18r1	-26.491184	-1.4695268	1.2246509902796053e-154
Ifngr1	-27.374323	-2.537522	5.545685354120126e-165
Tcf7	-28.133621	-3.1077132	3.8017195974211906e-174
Rplp1	-29.265226	-0.599582	2.8745368964465555e-188
Il7r	-37.64193	-1.9407157	4.43395094329943e-310

Column5
pvals_adj
3.9910190062694e-260
1.1003314223666081e-243
2.1850307256110143e-171
6.785068463887388e-153
7.542575700831311e-153
2.065788573594673e-138
9.340764517526891e-137
2.9616456078396167e-112
6.017029941321082e-100
6.656301321760035e-99
3.279271830767545e-96
8.577920992520638e-95
3.147869930450765e-92
4.4438807991973846e-89
5.707684682066528e-85
2.356909864165361e-84
3.9170168730525515e-82
5.450498530991614e-82
8.163283355085939e-82
8.498264125031067e-82
4.1867912439586506e-81
5.781239439209178e-79
7.76891139812882e-79
5.796720303806414e-77
6.768411953890581e-71
1.6751407825562797e-70
2.6613180898013917e-69
1.7068924895176547e-68
3.718309534760892e-68
7.276318469022407e-68
2.7812353353376663e-67
1.5466155651012497e-66
3.201060419463108e-64
4.4217776172207045e-64
3.1771849617176005e-63
4.9310335059915005e-60
1.1114253756397916e-57
3.382633669350964e-57
5.153355325326232e-57
1.0821777823040497e-56
2.4303468696874048e-56
3.1960670969080943e-55

5.5393674606414696e-55
1.3907979741704606e-52
8.068496383514595e-50
9.868721592607946e-50
1.3859486654229592e-49
3.92168931317094e-49
1.1984676627744887e-48
3.228486378677591e-46
6.42570023042803e-46
8.000824459390411e-46
1.4186206389404224e-45
1.4542723749954573e-45
1.372100664814092e-44
5.40538379394931e-44
7.419106372968962e-44
1.960344654478842e-43
4.885702335764391e-43
4.91257820050969e-42
7.476262061817678e-42
1.9370082986055867e-40
3.623490940753351e-38
3.863126823398276e-38
5.567320303865409e-38
1.3080019047397906e-37
2.3798636079408436e-36
3.656305471695531e-36
8.86956553220826e-36
1.2348320684118221e-35
2.548578157766158e-35
3.826815280596977e-34
8.008825298924478e-34
9.719310167481769e-34
8.848575448341813e-33
2.5556375700999123e-32
6.4187813932954115e-31
6.8632492245392514e-31
6.8632492245392514e-31
6.886668696525632e-31
7.099083741851269e-31
2.1657026596076445e-30
4.130699645058312e-30
5.382538319246974e-30
7.062144600079836e-30
1.3524211454308628e-29

2.8051699172822945e-29
3.049433666024635e-29
4.167265563682953e-29
1.7911234203966095e-28
2.260684165454271e-28
3.3472694114836104e-28
4.313387244937843e-28
6.6729556408581865e-28
7.191988112096201e-28
7.664795160395483e-28
1.2778977982518154e-27
2.008424296283076e-27
5.3082956117552154e-27
5.637842733460885e-27
6.343554481976848e-27
1.0566655144505976e-26
1.2212538145743919e-26
2.5525070976279572e-26
2.711837781461351e-26
6.284385552541152e-26
8.271091989632068e-26
9.908487903889504e-26
1.2169593749297605e-25
1.9859742716622394e-25
2.8613774124297655e-25
1.967366432184604e-24
3.1215172998465458e-24
4.3733120483551415e-24
5.037041922168116e-24
7.399920959464791e-24
1.1831772947185482e-23
1.1971720464251583e-23
7.412633677337227e-23
7.412633677337227e-23
3.403788887580483e-22
3.735832500002759e-22
4.909459326013089e-22
6.411733356327849e-21
6.411733356327849e-21
1.937804451874294e-20
2.914294466396309e-20
3.0892397565701684e-20
3.3301499872563876e-20
6.385605642660822e-20

1.5732641703011503e-19
1.7936054977919e-19
2.519705314881064e-19
2.7726280158356225e-19
2.8541656469397287e-19
3.7517251468474202e-19
4.260292304605673e-19
5.13716973796333e-19
8.132957751569691e-19
1.235772460201709e-18
2.121853004706964e-18
2.1488632540432188e-18
3.531376234890108e-18
3.705478837850213e-18
3.8479010125499354e-18
5.14554388108288e-18
5.2920411046133715e-18
5.367096867551352e-18
5.711875071298853e-18
7.469507682613159e-18
9.027782973349847e-18
1.0475703016445869e-17
1.204450868783691e-17
1.2071187639875114e-17
2.2614400122393173e-17
2.3923584837375854e-17
2.404397845634495e-17
2.9017362555305 37e-17
6.145576519620773e-17
8.159445800672931e-17
1.098814382463933e-16
1.404124044967387e-16
1.5871370065171435e-16
1.5994284142203422e-16
1.757853001977773e-16
1.9824701223139914e-16
2.474298516058025e-16
2.495932518353311e-16
2.714251436379155e-16
3.6575130047533493e-16
3.962216082054081e-16
4.714984559745665e-16
5.817683040970432e-16
6.813684772811268e-16

8.154674220536931e-16
8.634530031676836e-16
1.3974063204157493e-15
1.9400646291632262e-15
3.555156993921454e-15
4.204705516860541e-15
5.361873247045945e-15
6.860510639452982e-15
7.668087674405677e-15
7.668087674405677e-15
1.039260153602338e-14
1.642792526843838e-14
1.7033226793573542e-14
1.9183291654557015e-14
2.1470998421749428e-14
2.2351362191429826e-14
2.59994058119667e-14
2.832785744108307e-14
2.959519180971742e-14
3.5808867013762584e-14
3.867244244920491e-14
4.0136601793705584e-14
4.2213055230035674e-14
4.692363082104003e-14
4.877830862574767e-14
5.1156835604229426e-14
6.598015800406685e-14
9.607583119834641e-14
1.0243523148431707e-13
1.305739362162674e-13
1.4122549296538987e-13
1.577165580802339e-13
1.6882340915835512e-13
1.8434278843689414e-13
2.1022434864897392e-13
2.1335449011192902e-13
2.2083275070689192e-13
2.9094989371382314e-13
3.4265097312983997e-13
4.219993909389293e-13
4.2899539401583316e-13
4.35674576928734e-13
4.4173208204419054e-13
4.861049277788198e-13

5.957009272698122e-13
8.19983036175836e-13
8.549270215295102e-13
9.145245488264175e-13
9.499390077094054e-13
1.3037644492512911e-12
1.4186091662181596e-12
1.4488433244506895e-12
1.589887412061465e-12
1.7196085435063825e-12
1.8848733176892204e-12
2.3100378469304334e-12
3.938414971292925e-12
3.942348102195174e-12
4.9749298676923545e-12
5.496336962506796e-12
8.068274281604539e-12
1.0119898823086673e-11
1.2510350321285996e-11
1.681105507859142e-11
1.70415303672583e-11
1.9164772247841807e-11
2.0452696551761383e-11
2.6915504235824344e-11
3.2759175356025793e-11
3.750442188281965e-11
4.085083158276998e-11
5.229139849406123e-11
5.8136534375390325e-11
6.251706003295072e-11
6.652849273088926e-11
6.793296138424947e-11
7.078610056344195e-11
7.426489167600344e-11
7.646427379332616e-11
8.622774778940496e-11
9.470590294240703e-11
1.177306974799104e-10
1.234683850419305e-10
1.3680792554952594e-10
1.4488766576084281e-10
1.483340049648837e-10
1.52847532483193e-10
1.6474111256892688e-10

1.671370623421025e-10
2.0838212552874717e-10
2.2890903679232038e-10
2.3255697125360643e-10
2.489172291016475e-10
2.630670926873462e-10
2.6417652129480636e-10
2.6583751198373995e-10
2.9927046895891396e-10
3.619094543678197e-10
4.034695506865916e-10
4.388561996423877e-10
4.4870082076340545e-10
4.5349906812946735e-10
4.761630935426156e-10
4.774949004310932e-10
4.96161285864602e-10
5.307922848935668e-10
5.68593412981686e-10
5.951710854859928e-10
7.380701754897429e-10
7.381485897767022e-10
8.388768477554537e-10
1.0751326519393227e-09
1.1207158638290526e-09
1.233037494950453e-09
1.2561212293762968e-09
1.2703628157112566e-09
1.2766151107514971e-09
1.2889674384798481e-09
1.3069349822903192e-09
1.786018682217285e-09
1.9785460377574336e-09
2.2932487944124083e-09
2.5572660294740886e-09
2.574740389943251e-09
2.6605523024322474e-09
2.7323969345956744e-09
3.1578729234102173e-09
3.1656626536019315e-09
3.476656714414553e-09
3.6157381011575834e-09
3.6157381011575834e-09
3.7439817467281825e-09

4.2975252399397184e-09
4.320711879826318e-09
4.725646482209981e-09
4.726278736989933e-09
5.128443718425882e-09
5.2809971746003764e-09
6.095434425500506e-09
6.262173952159964e-09
6.44424243603862e-09
6.816675837948532e-09
6.8345339557509476e-09
7.253120220843743e-09
8.285114593184384e-09
8.402454652748971e-09
8.883912047242674e-09
9.976779171488588e-09
1.0006389904955792e-08
1.038757790377364e-08
1.0983950494236004e-08
1.1476950947992822e-08
1.3219308244750707e-08
1.3909590898140707e-08
1.4652499657501692e-08
1.5557553300261996e-08
1.5711139004509764e-08
1.6498214296234224e-08
1.6980009385388655e-08
1.717524145571169e-08
1.8041448713878832e-08
2.051845636896201e-08
2.0760871892097858e-08
2.150996340773973e-08
2.3028121748538016e-08
2.3666154767811025e-08
2.624296866812008e-08
2.6298900462487947e-08
2.8082230968395613e-08
3.0593455432183054e-08
3.5155958625684604e-08
4.031778319447432e-08
4.147909303186115e-08
4.3437346278042904e-08
4.350437893534194e-08
4.365434837763785e-08

4.4262193373588456e-08
4.9320632165515635e-08
4.946022327470961e-08
5.0056800707224076e-08
5.062229763875111e-08
6.130642211238564e-08
6.76279158686582e-08
7.127212967888675e-08
7.392923168522014e-08
7.526976802346459e-08
8.257435180085127e-08
8.430185285220218e-08
8.715905083395566e-08
8.805766773002952e-08
8.913715795645853e-08
8.943154117310004e-08
9.02127842681617e-08
9.583420020865008e-08
9.99798193616767e-08
1.179019247873078e-07
1.2330330989416767e-07
1.3063411627925676e-07
1.32244630091815e-07
1.3289064601821257e-07
1.3819666907308565e-07
1.5499967614825566e-07
1.6198015419019262e-07
1.746750713291267e-07
1.7783972672334284e-07
1.827586620097985e-07
1.9435871743197728e-07
1.960085909060831e-07
2.1212650966627767e-07
2.1275527181722933e-07
2.1935003181540508e-07
2.3900938584816597e-07
2.5521650208171183e-07
2.701244934027795e-07
3.1060702738369286e-07
3.2129072479034815e-07
3.4955196366844296e-07
3.618535080604997e-07
4.500134701042184e-07
4.698248894436835e-07

5.159940157210494e-07
5.325050882766905e-07
6.420208427189681e-07
6.545260283489332e-07
6.719557920868006e-07
6.897180251746688e-07
7.177943169761168e-07
7.219815098755603e-07
7.592575899241667e-07
8.416268953908732e-07
9.67103982004405e-07
9.673034381923718e-07
9.73435000904519e-07
9.829410600727932e-07
1.1253279644873549e-06
1.2437077590183185e-06
1.3850922336945365e-06
1.4520492959385367e-06
1.505207702565721e-06
1.529274826597783e-06
1.5762071600936687e-06
1.5810738994053648e-06
1.662947687269367e-06
1.7039296606452438e-06
2.0313182469254392e-06
2.1469710002036862e-06
2.194268771237119e-06
2.194268771237119e-06
2.243667483034836e-06
2.2763369344797303e-06
2.7815801677279028e-06
2.80920120899557e-06
2.935468239365131e-06
2.967794866041267e-06
3.7112987272447007e-06
3.932133578028831e-06
4.168634450217306e-06
4.177892726484271e-06
4.2459722641269235e-06
4.420774597998367e-06
4.44578221198073e-06
4.660451897830098e-06
5.201753108017137e-06
5.315730183022039e-06

5.947994917760701e-06
6.966992644761877e-06
7.729389555552644e-06
8.054414422869242e-06
8.250193566619661e-06
8.282115778039917e-06
8.66923468786129e-06
8.757831332678982e-06
8.812431732366647e-06
9.134701992339152e-06
9.799312755749473e-06
9.95534611764606e-06
1.006440521515646e-05
1.0098893557523325e-05
1.0393843617271931e-05
1.0700285935174009e-05
1.101332216676759e-05
1.1458780238426591e-05
1.146046087908644e-05
1.1899547265874828e-05
1.2368619921450512e-05
1.2459648585219117e-05
1.3536196244307363e-05
1.5161266439859199e-05
1.570784301444543e-05
1.5753397914485463e-05
1.6194376484322522e-05
1.6841585423040672e-05
1.6977933398150516e-05
1.704237928957368e-05
1.751411203789213e-05
1.8600762926975218e-05
1.8738209378832468e-05
1.948058995576616e-05
1.986290945961985e-05
2.0748230209412553e-05
2.0790104474366586e-05
2.097280341821518e-05
2.2082407123174196e-05
2.466629177073283e-05
2.467795980475166e-05
2.669714156003599e-05
2.740146593974079e-05
2.742835702264918e-05

2.9099232281524202e-05
3.102256908572667e-05
3.3645744972831085e-05
3.384666519881391e-05
3.395816311516717e-05
3.494816057731484e-05
3.592959623294915e-05
3.622914831719726e-05
3.756343838521612e-05
3.862257380478401e-05
3.8761228470556104e-05
4.6601500642218074e-05
4.684818701333588e-05
5.3800421229840796e-05
5.4479111614618194e-05
5.7059959744182473e-05
6.247851180094788e-05
6.328606153127868e-05
6.535048429527359e-05
7.262709931867291e-05
7.315665668384438e-05
7.806468598507967e-05
8.091766821688131e-05
8.164693772946467e-05
9.092259377739223e-05
9.829608613228587e-05
0.00010012810577415124
0.00010289111413282811
0.00010753578387981732
0.00011456527003642407
0.00011611899460969612
0.00011936236765362988
0.00012224218633095338
0.00012360076145871316
0.00012465684096262647
0.00012509283080957978
0.00012652723405579168
0.00013687832244735189
0.00013782478914928865
0.0001380453327689786
0.00014628950458149954
0.00014837324842759597
0.0001570366835896581
0.0001587779482945792

0.00016384128374764546
0.0001656864691890083２
0.00016878805581827615
0.0001693996273150551
0.00017341706383156987
0.00017341706383156987
0.00017390820336178622
0.000175235927888619
0.000178785388017626
0.00018488818081683017
0.00019175016330459982
0.00019323196367209333
0.0001962988222876663
0.0002047678167847046
0.0002083708547020943
0.0002085765965458559
0.00021420973694034141
0.00021747850119364307
0.00022673395513563073
0.00022806110779587774
0.00022942590535951515
0.00023353457541265438
0.00023650774885830092
0.00023683432163408254
0.0002375186245247526
0.00024141172875454894
0.0002512496018489016
0.00027662730069565923
0.00027709851343086204
0.0002949372351365807
0.00029977592257476865
0.00030840417745455975
0.000312608054587761
0.00031782865212193694
0.00031785478493270044
0.0003237112343012797
0.00033786969389527184
0.0003394357835335852
0.00034172394866330204
0.0003593602840685244
0.00036593330851639704
0.000382327868859124
0.0003904972733682911
0.00040245671442914544

0.0004136134890907
0.0004247558557754118
0.000442463678846203
0.0004486860212590664
0.0004505310973073317
0.0004601366633596926
0.00046636009433086677
0.0005053138313035617
0.0005092487499370511
0.0005181638300733897
0.0005247146594619375
0.0005334838022159703
0.0005415895265572931
0.0005492221239425315
0.000555143003360221
0.0005710847395905797
0.0006028199014887867
0.0006084176710223582
0.0006138318016681167
0.0006492642894967387
0.0006538686590296107
0.0006550928598420905
0.0006603559963792968
0.0006649242680926846
0.0007151384494103726
0.000724525925030443
0.0007420355887501508
0.0007448107971004574
0.0007493072634367439
0.0008030065258703552
0.0008124899356237883
0.0008279656032907383
0.0008364118676060917
0.0008764316421670373
0.0008939107470648307
0.001015009909020758
0.0010360241770848396
0.0011140267942568792
0.0011231859395230373
0.0011485412383340332
0.0011686794813269119
0.001188124280641745
0.0011995392213434595
0.0012049384008631422

0.0012242587525160052
0.0012442617799690095
0.0012678704272252553
0.0012876060228285168
0.0013107625676884848
0.0014012550895252291
0.001409589713597721
0.0014247180375276099
0.0014489026085764975
0.001457093131627168
0.0015671271575514495
0.001577569740800653
0.0015846817194630283
0.001637759811945305
0.0016506880130579295
0.0016530877732109303
0.0016570518652421426
0.0016736045411764558
0.0016779568883470862
0.001705165462401593
0.0017203918687619276
0.0017463796250626864
0.0017593879714909565
0.001787860393154946
0.0018458087555956954
0.0018519216314165016
0.001904088764250312
0.0019230678502852156
0.0019275936034177893
0.0019486007161550844
0.001969158331098727
0.002049500571218296
0.0020923987312337885
0.002101856013049987
0.002128719785092642
0.0021376684004663286
0.0021408999216007193
0.0022541670902496047
0.002269321467014692
0.0023186425615902215
0.002327535049933009
0.0023472030370148373
0.002360215434839886
0.002369046240450629

0.0023739103029191437
0.002375898771992185
0.0023810614316442406
0.002393185542750462
0.0024016842801057752
0.0024016842801057752
0.0024210511200055563
0.0025312916766039753
0.0026706588091189353
0.002688142088014739
0.0028167051980095958
0.0028430156295980603
0.002968493187153588
0.002995732066387569
0.002995732066387569
0.0030490654511034767
0.0030569209151521675
0.0031923332131699594
0.003226622511708064
0.0033565638031827823
0.003384231545356208
0.003384231545356208
0.0034193422261097365
0.00344502149282148
0.0034631520051490354
0.003469313647935583
0.0036578631432577576
0.0038197840267135065
0.0038906940369595588
0.003940574084739079
0.003963731626745664
0.004069817596395488
0.004192527606542341
0.004192889514345429
0.004239046907164812
0.004304805217112661
0.004383841917427603
0.004651378017913125
0.00465251626019013
0.004656886649981482
0.004723967270163921
0.004761265432536094
0.0049607214626248555
0.00498888630235267

0.005114220836465403
0.0053976885996258155
0.005402471097188254
0.005441480205199387
0.0054423855868536725
0.005444150848838195
0.005445000660670503
0.005510379410267146
0.005535192637786219
0.005649543942668816
0.005769638586779965
0.005927052707035598
0.005952007524032861
0.0060292133086178165
0.006208040607024495
0.006271845627680777
0.006585516873199612
0.00666197858365038
0.0067529937007974015
0.006778992768094187
0.006873477554290392
0.006894648860469575
0.0073405471867335976
0.0073566479480679245
0.00741082512673633
0.007451537391060991
0.007465244907063215
0.007539842956228161
0.007551132873137721
0.00755281784680975
0.0075634670681207
0.007644447458894448
0.007689542535663868
0.007888085554618115
0.007957010018652603
0.008179536998242806
0.008194856096268905
0.0082943741320271
0.008399546421664609
0.008605356889319736
0.00865680532077176
0.008768573787355996
0.008818436124476296
0.009005689929518059

0.00919638956459618
0.0092417023499021
0.00931613381716852
0.00936500871215564
0.00941342963298109
0.00994543895613307
0.00994543895613307
0.0100577703195761
0.0102484481334464
0.0102554720245120
0.0102554720245120
0.0104285908703626
0.0107235873063114
0.0110714449399052
0.0111995032263490
0.0112287465990819
0.011319821921421
0.011319821921421
0.011319821921421
0.0113552797609865
0.0115131437479743
0.0117050558214491
0.0117317340800630
0.012257979729714
0.012365402423838
0.0124686062837789
0.012756584183736
0.0128056069114662
0.012954981990735
0.013003664971961
0.0132432697591726
0.0136713469629061
0.0136991946418247
0.0139466650251255
0.01468093128494
0.0149266333425197
0.0149486033307960
0.0149911866623160
0.0151150755649954
0.0153732201123584
0.0154609222159767
0.015682221074318
0.015717905895741
0.0157339234534099

0.016228799071111553
0.016266458790709874
0.01638521661260893
0.01638521661260893
0.01664795583634553
0.016815780966813458
0.01690282995505856
0.016995992500391157
0.017285976791904082
0.01729838836569528
0.01743462709261715
0.017485996382816584
0.01751883120341223
0.017620913157049726
0.017732257180101272
0.01775608121756062
0.017854759042896847
0.017854759042896847
0.018016883630660604
0.018278906804446392
0.0185580286278335
0.018601267813815714
0.018831251742853015
0.018925076902297066
0.01895340636126876
0.018963790016324634
0.018963790016324634
0.019169707344803236
0.019335890885394526
0.019504446965745912
0.019513304201657325
0.019772865707601884
0.019874200838260105
0.020015873055916567
0.02003625644442615
0.02003625644442615
0.02006457058723778
0.02006457058723778
0.02013387329532764
0.02029160222499587
0.02030679798610701
0.020421809239033986
0.020535520374724316
0.02058243554155445

0.020583310870784337
0.02070786346515625
0.020887538016724026
0.021011493918853583
0.02113649281400017
0.02117295361002919
0.021742173085952575
0.021939086223375287
0.021959847523966295
0.021976528872164097
0.021980024189416542
0.02221149629769038
0.022415262710657614
0.023091913970032154
0.023091913970032154
0.02309803223316571
0.023216688205716592
0.02349918008493922
0.023650307907804397
0.02456515567258488
0.025103327928551265
0.025203250800711986
0.025410895698196944
0.02638303780527294
0.02638303780527294
0.026494505496491322
0.02673109359386321
0.026793775517866946
0.02689418317715954
0.027731824861900598
0.02814036669149665
0.028248901289279853
0.028580245455223224
0.028651650143086408
0.028723983621138465
0.028806138752957886
0.029200967987698903
0.029686734250547266
0.02993887450468205
0.030024007976586598
0.030025022152625563
0.030492339959845134
0.030905018690921073
0.030933054377667583

0.03095901839589313
0.030975696559030576
0.031072419346198057
0.03140935124468917
0.03140935124468917
0.032119650083271556
0.032311088318489216
0.03236330605555949
0.03236330605555949
0.03280057381732089
0.033003978380433355
0.033148780478999516
0.03318319684038808
0.033472088891331485
0.03351520159072208
0.03358221341375568
0.03361218856545266
0.03375107144894481
0.03382118530836343
0.03411086024182382
0.03417706337872998
0.03434935155656044
0.03446652587782109
0.03446652587782109
0.03457776092095395
0.03477121476435438
0.034893405719733095
0.034893405719733095
0.03576399994900059
0.03602674850342185
0.03721356425888247
0.038442536638918855
0.03872320753830831
0.03886514993405545
0.03944808336248892
0.03949177462092733
0.04026531282845613
0.04063605914305989
0.0406809292028054
0.04099943620811904
0.041403535830730656
0.041750683335338275
0.04235483704765868
0.042569870241333976

0.04304524828428356
0.043748457198095315
0.0439217409080944
0.04459976356757514
0.04463443807565685
0.04476353295477999
0.04490218671011453
0.04512462858504414
0.04536668291151732
0.04543171190293078
0.045486949991019474
0.045652302732853395
0.0457048114634442
0.0459522792738949
0.04679713544887098
0.04735032716687476
0.04740811325536522
0.04766339669500067
0.048173455993287394
0.048296086908922224
0.048384048050628715
0.048453119735140854
0.04890218892249357
0.04966015506634489
0.04848433790565108
0.048453119735140854
0.04794568947702583
0.04784958068596052
0.04744043316223575
0.046726548031228866
0.04671551458997873
0.04647145834116224
0.0459522792738949
0.04543171190293078
0.045274848735443365
0.04508874969275247
0.044183086677951254
0.0439217409080944
0.04361587496433558
0.043202590936166005
0.04304524828428356
0.04287549470884063
0.04257710973862002
0.042402259453183565

0.042402259453183565
0.04217173980191574
0.041854908770889154
0.04170748843520452
0.04103771076093748
0.04103771076093748
0.040335703198835296
0.03975563515000956
0.03944808336248892
0.03823659625494038
0.03802695380018701
0.03795586904715045
0.03764511811671633
0.036003322026774816
0.03597029898978562
0.03586769960714055
0.035570013883524855
0.03472491036620315
0.03386019144829005
0.03361218856545266
0.0335557886447304
0.033308918740227386
0.03313897268447934
0.03282609893135675
0.032411249922110676
0.032376949781067005
0.03230560573920642
0.03150016722932561
0.031460970515332094
0.03077651057198428
0.030316442058201904
0.029986807771939194
0.029937595599265454
0.029238134378540215
0.029166979975339274
0.028723035891065545
0.028723035891065545
0.028026447209410082
0.027776792689651626
0.02770023282952412
0.027643186445278214
0.02763038935362477
0.02755455476934263
0.027352977875033355

0.027347025209223722
0.027149225446873076
0.02693312327383514
0.02689418317715954
0.026858971747826314
0.026638377591599597
0.026430643665625163
0.02638303780527294
0.02628576530053009
0.02611502203348115
0.02562902640782735
0.025627270163744654
0.025495173770840922
0.02534115645908476
0.025208407116604478
0.025206198920996882
0.02520230666976579
0.02512987157646233
0.025097933338593538
0.024402660866275475
0.024344885504469328
0.024186839952703397
0.024147763708686164
0.02405369501703596
0.02405369501703596
0.023842828163865966
0.023842828163865966
0.02378067784873301
0.02368343935056435
0.023247410030505105
0.022415262710657614
0.02235317604676077
0.022280893997661987
0.022102623228126412
0.022015412174388696
0.021980024189416542
0.021965429949295564
0.021897471334812554
0.021723790467784033
0.021649921700324624
0.02095861095066109
0.020421809239033986
0.020239988887209893
0.020187070060149366

0.02010010718404252
0.02006457058723778
0.02006457058723778
0.019427884142718285
0.01932751116553738
0.0192660596996779
0.018963790016324634
0.0189365373543859
0.018790764375618028
0.018676426547457166
0.018676426547457166
0.01838433359345204
0.01838433359345204
0.01818690939653912
0.018023018861159458
0.017834948330805307
0.01773131731948464
0.017543997047199705
0.017431939572714255
0.017379118219871773
0.017058952598680243
0.016995992500391157
0.01689277381426155
0.016873705135997483
0.016866276791706082
0.016863538716799936
0.01661717663323948
0.01658781986083219
0.015733923453409873
0.015437764165791708
0.015420233709624346
0.014991186662316048
0.014958325179661168
0.014887974742950139
0.0146809312849421
0.0146809312849421
0.014424880271474245
0.014234890762186174
0.014227688864901245
0.013983584611690286
0.013956650082886496
0.013802114135352707
0.013799593993735242
0.013730114694732237

0.013244862552803452
0.01311328022260579
0.013110742061210903
0.012986262075923663
0.012532195232020971
0.012367014595858962
0.012088246674510559
0.012068787592325424
0.01172149189467748
0.011513143747974328
0.011513143747974328
0.011487503266333722
0.011441082932672987
0.011429149393354167
0.011253991486430996
0.011104279922877503
0.011051764962633787
0.01068722074878774
0.01063823044489375
0.010618464633334048
0.0105289307368326
0.010320838858220814
0.010049897560744805
0.009903654217191816
0.009873056997659977
0.009822458000548373
0.009650732898804089
0.009566281835832524
0.009417785994503278
0.009338171006081742
0.009308513605211698
0.00898525726279022
0.008958074448361749
0.008865484831356714
0.00853691088267656
0.008450651556522694
0.008435062894090354
0.00836635585057666
0.008311073805068299
0.008285741728813632
0.008170485316546657
0.007998124990567165
0.007888085554618115
0.007642562906036444

0.00761633427956949
0.007592522706480929
0.0074108409797436665
0.007323272127233523
0.007239888126989034
0.007058531720276585
0.0068376824662822805
0.006621626129320193
0.006530359778448733
0.0065081378691095385
0.0063317321984673386
0.006271845627680777
0.0062500131377544845
0.0061531492169215884
0.006141564486608296
0.0061081670030741
0.006103740287756239
0.005977394722197575
0.005746422858072312
0.005652521713231603
0.005599444474013958
0.005493814649598638
0.005441480205199387
0.0052323197189938254
0.005143635033262875
0.005068839288089746
0.004974015927020563
0.004974015927020563
0.004744925043284016
0.004723967270163921
0.004715581074748292
0.004701635443157985
0.004695355276732606
0.004561903855740903
0.00443450486916463
0.004383841917427603
0.004220473203208139
0.004150104219755144
0.004132323253991296
0.004057805415431727
0.004039652342371665
0.004017881056878875
0.003900399342101013
0.003783814192798303

0.0037748158544336674
0.00372984091096189
0.0036578631432577576
0.0036578631432577576
0.003625094607448104
0.003622106851419804
0.0036174899449636505
0.0035915699733591864
0.0035801022657907214
0.0035586368040789
0.0034282144934690726
0.0034193422261097365
0.003376138481599374
0.003344911281760742
0.0033233394997289312
0.0032809231883625814
0.003253283440081066
0.0032368299072613425
0.0032050927011087727
0.003145999482641381
0.0030435459281625606
0.003014141614658857
0.002989722840038922
0.002950623855611303
0.0029285540415622383
0.0028909024897569103
0.002820471207735539
0.0027406389287303744
0.0026991620357720695
0.002654944705589511
0.0025547506978302963
0.002455147604961792
0.00242198978703906
0.002385781362038834
0.002362419665919883
0.0023604169997533102
0.002233896172453098
0.002160925110189674
0.002147813527188911
0.002134109908509074
0.0020933503417704495
0.0020483958064170574
0.002009751818628051
0.0018866754068743322

0.0018792191178562633
0.0018792191178562633
0.0018518388941874211
0.0018137051186074146
0.001787860393154946
0.0017865563526122908
0.0017818511817639447
0.0017620471883046229
0.0016570518652421426
0.001567497088578041
0.001567497088578041
0.0015520617923354115
0.0015145682717406547
0.0015119900361385465
0.0014199601037749804
0.001347708301979262
0.0013206758748285136
0.001308156545038465
0.0013024302384007569
0.0013000659062238806
0.001280428337750606
0.001278798950287418
0.0012682574352150125
0.0012680713479372417
0.0012573991183060709
0.0012442617799690095
0.0012271575930944568
0.0011825614890943077
0.0011140267942568792
0.0011019702984256968
0.0010865772703517178
0.0010759238948189174
0.0010463702514970537
0.0010157671517493797
0.0009836222015875394
0.0009699161515944696
0.0009696421996114973
0.0009589878034661076
0.0009568495383294111
0.0009568495383294111
0.0009527558685957232
0.0009428590261428003
0.0009297839437754339
0.0009245094087138074

0.0009092962344559379
0.0008954161954906671
0.0008724256898888883
0.0008627949289238179
0.0008238870589158438
0.0008214634062375096
0.0008214634062375096
0.0008195045485375966
0.0008193698581425952
0.000783852467681217
0.0007755541129787131
0.0007741836209400877
0.000765660200374771
0.0007491657027912963
0.0007448107971004574
0.0007420355887501508
0.0007370559545373567
0.0007349728529969174
0.0007349728529969174
0.0007043038693210533
0.0006145094214524545
0.0006035007647405582
0.000601417224702505
0.000587788430205687
0.000563138297384541
0.0005577727152795402
0.0005482657408190134
0.0005421398629297126
0.0005351378733775582
0.0005334838022159703
0.0005257740871380492
0.0004950098483246525
0.0004914489030716823
0.00047642777353759365
0.0004666650123430022
0.00046341374123549483
0.00046184494245839566
0.00041554054853405086
0.00041319009983739716
0.0004008785334577096
0.0003982585673065994
0.00037189381229063817
0.0003714326450918299
0.0003663196352333502

0.00036593330851639704
0.00035433481167898557
0.000353383114673879
0.0003525746003691654
0.00034066863493761537
0.00032924054015742954
0.0003289235442726719
0.000312608054587761
0.00031218985873510894
0.00029950701546381677
0.000296395928122511
0.00028917681769831285
0.0002862295042660314
0.0002782970683175891
0.00027709851343086204
0.00027253309711017124
0.00026198044605567935
0.0002290813320033095
0.00022260308983966838
0.00022260308983966838
0.00022106717181589046
0.0002153609186756865
0.00020776812693088268
0.00020610649688584173
0.0001968133811133528
0.0001968133811133528
0.00019516533489276203
0.00019219449966459277
0.00018902067076301984
0.00018778336485783813
0.00018690488330813649
0.00018592066911482866
0.00018348792233257577
0.0001808634065598439
0.00017785945762313027
0.00017703072870521507
0.0001732224725999168
0.00016897228683976573
0.00016552410258939367
0.00014821269761033665
0.0001480914881219343
0.00014479989563299513
0.00013985729722934307
0.00013180740164628452

0.00013164241100941962
0.00012850117520610393
0.00012534966415883591
0.00012438415748175423
0.00011620438353699391
0.00011004655827361216
0.00010701692448769639
0.00010519495367969596
0.00010368463245721044
0.00010307159018620453
0.00010197263459295135
0.00010089511621738099
9.398559573652808e-05
8.551773949712487e-05
8.321947996582821e-05
8.243434720822454e-05
8.243434720822454e-05
7.908721206565789e-05
7.805586463138777e-05
7.528223358132266e-05
7.037912525369564e-05
7.037912525369564e-05
6.928846273414636e-05
6.709897837562498e-05
6.537756616168174e-05
6.368674616647674e-05
6.300496313583502e-05
6.210046072041839e-05
6.153686794206376e-05
5.9138698760507134e-05
5.817162222927399e-05
5.6453492238757326e-05
5.325970408621023e-05
5.2390316188750006e-05
5.09535866206916e-05
4.915968797056125e-05
4.6339836841254575e-05
4.5718572191381256e-05
4.5060452014236335e-05
4.475865464862433e-05
4.139435714285972e-05
3.7325320562378995e-05
3.405038429830943e-05
3.36322855163941e-05

3.3369303093433806e-05
3.262014059244842e-05
3.213797938255952e-05
2.9318039098299545e-05
2.8166733050447035e-05
2.742835702264918e-05
2.6027353603220885e-05
2.5936475848062234e-05
2.5576449281357496e-05
2.5558607746982373e-05
2.274491288472071e-05
2.1237644613493128e-05
2.010706298160699e-05
1.986783849816921e-05
1.9447500224936677e-05
1.8694155414702003e-05
1.86417378148424e-05
1.8182152539606526e-05
1.8017806871288486e-05
1.6977933398150516e-05
1.647094264632221e-05
1.6171513931653424e-05
1.5889853631729244e-05
1.57754083229768e-05
1.5708390137193818e-05
1.5557831488942715e-05
1.4498754161272376e-05
1.4254182955875553e-05
1.3599366051547731e-05
1.3536196244307363e-05
1.3318936125772042e-05
1.307666831597657e-05
1.2718656047397259e-05
1.2526607664476575e-05
1.1968201612465614e-05
1.1831648501284425e-05
1.1769222066830992e-05
1.1188475684642433e-05
1.068415692620263e-05
9.333168754223669e-06
8.409378785344162e-06
8.310004597462546e-06
7.792166210804149e-06
7.513146824136019e-06

7.171965132300356e-06
7.133972880402221e-06
6.555801993793912e-06
6.0860703648931045e-06
4.960384192313105e-06
4.783743030451857e-06
4.7241202929122805e-06
4.561896912525329e-06
4.527918638323457e-06
4.370367518263299e-06
4.370367518263299e-06
4.177892726484271e-06
3.8084241473006457e-06
3.8056024541485304e-06
3.800831014806367e-06
3.605221201725916e-06
3.605221201725916e-06
3.575723979449836e-06
3.302666914530602e-06
2.982900721450639e-06
2.840180469481325e-06
2.7570035802133963e-06
2.5682803979311264e-06
2.1475898818821395e-06
2.00555369275542e-06
1.9412703671558055e-06
1.9060397153573876e-06
1.8300299740240652e-06
1.805194821017206e-06
1.7487826830567646e-06
1.7449372231377628e-06
1.6135690246642144e-06
1.4406111070526456e-06
1.3329793256487616e-06
1.2979419448234679e-06
1.2755372163611052e-06
1.1505934094806331e-06
1.0965297961797254e-06
1.0687152781305114e-06
1.064373730972366e-06
1.0437304494833474e-06
9.435921758673306e-07
8.882702645092514e-07
8.760977824451337e-07

8.728324992900664e-07
8.269361483708073e-07
8.262804063068709e-07
7.82706397667951e-07
7.796368431233254e-07
7.188756939689151e-07
7.137125532036591e-07
5.811781034175525e-07
5.400777838312859e-07
5.048406448477034e-07
4.698248894436835e-07
4.163953869449516e-07
3.9433014592078226e-07
3.739196482116954e-07
3.438405902674573e-07
3.438405902674573e-07
2.91112707009616e-07
2.877305624168759e-07
2.8477981906931347e-07
1.9975409826045476e-07
1.9460503150500067e-07
1.8983412781404264e-07
1.8825446871969935e-07
1.7941005706120137e-07
1.5823577704641288e-07
1.5449041665902493e-07
1.452998192823923e-07
1.4519649212411882e-07
1.0132926194329208e-07
8.74213183329506e-08
7.835963962250163e-08
6.905895799488163e-08
6.422593223794341e-08
6.281117351690181e-08
6.241079910581932e-08
5.619921310601994e-08
5.2922472720006553e-08
5.19648038142762e-08
5.0056800707224076e-08
4.077835425587527e-08
4.0256541754281844e-08
3.198819011570578e-08
2.9659856805593676e-08
2.847802174066319e-08

2.75215124062199e-08
2.4824000654311992e-08
2.3729965666394254e-08
2.1968500517963135e-08
2.1925848315605864e-08
2.1887434020134346e-08
2.0760871892097858e-08
1.9131905880292465e-08
1.712671840211047e-08
1.6555214463817868e-08
1.572999072959475e-08
1.4794524635572975e-08
1.328877753862196e-08
1.3216103162031201e-08
1.2146646170276243e-08
1.1294652933496113e-08
1.1294652933496113e-08
1.0678648784773411e-08
1.054400275218937e-08
7.642893341486928e-09
7.411370713942333e-09
6.417589744216708e-09
5.12694737551073e-09
4.724292123902546e-09
2.7749455777673196e-09
2.7323969345956744e-09
2.6945858284894046e-09
2.15570500041102e-09
1.988697649957021e-09
1.8757811214383773e-09
1.7107016991674024e-09
1.5972374689960168e-09
1.5165310084984586e-09
1.336364967673194e-09
1.2941734163942283e-09
9.488655765196862e-10
7.097592487803312e-10
6.984455606066735e-10
6.859684636188175e-10
6.430959169353625e-10
5.420136366523274e-10
4.069611739941367e-10
3.9805276392936953e-10
3.9561501889005924e-10

3.1932722361029295e-10
3.0669434162256027e-10
2.769458509213228e-10
2.5572861398767686e-10
2.4368484041553137e-10
2.0927023739396438e-10
2.0270110580993909e-10
2.0093274683038942e-10
1.52847532483193e-10
1.446206705566191e-10
1.1977060440690597e-10
1.1932310299691136e-10
1.1595839179769169e-10
1.0748329085303157e-10
1.0175202636014583e-10
8.675809840245757e-11
6.101870130562521e-11
5.4527624254286645e-11
5.046737699356872e-11
4.988302416777728e-11
4.871966302341672e-11
4.766911572455969e-11
3.74012288984756e-11
3.73484897960182e-11
3.197288080917622e-11
2.3461900158682772e-11
2.2149433497708356e-11
1.6281132440048917e-11
1.3129374247472978e-11
1.1498588429372251e-11
1.1005218517203127e-11
1.0830854612981017e-11
8.523320241203339e-12
8.517448350779138e-12
8.042898126714405e-12
5.9326757639586665e-12
5.059369803568181e-12
3.947638582281734e-12
2.5903039932690806e-12
1.4076880175668913e-12
1.3863285220907292e-12
1.2762656181120433e-12
1.274174272803702e-12
1.2357210758867462e-12

1.0202447829316285e-12
7.199747398712329e-13
5.957009272698122e-13
5.611530469050986e-13
4.589637312829119e-13
4.3096876321890954e-13
3.755562802171378e-13
2.872849050157007e-13
2.4503081058317345e-13
2.276643623387654e-13
1.8434278843689414e-13
1.800623261173997e-13
1.2075763987088306e-13
1.134466271273708e-13
9.427033913790895e-14
5.965765504894414e-14
5.833670029291709e-14
5.067347976530097e-14
4.4860541034237524e-14
2.6821672700698894e-14
1.681818982098083e-14
1.1457942950213189e-14
1.0289079984032642e-14
9.861437886525869e-15
9.628846059749969e-15
7.82716198244938e-15
7.740932971400935e-15
7.291457709448544e-15
6.320205287671162e-15
6.01858396870953e-15
4.9194581985259456e-15
3.707208278151595e-15
3.4121108454440896e-15
2.6664257079132552e-15
1.8931005778234237e-15
1.745625492526497e-15
1.3677620767970957e-15
1.1152101088794093e-15
9.997386112149687e-16
8.632441147715345e-16
3.7352259155334845e-16
3.7330712530610877e-16
2.8525568988805414e-16
2.803608551926427e-16

2.369920745820884e-16
2.190773654035507e-16
2.167153289036663e-16
1.7465114994983466e-16
1.6585490498246377e-16
1.3512916800053436e-16
9.485034193609546e-17
8.215706709291518e-17
6.081862237980565e-17
4.5781790665814054e-17
4.4014365599541236e-17
2.9722767300664254e-17
2.83333234377438e-17
1.2251682716857822e-17
1.2139132807091954e-17
9.082410818691352e-18
6.902706691469217e-18
4.5315403795882094e-18
4.214153585505342e-18
2.0942179445027094e-18
1.576632428561142e-18
1.4793745614299344e-18
7.953612383973414e-19
7.528728627698681e-19
6.452319283919614e-19
4.800040980428532e-19
4.0652351437216992e-19
3.6150621932057274e-19
1.936276801308986e-19
7.650022337157044e-20
7.606906694692152e-20
5.3546415594605463e-20
5.94070259149154e-21
2.153410689669398e-21
1.111840000486067e-21
1.0974212518671064e-21
1.0974212518671064e-21
1.0536352380655171e-21
1.0192002912859257e-21
9.983575893686669e-22
7.8761105396220765e-22
5.046479057840207e-22
4.450450507417832e-22
3.107170298572294e-22

1.25076507405807e-22
7.79694862028404e-23
6.000907633335603e-23
4.515255143923104e-23
3.2977704923838995e-23
3.0953444292296706e-23
1.282208661166127e-23
9.86960787869891e-24
8.079720305858903e-24
5.729271646192917e-24
1.2074289640549188e-24
4.0893873122261544e-25
2.559541368577003e-25
1.3362269861233053e-25
1.224155677143749e-25
1.0600282983561583e-25
4.822885304416875e-26
2.1647148241537147e-26
1.1197159962372072e-26
1.632256972674172e-27
1.5813289006437948e-27
7.480898279544263e-28
3.0598118371656373e-28
1.9066083846114887e-28
1.6303604527128754e-28
1.1030981128728127e-28
9.79796984165723e-29
9.009471473086067e-29
8.14772520444111e-30
1.4743876760486655e-30
1.2894683226286432e-30
2.328830154011718e-31
4.2375978156663164e-32
1.823105541305906e-32
2.0745152730371238e-33
1.2566397240612165e-33
9.719310167481769e-34
6.047290083138718e-34
5.344997702615643e-34
1.6160640372219854e-34
4.3160613851367203e-35
2.000600981479815e-36
1.9770538275645544e-36
1.1656468676186314e-36

1.1656468676186314e-36
8.083738223556015e-37
4.944041708869496e-37
1.3562074768493842e-37
8.606859790979595e-39
7.66579276371539e-39
5.098295543021319e-40
3.8929994611838595e-41
1.9335181013874764e-41
4.91257820050969e-42
2.3252338486334937e-42
7.783742048723094e-43
1.903865846882809e-43
1.4009327076640174e-43
6.055999820708115e-45
1.4330630618264487e-45
3.5311103550828173e-48
3.284016583220781e-48
2.418030728113545e-48
9.906354151359648e-49
9.172527053954933e-49
6.074224761717021e-49
2.3529009495253974e-49
1.5050730040516388e-49
9.906685577976401e-50
1.2041946614688568e-50
1.2471134467164009e-51
2.5328495006878073e-53
1.6321734367638904e-55
9.186882035008799e-56
1.0880865762780227e-56
3.5529314687019277e-57
2.1798819568292597e-58
2.85343626183419e-59
2.841731500372372e-63
6.728366007364775e-66
3.155105237283723e-66
6.754516849303495e-67
1.4312866199244159e-70
9.880704005283662e-73
1.79353030286469e-74
4.162294085661166e-75
8.484019151455363e-77
1.681888319657208e-79

1.1209651365605659e-82
1.1337751783680366e-91
6.074684834393765e-92
5.165608647369021e-95
1.365753692856786e-97
2.4885202262221983e-102
1.257659058922077e-103
2.511880451164759e-110
8.075806279636726e-116
3.718587661930458e-120
8.283370708017509e-123
1.217455187720215e-136
6.382210127329005e-146
4.622680421827724e-148
2.2419685679048736e-151
1.4503551682553877e-161
1.1599680111664957e-170
1.3156036740811774e-184
8.117233991898267e-306
