# Supplementary material for: GFI1 as a novel regulator of γδ T cell development and the IL-17/IFN-γ lineage commitment
Source: Front Immunol. 2026 Aug 5;17:1849622. doi: 10.3389/fimmu.2026.1849622 (PMC13485770; doi:10.3389/fimmu.2026.1849622)
Supplement: Supplementary file 2 [file DataSheet2.pdf]

Column1	Column2	Column3	Column4	Column5
names	scores	logfoldchanges	pvals	pvals_adj
Scart1	13.681422	3.4431906	1.311027537251593e-42	2.4000981124464914e-38
S100a6	11.326073	1.7024804	9.747809445361227e-30	5.948438250540934e-26
Nme2	10.588883	0.9730318	3.3557009411843113e-26	1.5358204282565297e-22
Bcl2	10.430233	1.5022767	1.804456220478399e-25	6.60683600565961e-22
Rplp0	9.933431	0.63059455	2.978285771856552e-23	9.087246270896316e-20
Rpsa	9.8877325	0.6363461	4.7056659592767726e-23	1.2306660959497127e-19
Fau	9.849796	0.46471974	6.868334091198699e-23	1.5717324025946824e-19
Vim	9.576571	1.61137	1.0031990813089337e-21	1.8365565581522648e-18
Eef1a1	9.514198	0.46714592	1.8311958945132955e-21	3.0476093855322635e-18
Rack1	9.402321	0.5516693	5.33724540232212e-21	8.142412631692589e-18
Eef1b2	9.37407	0.66267276	6.978801046756881e-21	9.790214121312535e-18
Jaml	9.3666525	1.974301	7.486917446789506e-21	9.790214121312535e-18
Npm1	9.193327	0.74468267	3.8087686745746183e-20	4.648475208362502e-17
Rplp2	9.120301	0.49096018	7.491566361272076e-20	8.571756585987993e-17
Cd52	9.081114	0.67065716	1.0746980650080257e-19	1.1573233809471721e-16
Ly6a	8.930048	2.1799963	4.2582313904925783e-19	4.330857892541535e-16
Tpt1	8.685915	0.42252055	3.757072086003346e-18	3.6200378251822765e-15
Rplp1	8.422056	0.47979856	3.6993203014740696e-17	3.38617283795429e-14
Dnajc15	8.278737	0.9983545	1.2448882141023978e-16	1.0852461207415523e-13
Eef1g	8.238565	0.7724174	1.7428718724842465e-16	1.4503070622531407e-13
Ccr10	8.122664	5.946815	4.560572050754735e-16	3.478766355548623e-13
Gas5	8.030168	0.91736174	9.733956867556182e-16	6.853828783628885e-13
Lgals1	7.913242	1.122741	2.5077146165440073e-15	1.7003233883359683e-12
Chst11	7.8789177	1.8248156	3.3022897471751194e-15	2.159107800054818e-12
Hopx	7.5290895	1.2579015	5.1095293589096825e-14	3.2255225508123987e-11
Hspa8	7.5052743	0.5607401	6.130005729479675e-14	3.740733829652814e-11
Rbm3	7.3955984	0.9783935	1.4077305441614913e-13	8.31333002321433e-11
Naca	7.302286	0.4557664	2.829188469918418e-13	1.5695137369332265e-10
Btf3	7.1382422	0.5336851	9.453191107596039e-13	4.944559131621734e-10
Adam19	6.6245637	1.7542399	3.4827548769591914e-11	1.7232106360132952e-08
Il18r1	6.5768743	1.1894736	4.804405538564323e-11	2.3145855840657123e-08
Mrpl52	6.5692635	1.0722221	5.05647317713486e-11	2.3735603706104582e-08
Slc25a3	6.5590487	0.7770685	5.415216271733311e-11	2.4784091071655432e-08
Hsp90ab1	6.5138793	0.4336581	7.323430528041301e-11	3.2700010408988315e-08
Capg	6.4741035	2.4684684	9.537663661784485e-11	4.157285920387823e-08
Rala	6.3432326	0.9990158	2.249930264726077e-10	9.153216301408954e-08
Rexo2	6.304868	0.96811044	2.884396330409697e-10	1.147927035235007e-07
Hsp90b1	6.2933426	0.7053193	3.1070157853069767e-10	1.1850028746169755e-07
Ppp1r14b	6.2825985	0.9054661	3.3296035153453456e-10	1.243980643988311e-07
Thy1	6.090489	0.826622	1.1256652883759645e-09	4.040696947901722e-07
Sec61b	6.070504	0.55853164	1.2750940145226731e-09	4.489066562282034e-07
Fam169b	5.992968	0.8414246	2.0604545069242247e-09	7.117120878917317e-07

Snrpf	5.9447746	0.65010524	2.7683747374721882e-09	9.214661148891518e-07
Ndufa4	5.9317203	0.54367626	2.997769133431673e-09	9.80002848673815e-07
Atp5d	5.8811917	0.73742294	4.073231205924373e-09	1.3082218190676753e-06
Arl4c	5.827244	0.7337608	5.6350181411503334e-09	1.7786254674144682e-06
Uqcrh	5.7610655	0.49299484	8.358466481995075e-09	2.5935329811166753e-06
Pdia3	5.7146277	0.7198781	1.0994445417527579e-08	3.3233111466030608e-06
Chchd2	5.6979947	0.48125592	1.2122472357090993e-08	3.5794532490526583e-06
Calr	5.6584873	0.8030991	1.527127884455473e-08	4.437639711226404e-06
Gramd3	5.6406116	0.85328346	1.6944738407262348e-08	4.846989469089872e-06
Eif3i	5.5912337	0.7575395	2.254619920353186e-08	6.350050289523965e-06
Cox8a	5.5761886	0.4707672	2.4584564046239563e-08	6.819236575674359e-06
Lyst	5.562563	0.90270305	2.6584115911725947e-08	7.263812089492044e-06
Nme1	5.5082707	0.81392044	3.62375568440666e-08	9.755896369769517e-06
Nkg7	5.4677973	0.46408117	4.556619830427306e-08	1.2089570903714883e-05
Ostf1	5.4032655	0.58882064	6.543855624683402e-08	1.708616927964117e-05
Eef2	5.401014	0.525495	6.626525475799001e-08	1.708616927964117e-05
Hspe1	5.378047	0.59800255	7.52980840111047e-08	1.9145583666545746e-05
Inpp4b	5.353787	0.89349306	8.613238283910857e-08	2.1600349762130967e-05
Ppp3cc	5.300343	0.82457346	1.1558526343406112e-07	2.8342397931501976e-05
H2-Q7	5.2734447	0.47336268	1.3388647911944978e-07	3.225078648999693e-05
Socs1	5.258887	1.3426149	1.4492997899769397e-07	3.41871310576448e-05
Glipr2	5.2366676	0.9438367	1.6350164422367092e-07	3.7415307510034296e-05
Sntb1	5.218758	1.0553426	1.8012693370968558e-07	4.021443628564895e-05
Atp5g2	5.1922126	0.53836805	2.0780950649299857e-07	4.583576669117259e-05
Krtcap2	5.187735	0.6750754	2.1286702991231989e-07	4.639234186434334e-05
Slc25a4	5.164071	0.8033328	2.416358178588091e-07	5.146986814002845e-05
Vmp1	5.1639533	0.72442985	2.4178776752293913e-07	5.146986814002845e-05
App	5.1184483	2.7447305	3.080596277208713e-07	6.408690459870444e-05
Ddit4	5.082772	1.1125227	3.719668018881393e-07	7.566218046851296e-05
Arhgap12	5.07527	1.064531	3.8694606307587347e-07	7.784419315087929e-05
Cct3	5.050833	0.7620232	4.398871669404472e-07	8.659155231375018e-05
Cox6b1	5.012023	0.5544568	5.386073244358635e-07	0.0001035216713564194
Sell	5.0000105	0.61450803	5.732719796169019e-07	0.00010819474361697549
Camk2d	4.9875946	0.7204212	6.113567763090198e-07	0.00011420518881519618
Il6ra	4.966375	1.5106966	6.821594887309007e-07	0.00012614438141612727
Fbl	4.9308076	0.7760709	8.189034320987178e-07	0.00014843232803397253
Hcst	4.9106717	0.64199895	9.076490473903231e-07	0.0001629052069664181
Emb	4.900776	0.5113262	9.545882026839185e-07	0.00016966646821878152
Snrpe	4.8967605	0.51736397	9.742939197191947e-07	0.00017150383450287787
Ms4a4c	4.894207	1.0522009	9.870295235893487e-07	0.0001720909475080972
Crip1	4.8858905	0.5538447	1.029624280187846e-06	0.00017782388393772545
Tmsb10	4.8234835	0.2225996	1.4107238147427989e-06	0.00023693688877519652
Cox7b	4.8152847	0.58554846	1.469901305110706e-06	0.00024463166538783357
Klk8	4.8084636	0.80217385	1.5209471956695833e-06	0.0002508466694695771

Hspa5	4.7974844	0.6814241	1.606708651701024e-06	0.00026262513648830937
Il7r	4.7700315	0.75212896	1.841970476472542e-06	0.0002957978378314283
Eif3k	4.7583466	0.5470854	1.951851832350357e-06	0.0003086935664746361
Ppib	4.7579184	0.49120232	1.9559979085081e-06	0.0003086935664746361
Dlg5	4.752466	4.966367	2.0095032446573784e-06	0.0003144271444439541
Snx9	4.7473755	1.8270302	2.0607307817258625e-06	0.0003197101561106387
Pgs1	4.704802	0.9615151	2.5411228228411195e-06	0.00038766946264793645
Calm1	4.694948	0.3543076	2.6667400802298804e-06	0.0004001640217112166
Txn1	4.6932263	0.62503135	2.6892967937370287e-06	0.0004002679382353153
Txndc5	4.6884212	1.353546	2.7532079236476746e-06	0.00040647562466304823
Npm3	4.68533	0.7985896	2.7950933591904095e-06	0.00040935819301359064
2410006H16Rik	4.682835	0.7226599	2.829342849434584e-06	0.0004110855519412613
Klrd1	4.653013	0.7075707	3.271189658101103e-06	0.0004678567896160695
Atp5g1	4.6493087	0.65132177	3.33049237485852e-06	0.00046966003348433564
Gpr183	4.649023	0.832032	3.335107027528466e-06	0.00046966003348433564
Kcnq5	4.645201	1.0399123	3.397455103669476e-06	0.0004719068593794324
Ppic	4.6428323	1.308765	3.436656618003192e-06	0.0004719068593794324
Selplg	4.6418576	0.50605625	3.4529076505938836e-06	0.0004719068593794324
Sec61g	4.641782	0.60033524	3.4541715822824024e-06	0.0004719068593794324
Cst7	4.6117086	0.7979496	3.993729175889588e-06	0.0005362028688755254
Satb1	4.6094236	0.43762752	4.037870458586477e-06	0.0005362028688755254
Atp5e	4.609264	0.47703654	4.040971266117105e-06	0.0005362028688755254
Racgap1	4.6066346	1.656021	4.09238262945288e-06	0.0005389874014200998
Mrpl33	4.5966635	0.6352582	4.293106451205972e-06	0.000561384998587341
C1qbp	4.5808787	0.87151545	4.630261099726061e-06	0.0006011786521467022
Ly6e	4.558668	0.43080458	5.14790655169919e-06	0.000663681163675754
Cox4i1	4.555736	0.3862705	5.220239627693264e-06	0.0006683001878614027
Plac8	4.530048	0.80127656	5.89703613563392e-06	0.000739431784486645
Cd8a	4.494951	1.7980078	6.9586071709755365e-06	0.0008549746407989876
S100a13	4.475285	0.6800453	7.630936416484135e-06	0.0009251626024938746
Psmb8	4.4717236	0.4525968	7.759172270397007e-06	0.0009345208339089342
Eif3m	4.443498	0.57051575	8.85079888401159e-06	0.0010521530855168843
Atp5j2	4.436299	0.53256327	9.151879414196879e-06	0.0010809255253916275
Cox5b	4.433997	0.6243426	9.250188052782933e-06	0.0010855332864249817
Eif3f	4.411543	0.45890546	1.0263671026777305e-05	0.0011967963406828797
Serf2	4.4099045	0.28213468	1.0341616408441345e-05	0.001198252984742631
Bax	4.4062505	0.6681651	1.0517536681526559e-05	0.0012109719750233127
H2-K1	4.3854003	0.24628802	1.1577255993331236e-05	0.0013246551591869684
Ndufa6	4.382175	0.5936157	1.175005026991919e-05	0.0013360755918721157
H2aj	4.3805285	0.58880776	1.1839193508598008e-05	0.0013379019479129862
Adgrg5	4.376454	1.5969999	1.2062560775639179e-05	0.0013547809823289964
Tpm3	4.3698177	0.41097024	1.2435030521321087e-05	0.0013880981936208849
Nsa2	4.3569818	0.57514584	1.3186821523914115e-05	0.001463097828110883
Eif3c	4.3035216	0.56522167	1.681045971129727e-05	0.0018428088978126897

Hsp90aa1	4.2835116	0.5858905	1.839665415214622e-05	0.002004687783115124
Eif2s2	4.277917	0.59777784	1.8865045190389465e-05	0.0020435643923104136
Pim2	4.2732043	1.0329338	1.926838372452682e-05	0.002074978240264191
Ptpn18	4.2712803	0.43050864	1.9435375667148457e-05	0.002080721768061326
Atp6v0e	4.269264	0.5060916	1.961186787160309e-05	0.0020874096809618474
Ndufb7	4.266669	0.6173173	1.984135001726866e-05	0.0020996277154111988
Taf10	4.2611413	0.55311996	2.0338569978824034e-05	0.002139874716105354
Txndc17	4.255303	0.73375374	2.087662298268224e-05	0.002183933353965507
Hint1	4.238855	0.5180666	2.2466289992104956e-05	0.0023106200611543
Ssr4	4.2332015	0.6436036	2.303881095568825e-05	0.00235626543109377
Eef1d	4.2112427	0.4851753	2.5396988172453565e-05	0.0025687439915641295
Nop53	4.209294	0.6470377	2.5617028225013583e-05	0.002576763383051229
Epsti1	4.1933327	0.4990355	2.7488569875857263e-05	0.0027349633082462983
Psmb10	4.186999	0.623327	2.826672310769857e-05	0.0027966696138275477
Manf	4.183807	0.6356017	2.8666788618443466e-05	0.0027966696138275477
Hspa9	4.183387	0.76727235	2.8719827792624626e-05	0.0027966696138275477
Eif3h	4.174894	0.46361938	2.981250525068576e-05	0.0028837372072936553
Samd3	4.1728106	0.91785026	3.008651371568734e-05	0.0028837372072936553
Prdx1	4.1666613	0.5206119	3.0909307516717486e-05	0.0029471702745236823
Atp5b	4.1580257	0.533168	3.210098524916095e-05	0.0030292409121463375
Ctsw	4.1543045	0.6586997	3.262786560771952e-05	0.003063170952207801
Sh3bgrl3	4.128809	0.43356514	3.646471390359343e-05	0.0033886269920461163
Tnfsf8	4.104851	0.72222877	4.0457565444455956e-05	0.003721892716540981
Kbtbd11	4.092536	0.6399427	4.266811653842879e-05	0.003886195071985153
Actg1	4.073727	0.33939585	4.626665140171506e-05	0.004151978368682341
Eif3l	4.0709887	0.8419143	4.681400802935688e-05	0.004180605097528958
Fxyd5	4.0474257	0.45459247	5.1784051253018876e-05	0.004579761479657085
Atp8b4	4.0433683	0.55235356	5.268880046194098e-05	0.004637374375272853
Uqcr11	4.0261974	0.68057203	5.668605717543348e-05	0.004926514738307024
Gapdh	4.0258026	0.52858794	5.6781264531751895e-05	0.004926514738307024
Ccr5	4.0012736	1.7813342	6.300248714675427e-05	0.005414960245049908
Tmsb4x	3.995914	0.15573007	6.444512494561518e-05	0.005513069637286809
Psme2	3.9889164	0.5363578	6.637579566971482e-05	0.005625655978358654
Sub1	3.9422016	0.39924237	8.073707389308783e-05	0.006712670829353308
Higd1a	3.939505	0.6620894	8.164988723012225e-05	0.006733173358206522
Cxcr3	3.931424	0.8252392	8.444425745342206e-05	0.00693238126098564
Sfxn1	3.9218304	1.0507406	8.787880177383274e-05	0.00715020988477136
Gabarap	3.917227	0.50332355	8.957337008215167e-05	0.007255839318999781
Atp5l	3.9149086	0.42068875	9.043848196247475e-05	0.0072616547775746715
Nsg2	3.910095	0.59027475	9.225979429901672e-05	0.007375546088349778
Polr1d	3.9050045	0.5018259	9.422366201806956e-05	0.007499793828542606
Pgls	3.8968225	0.73564214	9.746303979418928e-05	0.007724051383169797
Ldha	3.8931513	0.57836527	9.895035513078579e-05	0.007803749079657205
Micos13	3.892244	0.60467726	9.932121787076686e-05	0.007803749079657205

Psmb1	3.8813152	0.45342782	0.00010389309386257918	0.008128080638214689
Cct2	3.8794167	0.5441987	0.00010470726089474102	0.008151575062328047
Klrc2	3.8785431	1.0012114	0.00010508394137266723	0.008151575062328047
Pabpc1	3.876149	0.35830536	0.00010612275536738503	0.008185261087015602
Ust	3.8624897	0.7562125	0.00011223726038340944	0.008561364690996153
Arhgap26	3.8529973	0.5846932	0.00011668068529612932	0.00884246214095041
Hspd1	3.8520732	0.5829695	0.00011712198100173028	0.00884246214095041
Mrps16	3.8515525	0.6068042	0.00011737140439454579	0.00884246214095041
Fgf13	3.830585	0.60334474	0.00012783901156621615	0.009541585983901407
Nhp2	3.8298624	0.83331054	0.0001282148987840578	0.009541585983901407
Pfdn5	3.8272247	0.34807274	0.0001295961875088436	0.009566602438404837
Cyba	3.8108943	0.3818478	0.00013846498507744082	0.010147442521100645
Hs3st3b1	3.8107011	0.9196507	0.0001385732577852822	0.010147442521100645
Ccnd2	3.804913	0.49908406	0.0001418539539741409	0.010268688288990156
Aimp1	3.8048208	0.64029944	0.00014190691929180015	0.010268688288990156
Timm10b	3.8018303	0.6155713	0.00014363115942381282	0.010352187541621028
Rtraf	3.79432	0.57316816	0.00014804852172565187	0.010546008899733499
Gng2	3.7884483	0.87609076	0.00015159113880269063	0.010756507666902547
Cox5a	3.7826772	0.4742303	0.00015515057869618988	0.010966570054792077
Mif	3.7786617	0.5371858	0.00015767341007280624	0.011102027377703322
Mtap	3.7629108	0.9492739	0.00016794702262305872	0.011690517654602038
Cops9	3.7568793	0.6060781	0.00017204534045495333	0.011930431998897085
Il18rap	3.7556279	0.8695822	0.0001729075298275066	0.011944974145479861
Use1	3.7231684	0.6383225	0.00019673816603053626	0.013489459196707966
Cct4	3.7151124	0.4953843	0.0002031133694243892	0.013874613634523483
Eif4a1	3.706796	0.47371653	0.0002098978938833744	0.014284761127594555
Cdk2ap2	3.7007897	0.43560377	0.00021492957434792733	0.014519246190359799
Reep5	3.690709	0.6012101	0.00022362973607431428	0.015051432273207615
Klrc1	3.6860132	0.937448	0.00022779445937490326	0.015221335468523122
Csnk2b	3.6859882	0.6028515	0.00022781700542826982	0.015221335468523122
Mrpl30	3.684686	0.5815366	0.00022898473860064568	0.015243722216589166
Serpina3g	3.655612	1.3569359	0.0002565690711646789	0.016886507793120155
Pik3r5	3.654831	0.68451405	0.0002573515963445962	0.016886507793120155
Emg1	3.6502023	0.51685154	0.00026203394739556055	0.017071371797048138
1700025G04Rik	3.6256225	0.70915526	0.0002882661924601927	0.01859469437966298
Eif3e	3.6224556	0.45077553	0.00029181962422660586	0.018745059160408677
Mrpl24	3.6060412	0.61261886	0.00031090393989693016	0.019828232834022193
Tomm20	3.6051843	0.51698303	0.000311931559305096	0.019828232834022193
Gpr18	3.5916595	0.65931785	0.00032857895058606114	0.020750422375736242
Arhgdia	3.576673	0.5681628	0.00034799486465971835	0.021782291095058918
Txn2	3.5762026	0.6661639	0.00034862136291321697	0.021782291095058918
Farp1	3.5635433	1.1632577	0.00036588238839907955	0.02270579282854898
Chd7	3.560645	0.59083927	0.00036994482968365634	0.022809136928452375
Mrto4	3.5605779	0.9932435	0.00037003952956521304	0.022809136928452375

Larp1	3.5596118	0.57985806	0.00037140334724444461	0.022816379456389514
Cmah	3.5581417	0.5608201	0.00037348774091678944	0.02286769255171794
Setbp1	3.5539331	1.0563936	0.0003795156882499574	0.023159312349306564
Sema4d	3.5373843	0.54879737	0.0004041112512703979	0.02456244063903363
Cct6a	3.5366786	0.5767068	0.00040519238941323844	0.02456244063903363
Ranbp1	3.535511	0.52312106	0.00040698735238047944	0.02458982660075722
Ctla2a	3.520424	0.98130965	0.0004308578178428286	0.025861357610651357
Tbca	3.5192478	0.49413967	0.0004327724284589354	0.02589138839149585
Zfas1	3.5069327	0.807983	0.00045330384518309886	0.027031379458524397
Ndufaf4	3.5038161	0.810074	0.0004586419723393941	0.027260904505250934
1810009A15Rik	3.5019093	0.7231629	0.0004619370224416905	0.02736789990239491
Eif5a	3.492635	0.43954018	0.00047827957397765785	0.02806366718208007
Dpcd	3.4818742	0.9061929	0.0004979176581663517	0.029122615233391054
Limd1	3.4752715	0.6550322	0.0005103367594762024	0.029659476367399486
Atp5c1	3.4518006	0.46436062	0.0005568591725286648	0.03205792726881216
Cct5	3.445265	0.5287442	0.0005704997601328444	0.03274024798981812
Elob	3.4344957	0.4201413	0.0005936576063255993	0.033856977566986746
Eef1e1	3.430161	0.8235562	0.0006032231680023485	0.0341505124676072
Efhd2	3.4299593	0.6041807	0.0006036715500274641	0.0341505124676072
Eif3d	3.4166868	0.64817965	0.0006338818049143927	0.03548768869286785
Prf1	3.4017591	0.9092615	0.000669536221206513	0.037255925840813484
N4bp1	3.400642	0.956095	0.0006722784205770853	0.03729515468334758
Cd44	3.3847818	0.79547244	0.0007123484906788796	0.03916205351008483
Rasal3	3.3824213	0.5512412	0.0007184986028618497	0.03938189797183198
Cycs	3.3802707	0.6146947	0.0007241444593754484	0.039462049845279706
Ola1	3.379708	0.6302474	0.0007256288757378149	0.039462049845279706
Ndufb1	3.3794055	0.5371805	0.0007264276395837254	0.039462049845279706
Nop58	3.3737185	0.6799016	0.0007416017822961434	0.04014778497557809
Gpr15	3.3687117	2.245174	0.0007552035372809479	0.04054402098827658
Timm8a1	3.3641167	1.1746601	0.0007678905444323422	0.04086561685152003
Cd7	3.3625374	0.4584305	0.0007722964748485111	0.04092223753772406
Banf1	3.3550863	0.5004441	0.0007934023873733699	0.04182679221927134
Rsl1d1	3.3544981	0.5909279	0.0007950906042664787	0.04182679221927134
Plek	3.3439977	1.293682	0.0008258042744503659	0.04331804828757263
Mrps34	3.3409567	0.8138492	0.0008349023618856939	0.04367016439726114
Eno1	3.3371346	0.5876785	0.0008464696806909323	0.044149061095182046
Cd69	3.3353117	0.8333383	0.000852038591962895	0.044313268474615676
Lrrk1	3.3317666	1.3011047	0.0008629658679780281	0.044619302670485445
Sem1	3.327558	0.35637307	0.0008761074440407827	0.045053087017007325
Abracl	3.3264828	0.39886072	0.0008794946027420537	0.04510058177142515
Set	3.3255503	0.36892006	0.0008824417291030208	0.04512530931477375
Snhg8	3.3226438	0.5711619	0.000891687116593022	0.045404881265942505
Cox7a2l	3.3222742	0.34001356	0.0008928692443185284	0.045404881265942505
Nacc2	3.3201404	4.5660496	0.000899721790413462	0.04562661168171537

Bsg	3.3179226	0.51858026	0.0009068957582375789	0.045863371950429166
Ubl5	3.3132942	0.42339543	0.0009220398012251055	0.0463855672954871
Nap1l1	3.3132184	0.4854519	0.0009222890968240185	0.0463855672954871
Tmem160	3.3099592	0.53193945	0.000933096081489255	0.04680052044883231
Clta	3.3060865	0.38267142	0.0009460888103833195	0.047275721791717414
Micos10	3.2909825	0.48218933	0.0009983812736820206	0.04953215712004539
Mrpl58	3.2883363	0.61321884	0.0010078135641903518	0.049739160650088245
Ran	3.2878156	0.44124144	0.0010096797670719687	0.049739160650088245
Trmt112	3.28753	0.43080908	0.0010107045262376592	0.049739160650088245
Pan3	-3.2935445	-0.3564141	0.000989326357710702	0.04921629790926582
Dnajc1	-3.3055992	-0.39951983	0.0009477352869154034	0.047275721791717414
Smpdl3a	-3.3310359	-0.70433986	0.0008652347434326941	0.044619302670485445
Nedd4	-3.334354	-1.0611764	0.00085497779052451	0.04434016547062948
Zfand3	-3.3621342	-0.46452376	0.0007734251481975487	0.04092223753772406
Stk38	-3.364814	-0.33947983	0.0007659528084942471	0.04086561685152003
Smc6	-3.3653936	-0.45070344	0.0007643453764693156	0.04086561685152003
Bptf	-3.3699298	-0.31796542	0.000751873225209423	0.04048395039384972
Cdk19	-3.373038	-0.6443655	0.0007434368878964861	0.04014778497557809
Msi2	-3.3922498	-0.4446436	0.0006932120414649254	0.03822479772017587
Asxl2	-3.3991635	-0.44832975	0.0006759232480634769	0.03738406919123284
Plxdc2	-3.407379	-1.1607614	0.0006558997797590195	0.03660840630502552
Mob4	-3.4209037	-0.61647254	0.0006241342091119571	0.035049156338075454
Klhl24	-3.4221218	-0.89163166	0.000621344736306369	0.0349998710386483
Klf3	-3.4296317	-0.3505522	0.0006044008324414011	0.0341505124676072
Hdac9	-3.4415605	-1.1868874	0.000578369291592995	0.033088145691228
Cd9	-3.4550095	-1.205627	0.0005502733771645727	0.03177872150079442
Ogt	-3.4570591	-0.24582498	0.000546104793500006	0.031637786248748766
Sh3kbp1	-3.4765146	-0.20655005	0.0005079764568776071	0.029616321643497936
Pde3b	-3.4926856	-0.3978666	0.00047818931620294784	0.02806366718208007
Cd84	-3.500019	-0.5669706	0.00046522482724371415	0.02747377713661508
Smg6	-3.5256488	-0.48239532	0.00042244670603196297	0.025439907392523508
Cers6	-3.5672395	-1.0992684	0.0003607618135796142	0.022464171840823118
Arhgap15	-3.5904248	-0.1854031	0.00033013965100356813	0.020769300999733065
Baz2b	-3.5915587	-0.5290428	0.00032870609542598515	0.020750422375736242
Scaf8	-3.6186502	-0.57353514	0.0002961436678705795	0.01895630114582762
Fam107b	-3.625446	-0.40932208	0.0002884630580556228	0.01859469437966298
St6galnac3	-3.6407266	-0.6920999	0.0002718698107703428	0.017649363921179665
Slfn5	-3.6530752	-0.94097394	0.00025911834788963883	0.016941712838627207
Ccr9	-3.6576703	-1.1244185	0.00025451824816718246	0.01682117534005996
Trgc2	-3.6663477	-0.5079897	0.0002460391901554724	0.016319708167305193
Zfp827	-3.705452	-1.4895825	0.00021101416125756512	0.0143075416671935
Ncor1	-3.72857	-0.33794084	0.00019256947207967005	0.0132532681404606
Sox5	-3.7643726	-1.5636309	0.0001669677549288773	0.011666712555278461
Auts2	-3.7688165	-2.4373968	0.00016402344314353983	0.011504893385550895

Sesn1	-3.7981927	-0.8743032	0.00014575493294924447	0.01042318577149148
Gpbp1	-3.800318	-0.46780917	0.0001445104576117945	0.010374717441173027
Hivep2	-3.8048124	-0.41323695	0.00014191173524414212	0.010268688288990156
Chd9	-3.827233	-0.7687563	0.00012959176632428495	0.009566602438404837
Trac	-3.849805	-1.3698685	0.00011821184363898347	0.008869279596306847
Chn2	-3.872604	-1.5714808	0.00010767874129139384	0.00824801136745417
Celf2	-3.8754852	-0.28185204	0.00010641241813020775	0.008185261087015602
Jak2	-3.9159417	-0.80629516	9.005197257837688e-05	0.0072616547775746715
Apbb1ip	-3.9260895	-0.2946141	8.633802948843642e-05	0.007056206722521453
Dtx1	-3.9413195	-0.98261994	8.103459077331518e-05	0.006712670829353308
Myb	-3.9699314	-1.3751647	7.1893315432456e-05	0.006009821578182521
Oprm1	-3.9709983	-0.55949986	7.157209087407035e-05	0.006009821578182521
Atf7ip	-3.9735436	-0.41775963	7.081117958646412e-05	0.005973918270458058
Ccm2	-3.991722	-0.5873207	6.559519004467046e-05	0.005585354158826893
Thada	-4.015823	-0.534948	5.92386898389118e-05	0.005115484409815842
Cd3e	-4.0406966	-0.30492982	5.329262950660287e-05	0.004668077360657314
Trgc4	-4.0636973	-0.5145076	4.830148256342959e-05	0.004292501171304396
Crebrf	-4.083472	-0.6731821	4.436781181691752e-05	0.00400118980754832
Rab3ip	-4.0861263	-1.2575821	4.38634956900421e-05	0.003975292156423766
Dock10	-4.0949216	-0.21207763	4.223112287908815e-05	0.0038656258327373345
Pitpnc1	-4.111193	-0.24314234	3.9361951889864686e-05	0.0036393901679179436
Ipcef1	-4.1530695	-0.44079965	3.2804507487849724e-05	0.0030640414213268614
Wls	-4.1649814	-1.0253782	3.1137806964766754e-05	0.00295357425960614
Ssbp3	-4.1736	-1.0027874	2.9982375181354183e-05	0.0028837372072936553
Gimap6	-4.184084	-0.2614458	2.863183378607801e-05	0.0027966696138275477
Airn	-4.1981544	-0.6297492	2.690988328274246e-05	0.0026920176680719464
Cnot2	-4.218677	-0.6348066	2.4574008315175373e-05	0.0024993131679217533
Ripor2	-4.246499	-0.32116872	2.1713655124287325e-05	0.0022458298551430965
Top2b	-4.253757	-0.4525732	2.1021324464510165e-05	0.002186576062339702
Ifi206	-4.312938	-0.7684032	1.6109908578917277e-05	0.001776651182856859
Tnrc6b	-4.4552584	-0.32268417	8.379213319322817e-06	0.0010026029950120446
Chd3	-4.479435	-0.4501752	7.484085122270417e-06	0.0009134076422226968
Cd3g	-4.5159516	-0.27371502	6.303300392850681e-06	0.0007796927046751178
Esyt2	-4.5233274	-0.5817987	6.08749696858166e-06	0.0007581211360804384
Fhit	-4.5308123	-1.3481152	5.875736062977827e-06	0.000739431784486645
Tspan32	-4.551931	-1.3340919	5.315579885552822e-06	0.0006757800067001077
Trat1	-4.6092134	-1.4996568	4.041950942525946e-06	0.0005362028688755254
Nfatc3	-4.653719	-0.44898266	3.260009317971974e-06	0.0004678567896160695
Nrip1	-4.70019	-0.821454	2.599191715798782e-06	0.0003932512623233744
Cbl	-4.732759	-0.55009747	2.214886197211797e-06	0.000340738837078625
2610307P16Rik	-4.787975	-2.009397	1.6847269955835933e-06	0.0002729406823729986
Cdk17	-4.8799515	-0.60999435	1.0611200798729236e-06	0.0001798696787243853
Zfp36l1	-4.881724	-0.6733766	1.051624546250881e-06	0.0001798696787243853
Pde7a	-4.9430723	-0.40936804	7.690095491797352e-07	0.00014078257816833414

Rsrp1	-5.010511	-0.4195997	5.42856855313064e-07	0.0001035216713564194
Rabgap1l	-5.0478344	-0.62706167	4.46846669174232e-07	8.702576566566665e-05
Prkch	-5.061359	-0.48890445	4.1627873148742746e-07	8.283494279717755e-05
Shisa5	-5.103025	-0.31994614	3.3426663820816213e-07	6.875752073794184e-05
Foxn3	-5.1558976	-0.63917637	2.5241896743283065e-07	5.311533375623944e-05
Cd226	-5.2265873	-0.60158336	1.726673945256215e-07	3.902496285901917e-05
Arhgap45	-5.249008	-0.3375214	1.5292044179751146e-07	3.543689275932965e-05
Parp8	-5.2579627	-0.79497576	1.456599236628773e-07	3.41871310576448e-05
Aff3	-5.2995114	-0.5639854	1.1611295378066578e-07	2.8342397931501976e-05
Zbtb20	-5.7134094	-0.5203046	1.1073468069196847e-08	3.3233111466030608e-06
Foxp1	-5.9858446	-0.39210156	2.152701044634141e-09	7.298055189651338e-07
Ikzf2	-6.139203	-0.9816797	8.29365975032691e-10	3.036640580984695e-07
Ets1	-6.295216	-0.41591159	3.0697233480031197e-10	1.1850028746169755e-07
Mllt3	-6.3659897	-0.682592	1.9403456977066378e-10	8.073161065435321e-08
Ift80	-6.4581423	-1.314404	1.0599593896901989e-10	4.51271547606011e-08
Lef1	-6.6751847	-0.530504	2.4692100577060936e-11	1.2556619035118182e-08
Art2b	-7.1515236	-4.7119136	8.582010496405561e-13	4.6209078281675473e-10
Themis	-7.384266	-0.7802803	1.532966703910286e-13	8.770006702651752e-11
Smc4	-8.050186	-0.91486907	8.266837819473057e-16	6.053639998443731e-13
Tox	-8.217489	-1.1123565	2.0780811737729447e-16	1.6540622629678826e-13
Malat1	-9.663214	-0.4283571	4.320923080994324e-22	8.78923764930701e-19
Itga4	-12.587089	-1.9917985	2.4867789694746252e-36	2.2762731297085983e-32
