# Supplementary material for: GFI1 as a novel regulator of γδ T cell development and the IL-17/IFN-γ lineage commitment
Source: Front Immunol. 2026 Aug 5;17:1849622. doi: 10.3389/fimmu.2026.1849622 (PMC13485770; doi:10.3389/fimmu.2026.1849622)
Supplement: Supplementary file 3 [file DataSheet3.pdf]

Column1	Column2	Column3	Column4	Column5
names	scores	logfoldchanges	pvals	pvals_adj
Nme2	7.4976344	0.8577736	6.497977444700702e-14	6.405842767469835e-10
Scart1	7.4879036	2.4199173	6.998244133358645e-14	6.405842767469835e-10
Ly6a	7.01649	1.5652673	2.2751089220615558e-12	1.3883473012060301e-08
Ifngr1	6.9109626	0.85374475	4.813753333628531e-12	2.203134556968438e-08
Lgals1	6.8099766	1.059588	9.761466601455008e-12	2.9783861512139472e-08
Ccr10	6.0410104	1.9237143	1.5315195634318934e-09	4.005361235392524e-06
S100a4	5.616306	0.7714765	1.9508298070550308e-08	4.4642301597195566e-05
Tmem176a	5.3620024	0.88662666	8.230446687919733e-08	0.00016741643057305172
Adam19	5.220578	1.6583704	1.7836522207863558e-07	0.0003265332120593582
Tmem176b	5.19744	0.88405555	2.0205153339115316e-07	0.000336268856526531
Manf	5.1085634	1.055794	3.2461709732712593e-07	0.0004952304333973079
Gimap4	4.9887643	1.5348434	6.076679931223008e-07	0.0008557367653915355
Hsp90b1	4.8810744	0.7663982	1.0550950283764234e-06	0.0013796874774633702
Camk2d	4.829608	1.0102922	1.3680208194230177e-06	0.0016696238094118122
Socs1	4.776412	1.3975801	1.7845073289990001e-06	0.0020418109794990433
Efna5	4.757166	3.8470137	1.963296528416826e-06	0.002114239385042755
Ccr9	4.7195396	1.2992625	2.363791466191985e-06	0.00240410724286537
Bcl2	4.692725	1.2557553	2.695895608231187e-06	0.002597566363152018
Ppp1r14b	4.377008	0.9156111	1.2031957543767766e-05	0.009624516807852946
Cfl1	4.375927	0.51561004	1.2091761980696877e-05	0.009624516807852946
Jaml	4.3424087	1.013155	1.4092897497308839e-05	0.010535438103435523
Ddit4	4.3378677	1.7311238	1.4387171714966302e-05	0.010535438103435523
H2-K1	4.15579	0.27824306	3.2416590806277154e-05	0.021979649181130215
Psmb8	4.0974035	0.68566155	4.178100018897154e-05	0.02684728503710471
Serpina3g	4.093295	2.7296877	4.25286101532767e-05	0.02684728503710471
AW112010	4.0392337	0.8282723	5.3626084791134296e-05	0.03272442447570985
Sdc4	3.9834428	2.154312	6.792405234845207e-05	0.040112439559455225
Tcf7	-4.2777514	-0.5525815	1.887904543950281e-05	0.013293026340806844
Clint1	-4.529028	-0.5162085	5.92556579482395e-06	0.005165682524087717
Malat1	-4.6685057	-0.34367204	3.0339827247467977e-06	0.0027771560870969813
Rsrp1	-6.8666325	-0.8959924	6.573505953118424e-12	2.40682346967478e-08
