# Supplementary material for: GFI1 as a novel regulator of γδ T cell development and the IL-17/IFN-γ lineage commitment
Source: Front Immunol. 2026 Aug 5;17:1849622. doi: 10.3389/fimmu.2026.1849622 (PMC13485770; doi:10.3389/fimmu.2026.1849622)
Supplement: Supplementary file 4 [file DataSheet4.pdf]

Antibodies	Fluorochrome	Clone	Companies
CD117 (c-Kit)	APC	2B8	Biolegend
CD8a	APC-Cy 7	53-6.8	Biolegend
CD3	APC	145-2C11	Biolegend
TCR Vγ3 (Vγ5 Tonegawa nomenclature)	APC	536	Biolegend
TCR Vγ2 (Vγ4 Tonegawa nomenclature)	APC	UC3-10A6	Biolegend
IL22	APC	IL22JOP	eBioscience
CD3	biotin		BD Pharmingen
CD4	biotin	RM4-5	Biolegend
CD8	biotin	53-6.8	Biolegend
CD11b	biotin		BD Pharmingen
CD11c	biotin	N418	Biolegend
Gr1 (Ly6C Ly6G)	biotin	RB6-8C5	Biolegend
CD19	biotin	6D5	Biolegend
TCR γδ	biotin	GL3	BD Pharmingen
NK1.1	biotin	PK136	Biolegend
TCR beta	biotin	H57-507	BD Pharmingen
CD24 (HSA, T cell)	FITC	M1/69	Biolegend
CD8a	FITC	53-6.8	Biolegend
CD44	FITC	IM7	Biolegend
CD45.1	FITC	A20	Biolegend/eBioscience
CD45	FITC	30-F11	Biolegend
TCR Vγ2 (Vγ4 Tonegawa nomenclature)	FITC	UC3-10A6	Biolegend
STAT3Phospho (Tyr705)	AF488	13A3-1	Biolegend
Mouse IgG1, control isotype	AF488		Biolegend
TCR γδ	PE	GL3	Biolegend
CD45.2	BUV395	104	BD Horizon
TCR γδ	BUV395	GL3	BD Horizon
CD117 (c-Kit)	BV421	2B8	Biolegend
CD3	BV605	145-2C11	Biolegend
CD45	BV605	30-F11	Biolegend
CD24	BV605	M1/69	Biolegend
CD27	BV605	LG.3A10	Biolegend
CD4	Pacific blue	RM4-5	Biolegend
CD11b	Pacific blue	M1/70	Biolegend
CD45RB	Pacific blue	C363-16A	Biolegend
TCR Vγ1.1/Cr4 (Vγ1 Tonegawa nomenclature)	Pacific blue	2.11	Biolegend
CD25	AF 647	PC61	Biolegend
CD4	PerCP-cy 5.5	RM4-5	Biolegend
CD44	PerCP-cy 5.5	IM7	Biolegend
CD45.2	PerCP-cy 5.5	104	Biolegend
TCR Vγ2 (Vγ4 Tonegawa nomenclature)	PerCP-cy 5.5	UC3-10A6	Biolegend
RORγt	PerCP-cy 5.5	Q31-378	BD Pharmingen
IFN-γ	PerCP-cy 5.5	XMG1.2	eBioscience
Mouse IgG2a, control isotype	PerCP-cy 5.5		BD Pharmingen
CD4	PE-Cy 7	GK.15	Biolegend
CD25	PE-Cy 7	PC1	Biolegend
CD45	PE-Cy 7	30-F11	Biolegend
IL17A	PE-Cy 7	eBio17B7	eBioscience
Rat IgG2a	PE-Cy 7		eBioscience
c-MAF	PE-Cy 7	sym0F1	eBioscience
c-MAF	eFluor 660	sym0F1	eBioscience
Mouse IgG2b, control isotype	eFluor 660		eBioscience

IL23R	BV421	12B2B64	Biolegend
CD19	BV421	6D5	Biolegend
CD11c	BV421	N418	Biolegend
Rat IgG2b, control isotype	BV421		Biolegend
TCR Vγ6	PE-rat anti-mouse IgM	17D1	Gift from Ira King
